# Type specimens of Neuropterida in the Hope Entomological Collection, Oxford University Museum of Natural History

**DOI:** 10.3897/zookeys.823.30231

**Published:** 2019-02-11

**Authors:** Catherine A. Tauber, Zoë immons, Agatha J. Tauber

**Affiliations:** 1 Department of Entomology, Comstock Hall, Cornell University, Ithaca, NY, 14853, USA University of California Davis United States of America; 2 Department of Entomology and Nematology, University of California, Davis, CA, 95616, USA Cornell University Ithaca United States of America; 3 Hope Entomological Collection, Oxford University Museum of Natural History, Oxford, OX1 3PW, UK> Oxford University Museum of Natural History Oxford United Kingdom

**Keywords:** Neuroptera, Megaloptera, Raphidioptera, type specimens, nomenclature, systematics, lectotype designations, synonymy

## Abstract

The Hope Entomological Collection in the Oxford University Museum of Natural History holds a large and diverse array of historically valuable type specimens for species in the superorder Neuropterida (Megaloptera, Neuroptera, and Raphidioptera). Most are from the mid-1800s (1823–1874). Here, we report each type found during a rigorous search of the collection, and we confirm the identity and status of each type with bibliographic, specimen, and label data. Images, current nomenclatural information, and references for name changes are provided for each species.

We identified primary or secondary types for 76 species in seven families of Neuroptera, primary and secondary types for one species of Raphidoptera (Raphidiidae), and secondary types for three species of Megaloptera (Corydalidae). Among the Neuroptera, we found primary types for 26 species of Mantispidae, 16 species of Myrmeleontidae, 11 species of Ascalaphidae, seven species of Nemopteridae, five species of Chrysopidae, and one species each of Coniopterygidae and Hemerobiidae. Types for only two species that were reported to be in the collection were not found.

To help stabilize the nomenclature, we made new lectotype designations for the following six species in the Hope Collection: *Colobopterusdissimilis* McLachlan, 1871; *Mantispabatesella* Westwood, 1867; *Mantispamyrapetrella* Westwood, 1867; *Mantispatropica* Westwood, 1852; *Myrmeleonanomalus* Rambur, 1842; and *Myrmeleonsingulare* Westwood, 1847. We also made new lectotype designations for two species in the Natural History Museum in London: *Mantispaindica* Westwood, 1852, and *Mantispaquadrituberculata* Westwood, 1852. In several other cases, holotype determinations in the literature were recognized as lectotype designations. Finally, to correct an important error in the literature, we reinstated the synonymization of *Ascalaphusanticus* Walker, 1853, *A.loquax* Walker, 1853, and *A.trux* Walker, 1853 under *A.longus* Walker, 1853.

## Introduction

The Hope Entomological Collection at the Oxford University Museum of Natural History (OUMNH), Oxford, United Kingdom, houses one of the oldest and most historically significant collections of insects in the world. Among the natural history museums in Britain, it is second in size and age only to the national collection of insects at the Natural History Museum in London [NHMUK; formerly the British Museum (Natural History)]. Established through deed of gift in 1860 by Frederick William Hope and curated by John Obadiah Westwood, the Hope Entomological Collection grew rapidly through donation and purchase of materials from various entomologists and collectors, including A. R. Wallace, J. J. Walker, S. A. Neave, and H. W. Bates. Later, entomologists such as J. P. Rambur, L. Navás, and R. McLachlan added specimens and/or worked on the material. As a result, its holdings constitute a rich source of taxonomic history, as well as old and new biological information.

Among the Hope Collection’s holdings are a significant number of type specimens in the superorder Neuropterida, a group of insects that includes the Megaloptera (alderflies, dobsonflies, and fishflies), Neuroptera, *sensu stricto*, (lacewings, antlions, etc.), and Raphidioptera (snakeflies). These specimens include primary types (holotypes, lectotypes, syntypes) from a diverse array of neuropteridan families. In addition to their value as name-bearing representatives of described species, many of these types are of special interest now because of their age (dating from 1823–1939) or association with historical expeditions, or because recent systematic publications have either overlooked or regarded them as lost.

Given the crucial importance of type specimens to the practice of systematics, Recommendation 72F.4 of the International Code of Zoological Nomenclature (ICZN) encourages institutions (collections and museums) to publish lists of the name-bearing (primary) types in their care. Thus, in accordance with the ICZN recommendation, here we provide an annotated and illustrated catalog of the primary and secondary types of Neuropterida that are housed in the Hope Entomological Collection. For each purported type, we provide images of the specimen and its labels, information on its condition and status (holotype, lectotype, syntype, paratype, nontype, etc.), its current name, and a brief taxonomic history of the name.

## Materials and methods

### Format

We made reasonably extensive searches of both the literature and the Hope collection. Our report includes all of the species that we found to have a type in the collection, or for which the literature reports the collection as housing a type or a possible type (Table 1). We are confident that this list includes most, if not all, of the type specimens that were reported to be in the collection or that currently reside there.

**Table 1.** Neuropteridan type specimens reported to be in the Hope Entomological Collection, Oxford University Museum of Natural History. Names appear as in the original descriptions.

Name	Author	Year	Category (Number of specimens)
Megaloptera			
Corydalidae			
1. ***armata*** (*Corydalis*)	Hagen	1861	Paralectotype (1)
2. ***infectus*** (*Nevromus*)	McLachlan	1869	Paralectotype (2)
3. ***subfasciatus*** (*Chauliodes*)	Westwood	1847	Paralectotype (1)
Neuroptera			
Ascalaphidae			
1. ***abdominalis*** (*Suphalasca*)	McLachlan	1871	Syntype (1)
2. ***angulatus*** [Ascalaphus (Ogcogaster)]	Westwood	1847	Syntype (2)
3. ***aurifera*** (*Ulula*)	McLachlan	1871	Paralectotype (2)
4. ***delicatulus*** (*Colobopterus*)	McLachlan	1871	Paralectotype (1)
5. ***dentifer*** [Ascalaphus (Ogcogaster)]	Westwood	1847	Syntype (1)
6. ***dissimilis*** (*Colobopterus*)	McLachlan	1871	Lectotype (New designation), Paralectotype (1)
7. ***leucostigma*** (*Ascalaphus*)	Walker	1860	Syntype (1)
8. ***longus*** (*Ascalaphus*)	Walker	1853	Historical specimen (3)
9. ***macleayanus*** (*Ascalaphus*)	Guilding	1823	Syntype (2 adults, 1 larva)
10. ***mexicana*** (*Ulula*)	McLachlan	1871	Syntype (1)
11. ***obscurus*** [Ascalaphus (Haploglenius)]	Westwood	1847	Syntype (1)
12. ***segmentator*** [Ascalaphus (Ogcogaster)]	Westwood	1847	Syntype (3)
13. ***terminalis*** (*Haploglenius*)	McLachlan	1871	Syntype (1)
14. ***tessellatus*** [Ascalaphus (Ogcogaster)]	Westwood	1847	Syntype (3)
Chrysopidae			
1. ***brevicollis*** (*Hemerobius*)	Rambur	1842	Lectotype
2. ***conformis*** (*Hemerobius*)	Rambur	1842	Lectotype
3. ***mauricianus*** (*Hemerobius*)	Rambur	1842	Holotype
4. ***neavei*** (*Ancyclopteryx*)	Navás	1913	Lectotype
5. ***proximus*** (*Hemerobius*)	Rambur	1842	Syntype (1)
Coniopterygidae			
1. ***detrita*** (*Coniopteryx*)	McLachlan	1867	Holotype
Hemerobiidae			
1. ***fassnidgei*** (*Boriomyia*)	Killington	1933	Holotype
Mantispidae			
1. ***areolaris*** (*Mantispa*)	Westwood	1852	Syntype (1)
2. ***basella*** [Mantispa (Trichoscelia)]	Westwood	1867	Holotype
3. ***batesella*** (*Mantispa*)	Westwood	1867	Lectotype (New designation), Paralectotype (3)
4. ***bella*** [Mantispa (Trichoscelia)]	Westwood	1867	Holotype
5. ***biseriata*** (*Mantispa*)	Westwood	1852	Lectotype
6. ***chilensis*** (*Mantispa*)	Hagen	1859	Syntype (1)
7. ***cognatella*** (*Mantispa*)	Westwood	1867	Lectotype
8. ***crucifera*** (*Mantispa*)	Navás	1914	Lectotype, Paralectotype (3)
9. ***delicatula*** (*Mantispa*)	Westwood	1852	Lectotype, Paralectotype (1)
10. ***eurydella*** [Mantispa (Trichoscelia)]	Westwood	1867	Holotype
11. ***fasciatella*** [Mantispa (Trichoscelia)]	Westwood	1867	Holotype
12. ***fumosella*** [Mantispa (Trichoscelia)]	Westwood	1867	Holotype
13. ***gracilis*** (*Mantispa*)	Rambur	1842	Syntype (1)
14. ***haematina*** (*Mantispilla*)	Navás	1914	Holotype
15. ***hagenella*** (*Mantispa*)	Westwood	1867	Holotype
16. ***hamiltonella*** (*Mantispa*)	Westwood	1867	Syntype
17. ***indica*** (*Mantispa*)	Westwood	1852	Paralectotype (2)
18. ***iridella*** [Mantispa (Trichoscelia)]	Westwood	1867	Paralectotype (1)
19. ***mozambica*** (*Mantispa*)	Westwood	1852	Holotype
20. ***myrapetrella*** (*Mantispa*)	Westwood	1867	Lectotype (New designation), Paralectotype (7)
21. ***natalensis*** (*Necyla*)	Navás	1914	Holotype/Syntype
22. ***nodosa*** (*Mantispa*)	Westwood	1847	Holotype
23. ***partheniella*** [Mantispa (Trichoscelia)]	Westwood	1867	Lectotype, Paralectotype (1)
24. ***quadrituberculata*** (*Mantispa*)	Westwood	1852	Paralectotype (1)
25. ***rubellus*** (*Campion*)	Navás	1914	Lectotype, Paralectotype (3)
26. ***sacra*** (*Necyla*)	Navás	1914	Holotype
27. ***sequella*** [Mantispa (Trichoscelia)]	Westwood	1867	Holotype
28. ***simulatrix*** (*Mantispa*)	McLachlan	1900	Holotype
29. ***tropica*** (*Mantispa*)	Westwood	1852	Lectotype (New designation), Paralectotype (1)
Myrmeleontidae			
1. ***acuta*** (*Acanthaclisis*)	Kimmins	1939	Holotype, Paratype (1)
2. ***anomalus*** (*Myrmeleon*)	Rambur	1842	Lectotype (New designation)
3. ***atomarius*** (*Myrmeleon*)	Rambur	1842	Syntype (2)
4. ***distincta*** (*Acanthaclisis*)	Rambur	1842	Paralectotype (3)
5. ***excelsus*** (*Palparellus*)	Navás	1913	Holotype
6. ***interjectus*** (*Formicaleo*)	Navás	1913	Holotype
7. ***isopterus*** (*Gymnoleon*)	Navás	1913	Holotype (not found)
8. ***loanguana*** (*Creagris*)	Navás	1913	Syntype (1)
9. ***mozambicus*** (*Nelees*)	Navás	1913	Syntype (1)
10. ***neavinus*** (*Formicaleo*)	Navás	1913	Lectotype
11. ***notatus*** (*Myrmeleon*)	Rambur	1842	Syntype (2)
12. ***nycterinus*** (*Palparidius*)	Navás	1913	Holotype
13. ***obscurus*** (*Myrmeleon*)	Rambur	1842	Syntype (2)
14. ***pardus*** (*Palpares*)	Rambur	1842	Syntype (1)
15. ***poultoni*** (*Cymothales*)	Navás	1913	Holotype (not found)
16. ***pulchellus*** (*Myrmeleon*)	Rambur	1842	Holotype
17. ***pulchellus*** (*Palpares*)	Esben-Petersen	1922	Paralectotype (1)
18. ***punctulatus*** (*Myrmeleon*)	Rambur	1842	Syntype (1)
19. ***rhodesicus*** (*Gymnoleon*)	Navás	1913	Holotype
20. ***singulare*** (*Myrmeleon*)	Westwood	1847	Lectotype (New designation)
21. ***tessellatus*** (*Palpares*)	Rambur	1842	Paralectotype (1)
22. ***tillyardi*** (*Acanthaclisis*)	Kimmins	1939	Paratype (1)
Nemopteridae			
1. ***albostigma*** (*Nemoptera*)	Westwood	1874	Holotype
2. ***angulata*** (*Nemoptera*)	Westwood	1836	Holotype
3. ***costalis*** (*Nemoptera*)	Westwood	1836	Holotype
4. ***filipennis*** (*Nematoptera*)	Westwood	1841	Syntype (1)
5. ***hebraica*** (*Nemoptera*)	Westwood	1874	Syntype (1)
6. ***lawi*** (*Croce*)	Navás	1913	Lectotype
7. ***storeyi*** (*Pterocroce*)	Withycombe	1923	Syntype (2)
Raphidioptera			
Raphidiidae			
1. ***bagnalli*** (*Agulla*)	Navás	1914	Lectotype, Paralectotype (1)

For each neuropteridan order, the families and the species within each family are presented in alphabetical order, and, for convenience, we used the traditional classification of neuropteridan taxa. It is worthy of note that recent work has questioned important aspects of the traditional classification, for example, the monophyly of Myrmeleontidae, as well as the monophyly and family status of Ascalaphidae. For recent phylogenetic information, readers are referred to the following selected references (Engel et al. 2018, Winterton et al. 2018; also Badano et al. 2016).

In our text, the format for each species entry is as follows:

**First line**: Species name; author; year of description; original genus name as published (in parentheses); and number of specimens, current type status, and figure number(s) of any types in the collection (in parentheses).

**Section 1** – Original description: Citation for the published description, followed by the exact locality and depository data quoted from the original description.

**Section 2** – Type series: Published data on the number of specimens mentioned or estimated from the literature, notation of published type status, and any other pertinent published information. Then follow details on the type(s) actually found in the collection, their Hope Entomological Collection number(s), pertinent label data, and sex(es) if known, and our interpretation of the current type status (holotype, syntype, etc.), as well as reference to our images of the specimen(s) and accompanying labels. Any other relevant information on the type(s) in the collection or elsewhere (especially the NHMUK) is presented in this section.

**Section 3** – Current name: Presently accepted name of the species.

**Section 4** – Nomenclature: References and information leading to the current acceptance of the name: nomenclatural changes, synonymies, generic reassignments, and spelling issues.

**Note**: (i) We included any explanatory information, translations, or unreferenced comments by the current authors within brackets. (ii) The scale markers on the images apply to the specimens, not necessarily to the associated labels.

To gather or confirm information on selected species with types in both the OUMNH and the NHMUK, we (ZS) made brief visits to the NHMUK. In preparation for these trips, we used the Natural History Museum Data Portal (data.nhm.ac.uk), and the Natural History Museum (2014) Dataset: Index Lot collection, http://dx.doi.org/10.5519/0073880, retrieved: 09 Jan 2018, made available by Ben Price, Senior Curator in Charge of Small Orders. Our coverage of the NHMUK types that overlap with those in the OUMNH was targeted, not exhaustive.

### Type designations

Because the neuropteridan types housed in the Hope Collection are mostly quite old (from the 1800s), the original descriptions are sparse. They seldom indicate the number of specimens that were included in the type series or if a primary type was selected. In some cases, there probably was only one type, but during our studies we learned that it was not prudent to make such an assumption. Not infrequently, we found that type series had been divided, and individual types traded and/or transferred among collections. Therefore, in the absence of specific information from the original description or a published record that indicates a single type, and in accordance with ICZN Recommendation 73F, we assumed that more than one syntype was used. In cases where a previous author had recognized a holotype in the absence of specific evidence of monotypy, and where additional types or probable types had been identified and considered, we noted, in accordance with ICZN Rules 74.5 or 74.6, that the author’s identification of a holotype could be considered as designation of the specimen as the lectotype. In cases where a holotype identification had been referred to in subsequent publication(s), and where no additional types were indicated or had been found, we noted that the holotype had been determined without evidence of monotypy.

We also were aware that the reverse situation might be possible, i.e., that type labels could have been added to nontype specimens either by the original author at a subsequent date or by subsequent authors. We made an effort to compare handwriting and to corroborate the authenticity of each type.

In most cases where several syntypes are known to exist (either in the OUMNH collection or elsewhere), we did not designate lectotypes; we merely indicated the number of syntypes found and their location(s). We trust that the information provided here will aid future systematists in taking the necessary steps to stabilize the type status. In those few instances where we felt that a prompt lectotype designation would be useful for maintaining nomenclatural stability, we made a designation.

### List of type depositories (abbreviations)

**IRSNB**Institut royal des Sciences naturelles de Belgique, Brussels, Belgium (formerly, ISNB or IRSN) [contains the collection of de Sélys Longchamps – "de Sélys collection" here]

**MCZ** Museum of Comparative Zoology, Harvard University, Cambridge, Massachusetts, USA

**MNHN** Muséum national d’Histoire naturelle à Paris, France

**NHMUK**Natural History Museum, London, United Kingdom [formerly, the British Museum (Natural History), BMNH]

**NHRS** Naturhistoriska riksmuseet, Stockholm, Sweden

**OUMNH**Oxford University Museum of Natural History, Oxford, United Kingdom [historically referred to as the Hope Entomological Collection]

## Type specimens of Neuropterida in the Hope Entomological Collection

### Megaloptera

The Megaloptera, one of three orders in the Neuropterida, is a small group that contains only two families: Corydalidae and Sialidae. The larvae are aquatic predators that inhabit streams and other bodies of water, whereas the adults are terrestrial and may feed on pollen, soft or fermenting plant material, or not at all (Villagomez and Contreras-Ramos 2017). We found secondary types (paratype, paralectotype) of three megalopteran species in the Hope Collection; all are in the family Corydalidae.

#### Corydalidae (Dobsonflies, fishflies)

**Figure 1.**
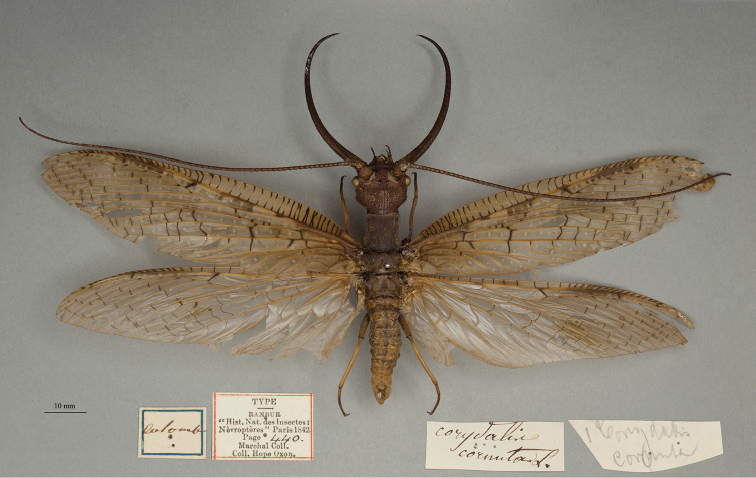
OUMNH paralectotype of *Corydalusarmatus* Hagen, 1861 (NEUR0080, male). Earlier, erroneously identified as *Hemerobiuscornutus* Linnaeus, 1758.

**1. *armata* Hagen, 1861** (*Corydalis*) (Paralectotype; Fig. 1)

**Original description.***Smith. Misc. Coll., 4 (1): 321*; “HAB. Columbia, Venezuela.”; no actual description, but with an indication, i.e., bibliographic reference, to descriptions of *Hemerobiuscornutus* L. by Rambur (1842: 440) and Walker (1853: 208). Sexes and number of specimens not specified.

**Type series.** There is one specimen in the OUMNH labeled as “*Corydaliscornuta* L.”, probably in Rambur’s handwriting; it also bears a printed “TYPE” label that refers to Rambur’s (1842: 440) redescription of the Linnaean species *Hemerobiuscornutus* Linnaeus, 1758. This specimen is not a type of *H.cornutus* L., 1758. The *H.cornutus* holotype, which was from North America, was reported from the Charles De Geer collection of the NHRS (Tjeder 1952), and there is no reason to believe that Linnaeus had seen the OUMNH specimen. However, two subsequent, independent studies redescribed *Corydaluscornutus* (L.) from Colombia and Venezuela, not from North America, as was the original description by Linnaeus (Colombia: Rambur 1842: 440; Venezuela: Walker 1853: 208; both as *Corydaliscornuta*). Rambur’s label on the specimen and the reference to the Marchel Collection on another label are indicators that this specimen in the OUMNH (NEUR0080, Fig. 1) is one that Rambur used in his redescription.

*Corydaluscornutus* (L.), originally described from Pennsylvania, USA, is widespread and relatively common throughout southeastern Canada and eastern and central USA. Its distribution was later shown to extend into southwestern USA, but it has not been recorded from Mexico, Central America, or South America (Contreras-Ramos 1998: 82). As a result, Rambur’s and Walker’s redescriptions based on their South American specimens now are considered applicable to a separate species, *Corydalusarmatus* (Hagen, 1861) (van der Weele 1910: 17, Contreras-Ramos 1998: 48, 82–84). Indeed, the specimens they used to prepare their redescriptions constitute the type series for this South American species, because in lieu of a description, Hagen (1861: 321) referred to Rambur’s and Walker’s earlier redescriptions of the Colombian and Venezuelan specimens that they had identified as *C.cornuta*. According to ICZN Article 72.4, the type specimens mentioned in Rambur’s and Walker’s publications thus serve as types for Hagen’s South American *Corydalisarmata* [= *Corydalusarmatus*] (Contreras-Ramos 1998: 48). Contreras-Ramos (1998: 51) designated a male from the de Sélys collection (IRSNB) as the *C.armatus* lectotype, and he identified paralectotypes from a number of collections, but not the OUMNH.

Here, the OUMNH specimen, a male (Fig. 1, NEUR0080), is recognized as a paralectotype of *Corydalisarmata* Hagen. However, it should be noted that there is at least one other *Corydalus* species from northern South America, so the actual species identity of this specimen has not been confirmed.

**Current name.***Corydalusarmatus* (Hagen, 1861).

**Nomenclature.**Linnaeus (1758: 551) originally included the North American species (*cornutus*) within his broadly defined genus *Hemerobius*. The genus name “Genre. Corydale; *corydalus*” [masculine] first appeared in Latreille (1802: 290), with *Hemerobiuscornutus* F. (sic) listed as the type species. A short time later, Latreille (1805: 44) referred to the genus as “Corydale; *corydalis*” [feminine], with “la raphidie cornue de Linnaeus” and “l’hémerobe cornu de Fabricius et de De Géer” as examples. ICZN Article 33.2 (Emendations) does not apply to this change, and thus the name is considered to be an incorrect subsequent spelling and unavailable. Latreille (1807: 199) again used the name *Corydalis*; however, with this usage he provided a list of synonyms under the name *Corydaliscornuta*, including some in combination with the original genus name (*Corydalus*). A second author, Rambur (1842: 440–441), also used the name *Corydalis*; his usage was in association with references to several published articles pertaining to species under the original name. Both authors attributed the name *Corydalis* to Latreille. These uses of the name are deemed unjustified emendations, and they render *Corydalis* Latreille, 1804 an available name and junior synonym of *Corydalus* Latreille, 1802 (see ICZN Article 33.2). Subsequently, the name *Corydalis* was commonly used (e.g., Walker 1853: 208, others listed by Contreras-Ramos 1998). However, it was not in exclusive use (e.g., van der Weele 1910: 9), and today the original generic appellation *Corydalus* Latrielle, 1802 is regarded as the valid name. For a list of generic synonymies and references to species combined with both generic names, see Contreras-Ramos (1998: 29) and references therein.

Hagen (1861: 321) first reported the species name as “armata”; he listed it under the feminine genus name “*Corydalis* Latreille”. He immediately followed that listing with the name “*Corydalisarmatus* Hagen”. It appears that the masculine spelling “*armatus*” was a *lapsus calami* by Hagen or a printer’s error, because all of the other species that Hagen listed in this publication under the genus name *Corydalis* have feminine endings. The validity and history of the combination *Corydalusarmatus* Hagen were examined and confirmed by Contreras-Ramos (1998: 29).

**Figure 2.**
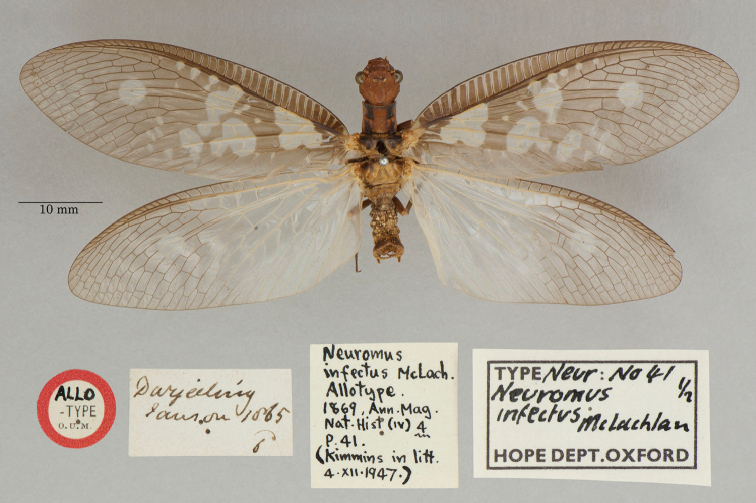
First of two OUMNH paralectotypes for *Nevromusinfectus* McLachlan, 1869 (NEUR0041-01, male).

**Figure 3.**
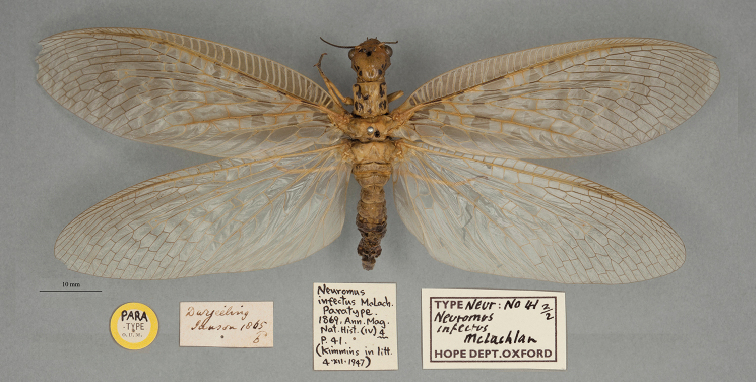
Second of two OUMNH paralectotypes for *Nevromusinfectus* McLachlan, 1869 (NEUR0041-02, female).

**2. *infectus* McLachlan, 1869** (*Nevromus*, as *Neuromus*, see below) (Two paralectotypes; Figs 2, 3)

**Original description.***Ann. Mag. Nat. Hist., 4: 41*; “… (♂ ♀)…. Darjeeling. In coll. Mus. Brit., Oxon., et auct.”. Number of specimens not specified.

**Type series.** Although McLachlan’s description did not state how many specimens he had in the type series, clearly there were more than two and at least one of each sex. He did not specify a primary type. We have seen specimens in the NHMUK, and two specimens are in the OUMNH. Each of the OUMNH specimens bears a locality label reading “Darjeeling” and “1865” (NEUR0041-01, male, Fig. 2; NEUR0041-02, probably a female but labeled as a male, Fig. 3).

Quite a few years after the description was published, van der Weele (1910: 37) made the following ambiguous statement: “Mac Lachlan’s *infectus* is a very mature ♂ of this species from Darjeeling, the type is also in the British Museum”. This statement neither restricted the type series to the specimens in the NHMUK, nor did it identify a specific specimen as the primary type. Thus all of the available types retained name-bearing (syntype) status. Subsequently, Kimmins (1948: 780) reported three type specimens in the NHMUK, all females; he identified one (the only one of the three females bearing a type label) as the holotype, the other two types in the NHMUK as paratypes, and the two specimens in the OUMNH as an allotype (♂) and a paratype (♀). Because Kimmins expressly chose the holotype from among the available syntypes, his action constituted a valid lectotype designation (ICZN Article 74.5; also see Oswald 2018). The two types in the OUMNH are paralectotypes.

Note: In an article over 20 years later, Kimmins (1970: 355), without reference to his own earlier article, accepted that van der Weele's (1910: 37) statements established the lectotype. However, for the reasons above, and unless a large male specimen from Darjeeling were found in the NHMUK to contradict Kimmins' (1948: 780) sex determinations, we disagree and conclude that Kimmins' earlier holotype identification served as the valid lectotype designation.

**Current name.***Protohermesinfectus* (McLachlan, 1869).

**Nomenclature.** The original generic name *Neuromus* is recognized as an unjustified emendation of *Nevromus*, probably used for the first time by Hagen (1861: 194). The species was synonymized with *Protohermesanticus* (Walker) by van der Weele (1907: 244, as *Neuromus*), but later reinstated as a valid species within *Protohermes* by Kimmins (1948: 778); also see Liu and Yang (2006: 51).

**Figure 4.**
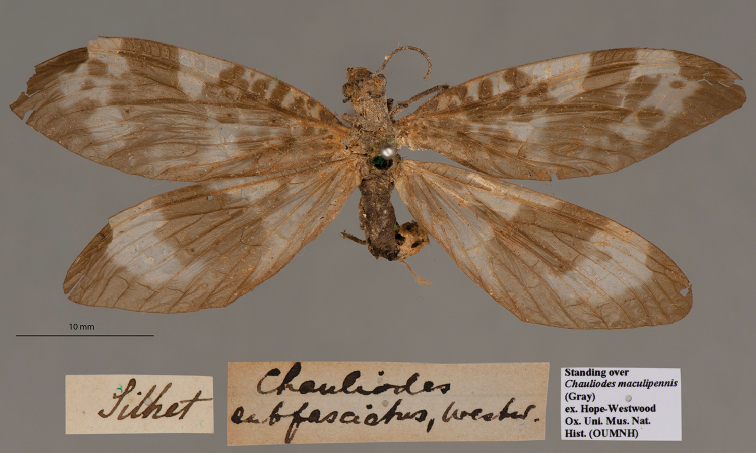
OUMNH paralectotype of *Chauliodessubfasciatus* Westwood, 1847 (NEUR0072, female).

**3. *subfasciatus* Westwood, 1847** (*Chauliodes*) (One paralectotype; Fig. 4)

**Original description.***The Cabinet of Oriental Entomology; being a selection of the rarer and more beautiful species of insects, natives of India and the adjacent islands. The greater portion of which are now, for the first time, described and figured. Smith, London, 1848 [1847]: 70, fig. 5.* “Inhabits Sylhet. In the Collection of W. W. Saunders, Esq.”. Sexes and number of specimens not specified.

**Type series.**Walker (1853: 200) reported two types of this species in the NHMUK; he did not specify the condition or sexes of these types. Later, van der Weele (1910: 65–66) stated that he examined two male types with “abdomen … broken off” in the NHMUK and a female type in the de Sélys collection. Kimmins (1970: 358) designated one of the NHMUK specimens as the lectotype and the other as a paralectotype (he also noted the absence of their abdomens). For additional information, see Liu et al. (2010: 44).

Because the OUMNH holds types of other species from the W. W. Saunders collection that were described by Westwood (1848) from the Cabinet of Oriental Entomology, we expected to find a type of this species in the collection as well. Indeed, we found one (NEUR0072, Fig. 4), a female, now identified as a paralectotype; it bears a “Silhet” locality label and previously was housed in the OUMNH collection with *Chauliodesmaculipennis* (Gray).

**Current name.***Neochauliodessubfasciatus* (Westwood, 1847).

**Nomenclature.** An article by van der Weele (1909: 259) described the genus *Neochauliodes*, but it did not list *C.subfasciatus* as an included species. However, shortly thereafter, another article by the same author (van der Weele 1910: 65) appeared with the species listed as *Neochauliodessubfasciatus* (Westwood), without comment. We presume that this was the first use of the combination.

### Neuroptera

Neuroptera, with its approximately 16 described families, is by far the largest and most diverse of the neuropteroid orders (Withycombe 1924, Henry 1982, Tauber et al. 2002, Engel et al. 2018). For the most part, the larvae are predaceous; in some families adults also may be predaceous, but, more commonly, they feed on honeydew and pollen. The taxa within the order express a broad range of diversity in many respects, including their lifestyles (arboreal, subterranean, aquatic, semiaquatic); defense strategies (larval trash carrying, chemical repellents, mimicry); reproductive biology (secondary sexual characteristics, courtship songs and movements, chemical attractants); seasonality (diapausing stage, voltinism, ecophysiological responses); and larval food relations (predation or semiparasitoidism, prey preferences, searching and feeding behavior).

The OUMNH contains types from seven of the ~16 neuropteran families. Types of only two species that were reported to be in the OUMNH were not found; both were antlions (Myrmeleontidae) described by L. Navás in 1913.

#### Ascalaphidae (Owlflies)

About 450 species of owlflies are known from the warm regions of the world. They are very closely related to the antlions (family Myrmeleontidae), but differ noticably in the length and structure of their adult antennae, larval head morphology, and flight pattern (some species).

The OUMNH houses type specimens of thirteen ascalaphid species, eleven with primary types (holotype, lectotype, or syntype). It also holds secondary types (paralectotypes) of two species, and historical specimens of an additional species (also see the Appendix). All of the species with OUMNH types were described by Westwood, McLachlan, Guilding, and Walker, between 1823 and 1871. They include seven species from the Old World: the East Indies, Gabon (Gaboon), and six from the New World: Brazil, Mexico, and the Caribbean. Five of the New World types were collected by H. W. Bates during his expedition in Brazil's Amazon region (1848–1859). All five of the specimens carry labels with the year "1861", probably the year that they were transferred to the OUMNH.

It is noteworthy that ten of the eleven primary types of Ascalaphidae in the OUMNH are syntypes; only one is a lectotype, and none are holotypes. The cause of this skewed distribution is not readily apparent.

**Figure 5.**
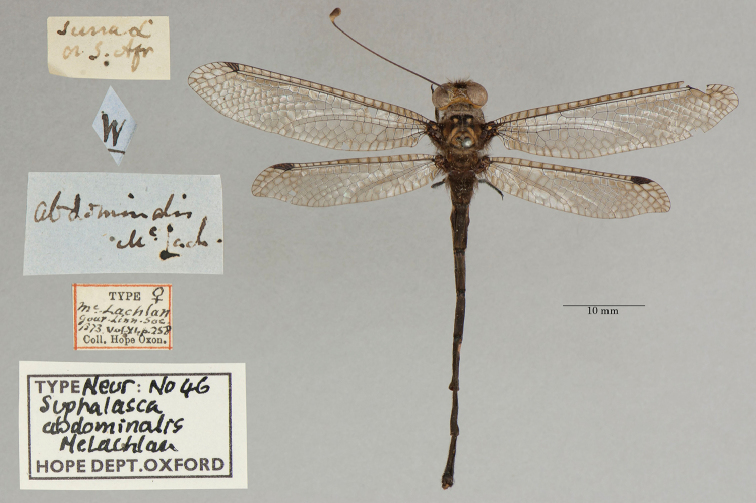
OUMNH syntype of *Suhpalacsaabdominalis* McLachlan, 1871 (NEUR0046, female).

**1. *abdominalis* McLachlan, 1871** (*Suphalasca*, a subsequent spelling of *Suhpalacsa*) (One syntype; Fig. 5)

**Original description.***J. Linn. Soc. Lond., Zoology, 1873a [1871], 11: 258*; “*Hab.* Gaboon. One ♂ in my collection. A second example in the Oxford Museum, perhaps a ♀, ...”.

**Type series.** McLachlan mentioned two specimens: one in his own collection (a male) and another (perhaps a female) in the OUMNH. Although it appears that the description largely refers to the male, McLachlan did not specify a primary type.

A single type specimen, labeled as a female (unconfirmed), is in the OUMNH (NEUR0046, Fig. 5). The NHMUK houses a specimen from Gabon that is labeled as a type (BM1938674); we did not examine it closely. No lectotype has been designated, so these specimens both remain as syntypes.

**Current name.***Suhpalacsaabdominalis* McLachlan, 1871.

**Nomenclature.** First, the original spelling of the genus that Lefèbvre (1842: 7) described, and to which *S.abdominalis* was assigned, is *Suhpalacsa*, which is “*Ascalaphus*” spelled backwards. McLachlan (1873a [1871]: 253), as well as Navás (1912a: 84, 86–88), used other spellings, *Suphalasca* and *Suphalacsa* respectively. The type label on the OUMNH specimen also carries the spelling *Suphalasca*. McLachlan’s subsequent spelling is not demonstrably intentional; thus, it is not viewed as an emendation. However, Navás consistently adopted his subsequent spelling in favor of others that he listed. But notably, he did not cite the original spelling, and therefore his spelling appears to be an unjustified emendation. Moreover, it has not been in prevailing usage; for example, Tjeder (1992: 165) and Ghosh (2000: 102) used the correct original spelling. Therefore, ICZN Article 33.2.3.1 does not apply; Navás’ emendation is unjustified; and *Suphalacsaabdominalis* McLachlan remains as an available junior synonym of the original name.

Second, in the original description, McLachlan (1873a [1871]: 258) was uncertain about the placement of this and other African species in *Suhpalacsa* (as *Suphalasca*). He used the genus name for his new species, and listed it with a “(?)”. Later, Tjeder (1992: 164–165) also questioned the generic assignment of the species. He proposed that it should be the type species of a separate genus. Unfortunately, Tjeder’s health failed before he could describe the new genus, but his opinion was made clear by the colleagues/editors who helped shepherd his final manuscript through publication.

**Figure 6.**
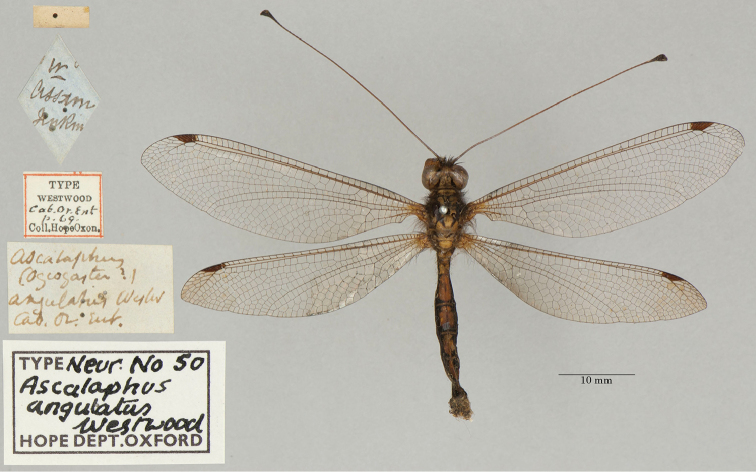
First of two OUMNH syntypes for Ascalaphus (Ogcogaster) angulatus Westwood, 1847 (NEUR0050-01, sex undetermined).

**Figure 7.**
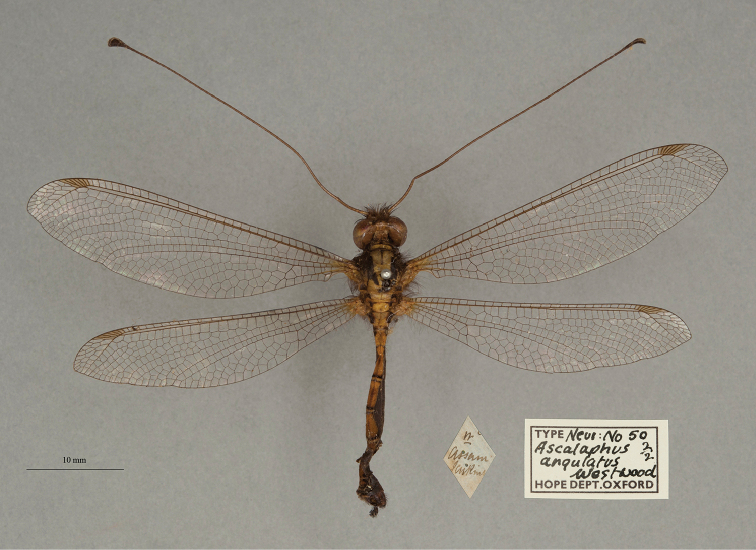
Second of two OUMNH syntypes for Ascalaphus (Ogcogaster) angulatus Westwood, 1847 (NEUR0050-02, sex undetermined).

**2. *angulatus* Westwood, 1847** [Ascalaphus (Ogcogaster)] (Two syntypes; Figs 6, 7)

**Original description.***The Cabinet of Oriental Entomology; being a selection of the rarer and more beautiful species of insects, natives of India and the adjacent islands. The greater portion of which are now, for the first time, described and figured. Smith, London, 1848 [1847]: 69*; “Inhabits Assam. Major Jenkins.”. Sexes and number of specimens not specified.

**Type series.**Walker (1853: 421) reported a Westwood type from Assam in the NHMUK, and two Westwood types (both from Assam, sexes undetermined) are in the OUMNH (NEUR0050-01, -02; Figs 6, 7). No lectotype has been designated; we consider all three specimens (including the two in the OUMNH) to be syntypes.

**Current name.***Ascalohybrisangulata* (Westwood, 1847).

**Nomenclature.**Hagen (1866: 381) referred to the species as *Ogcogasterangulatus* Westwood. Later, McLachlan (1873a: 267) transferred the species to the genus *Hybris* Lefèbvre; this generic assignment was used by van der Weele (1908: 236). Subsequently, Oswald and Penny (1991: 30) recognized *Hybris* Lefèbvre as a junior homonym, which Sziráki (1998: 59) replaced with the generic name *Ascalohybris* Sziráki. Sziráki (1998: 59) also was the first to use the combination *Ascalohybrisangulata* (Westwood). Ghosh (2000: 108) probably did not see Sziráki’s paper and continued to use the homonym.

**Figure 8.**
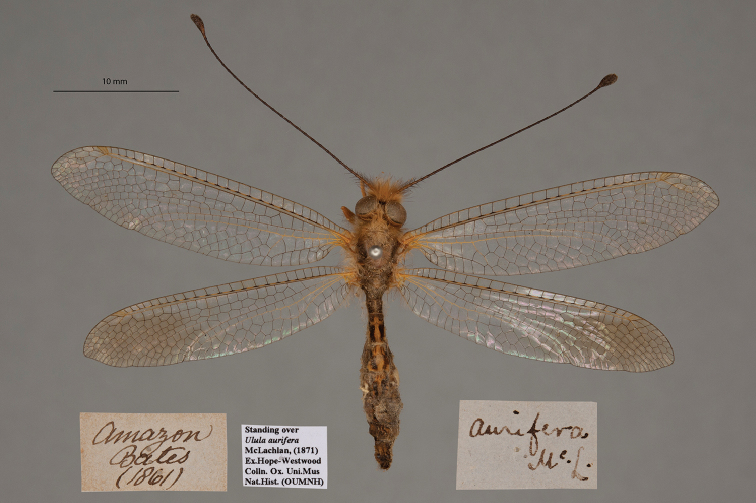
First of two OUMNH paralectotypes for *Ululaaurifera* McLachlan, 1871 (NEUR0077-01, sex undetermined).

**Figure 9.**
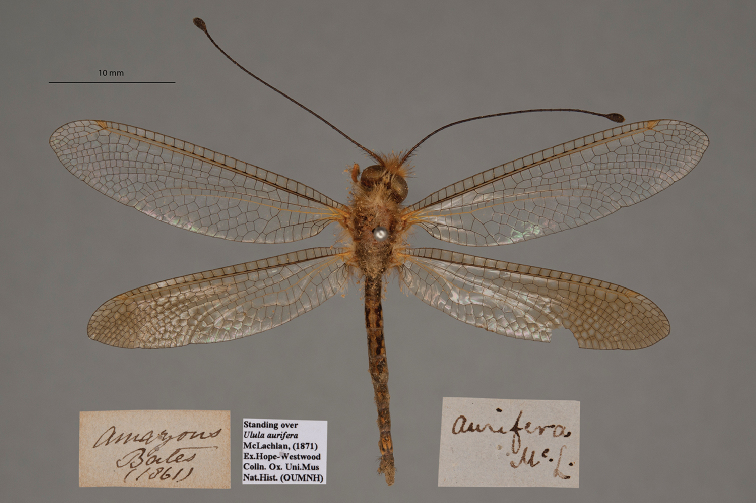
Second of two OUMNH paralectotypes for *Ululaaurifera* McLachlan, 1871 (NEUR0077-02, sex undetermined).

**3. *aurifera* McLachlan, 1871** (*Ulula*) (Two paralectotypes; Figs 8, 9)

**Original description.***J. Linn. Soc. Lond., Zoology, 1873a [1871] 11: 249*; “Santarem (Bates). In the British and Oxford Museums.”. Sexes and number of specimens not specified.

**Type series.** McLachlan mentioned two depositories, and we conclude that there were at least two syntypes. However, he did not identify a primary type. Both van der Weele (1908: 120) and Penny (1981b: 648) stated that they saw a type in the NHMUK; Penny referred to it as a “holotype female”. Thus, in accordance with ICZN Rule 74.5, the reference by Penny (1981b: 648) to the NHMUK specimen as the holotype serves to fix it as the lectotype. This type, with a McLachlan label (sex unconfirmed by us), carries an NHMUK identification label (NHMUK010212092).

In addition to the specimen above, we found three specimens, sexes undetermined, in the OUMNH standing over this name. Two of them bear labels showing the type locality and McLachlan's identification; they clearly are paralectotypes (NEUR0077-01, -02; Figs 8, 9). The third does not appear to be a type.

**Current name.***Ululodesvetulus* (Rambur, 1842).

**Nomenclature.** Both van der Weele (1908: 119) and Penny (1981b: 648) listed *Ululaaurifera* McLachlan as a synonym of *Ululodesvetula* (Rambur). In accordance with ICZN Article 30.1.4.4, the suffix “-odes” is treated as masculine; thus, the species name is the masculine “*vetulus*”.

**Figure 10.**
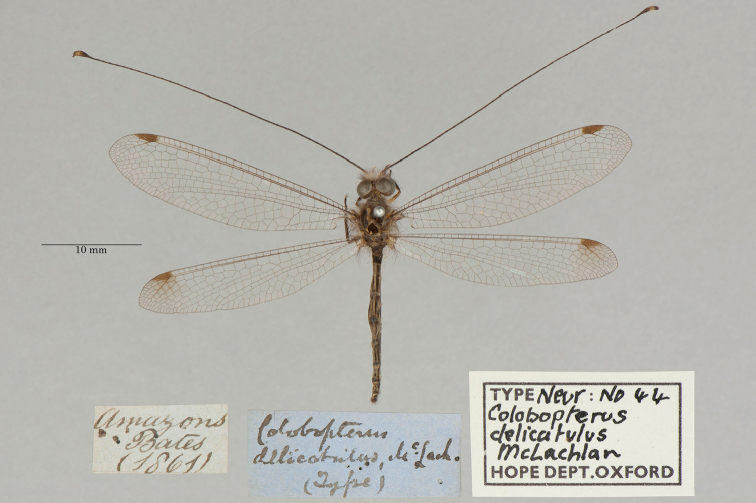
OUMNH paralectotype of *Colobopterusdelicatulus* McLachlan, 1871 (NEUR0044, male).

**4. *delicatulus* McLachlan, 1871** (*Colobopterus*) (One paralectotype; Fig. 10)

**Original description.***J. Linn. Soc. Lond., Zoology, 1873a [1871], 11: 250*; “*Hab.* Santarem (*Bates*).”. Sexes and number of specimens not specified.

**Type series.** Although McLachlan (1873a: 250) neither identified a primary type nor mentioned the sexes or number of specimens he studied, he did note that *Colobopterusinteger* McLachlan actually might represent the female of *C.delicatulus*. [See his paragraph on page 251, immediately after the *C.delicatulus* description]. With this statement, he implied that at the time he wrote the *C.delicatulus* description, he may not have identified a female specimen of this species with certainty, in which case the description most likely was based on a male (or males). The first subsequent author to report on the topic, van der Weele (1908: 132), was aware that McLachlan believed he had studied a male. However, he stated, probably in error: “The type is a female, not a male, as Mac Lachlan said, from Santarem (Bates). I examined the type in the British Museum.” [translation from Oswald 2018]. Later, Esben-Petersen (1927: 345) also mentioned a female specimen, but he did not refer to a type. Finally, Penny (1981b: 630) referred to the specimen in the NHMUK as the “Holotype female” (also see Oswald 2018). This female type is in the NHMUK (NHMUK010212094). It carries a locality label reading "Santarem" that is in Bates’ handwriting and a label stating “Type”.

Here, we report that the OUMNH also holds a *C.delicatulus* type (a male, NEUR0044, Fig. 10). It carries a type label probably written in McLachlan’s handwriting. Now, given the discovery of the type in the OUMNH, Article 74.5 applies and the NHMUK female specimen that van der Weele and Penny considered to be the holotype becomes the lectotype. The specimen in the OUMNH is identified as a paralectotype.

**Current name.***Ameropterusdelicatulus* (McLachlan, 1871).

**Nomenclature.** The original genus name *Colobopterus* Rambur was identified as a junior homonym (Esben-Petersen 1922: 621). *Ameropterus* Esben-Petersen is the replacement name.

**Figure 11.**
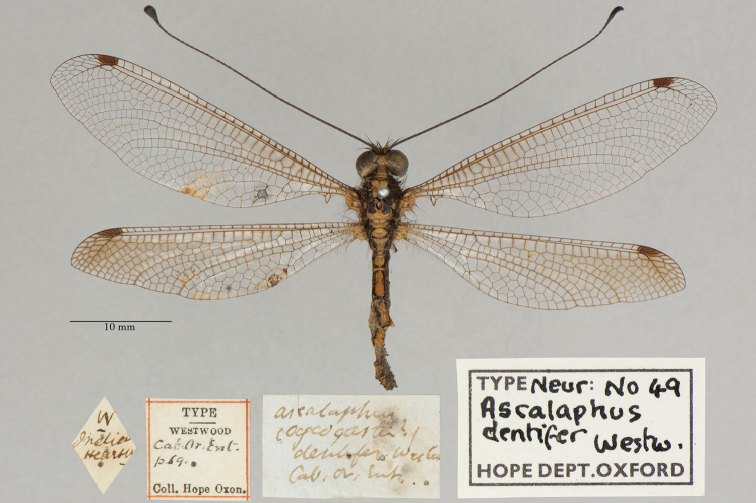
OUMNH syntype of Ascalaphus (Ogcogaster) dentifer Westwood, 1847 (NEUR0049, sex undetermined).

**5. *dentifer* Westwood, 1847** [Ascalaphus (Ogcogaster)] (One syntype; Fig. 11)

**Original description.***The Cabinet of Oriental Entomology; being a selection of the rarer and more beautiful species of insects, natives of India and the adjacent islands. The greater portion of which are now, for the first time, described and figured. Smith, London, 1848 [1847]: 69*; “Inhabits the East Indies. Col. Hearsey.”. Sexes and number of specimens not specified.

**Type series.**Walker (1853: 421) reported a specimen at the NHMUK; it was from the “East Indies” and originally in the collection of Mr. Stevens. Later, van der Weele (1908: 241, fig. 197) reported seeing “Typen” in London, and he provided an image of one of the specimens. Fraser (1922: 516) also reported a type in the NHMUK. Apparently, there have been no reports of the type in the OUMNH since the original description.

We found a syntype of *A.dentifer*, sex undetermined, in the OUMNH (NEUR0049, Fig. 11); its labels confirm it as one of Westwood’s syntypes from the Cabinet of Oriental Entomology. We also found in the NHMUK the specimen that was shown in van der Weele’s image (a female, NHMUK010212096). Its labels too are in Westwood’s handwriting, and we consider it to be a second syntype. Other specimens are associated with this syntype in the NHMUK; their type status should be evaluated.

Thus, there are syntypes in the OUMNH and NHMUK. No lectotype has been designated.

**Current name.***Glyptobasisdentifera* (Westwood, 1847).

**Nomenclature.**Hagen (1866: 382) referred to the species as *Ogcogasterdentifer* Westwood. The current combination was first used by McLachlan (1873a: 268) and has remained unchanged since then.

**Figure 12.**
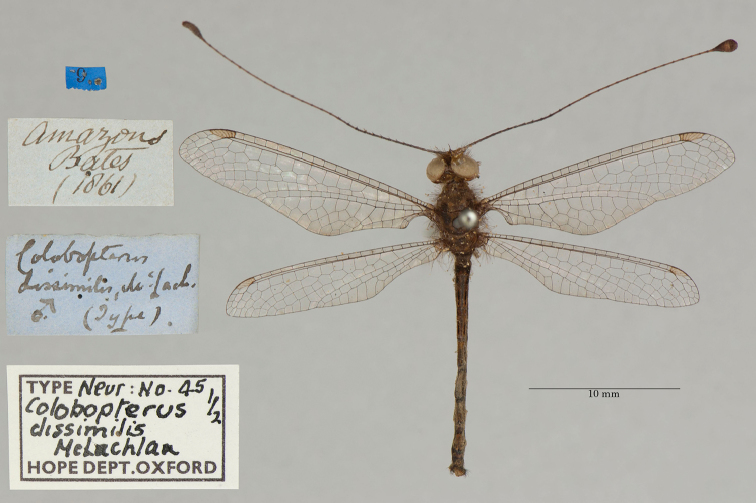
Lectotype (present designation) of *Colobopterusdissimilis* McLachlan, 1871 (NEUR0045-01, male).

**Figure 13.**
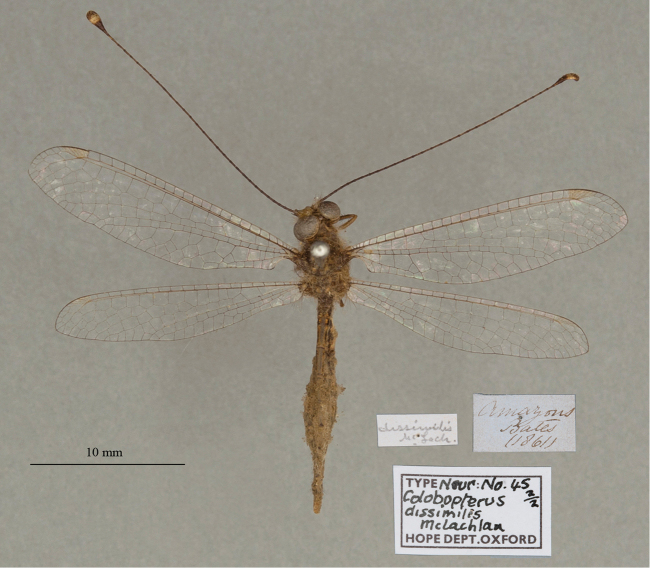
OUMNH paralectotype of *Colobopterusdissimilis* McLachlan, 1871 (NEUR0045-02, female).

**6. *dissimilis* McLachlan, 1871** (*Colobopterus*) (Lectotype, new designation; one paralectotype; Figs 12, 13)

**Original description.***J. Linn. Soc. Lond., Zoology, 1873a [1871], 11: 251*; “*Hab.* Amazons (*Bates*). This curious little species is remarkable for the dissimilarity of form in the sexes, as confirmed by the notes made in situ by Mr. Bates. I have seen only one ♂, which is in the Oxford Museum.”.

**Type series.** The description included information and measurements for both male and female specimens. Thus, we conclude that McLachlan’s type series consisted of the single male and at least one female.

Both van der Weele (1908: 136) and Penny (1981b: 632), respectively, mentioned “Typen” and “syntype male and female” in the OUMNH. Currently, two specimens (syntypes) are in the collection. One (NEUR0045-01, Fig. 12) has a “(Type)” label that probably is in McLachlan’s handwriting; it bears a “♂” notation and likely is the male that McLachlan noted in his description. Here, because of the differences between the sexes that McLachlan noted, we designate this male specimen as the lectotype (present designation). The other syntype (NEUR0045-02, Fig. 13), a female, now becomes a paralectotype.

In addition, there is one type (examined) in the NHMUK (NHMUK010212093). McLachlan’s label on this specimen indicates that it is a female; it also has an original “Brazil, Bates” label. We consider this specimen to be a paralectotype.

**Current name.***Ameropterusdissimilis* (McLachlan, 1871).

**Nomenclature.** The original genus name *Colobopterus* Rambur was identified as a junior homonym (Esben-Petersen 1922: 621). *Ameropterus* Esben-Petersen is the replacement name.

**Figure 14.**
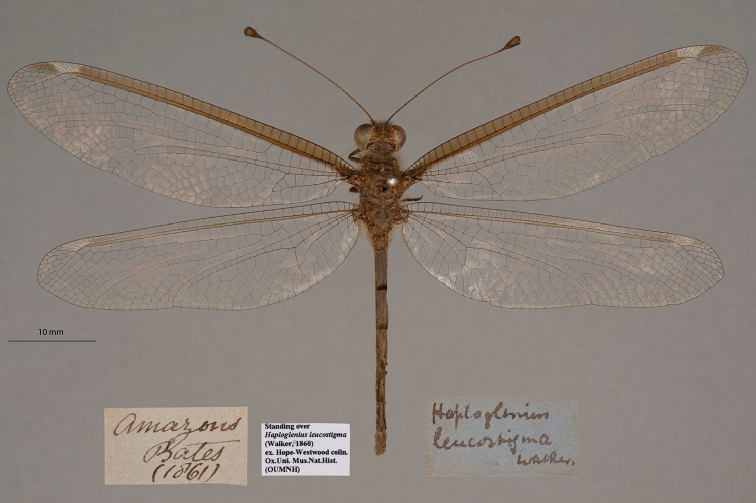
OUMNH syntype of *Ascalaphusleucostigma* Walker, 1860 (NEUR0078, sex undetermined).

**7. *leucostigma* Walker, 1860** (*Ascalaphus*) (One syntype; Fig. 14)

**Original description.***Trans. R. Ent. Soc. Lond., 10: 195*; “Amazon region.”. Sexes and number of specimens not specified.

**Type series.** The original description by Walker made no mention of the number or sexes of the specimens in the type series. About 10 years after the original description, McLachlan (1873a: 235) redescribed Walker’s species and specifically mentioned the collector (“Bates”), a female specimen, as well as two depositories (NHMUK and OUMNH). Thus, it is clear that there was more than one specimen in the type series and that the OUMNH housed at least one of them. Currently, there is one type, sex undetermined, in the OUMNH (NEUR0078, Fig. 14). It was standing over the name *leucostigma*. Its labels match the locality data in the original description and also the collector information that McLachlan had reported.

Penny (1981b: 613) referred to a specimen in the NHMUK as the “holotype”; however, he made no mention of selecting this specimen in favor of the other syntype(s) known to exist. Thus, under ICZN Article 74.5, this citation does not serve as a valid lectotype designation, and the two specimens in the NHMUK and the OUMNH remain as syntypes.

**Current name.***Ascalobyasmicrocerus* (Rambur, 1842).

**Nomenclature.**Hagen (1866: 384) synonymized *A.leucostigma* with *Haplogleniuscostatus* (Burmeister, 1839). Later McLachlan (1873a: 236) listed both “*H.leucostigma*, Walker” and “*H.costatus*, Burmeister” as valid species, without comment. Then, in 1908, also without comment, van der Weele (1908: 32) listed the two names *Ascalaphusleucostigma* and *Haplogleniusleucostigma* as synonyms of *Byasmicrocerus* Rambur; he also viewed *H.costatus* as a valid species. Penny (1981b: 613) confirmed the synonymy of *A.leucostigma* under *B.microcerus*, and he replaced the preoccupied generic name *Byas* with the new generic name *Ascalobyas* (Penny 1981a: 395).

**Figure 15.**
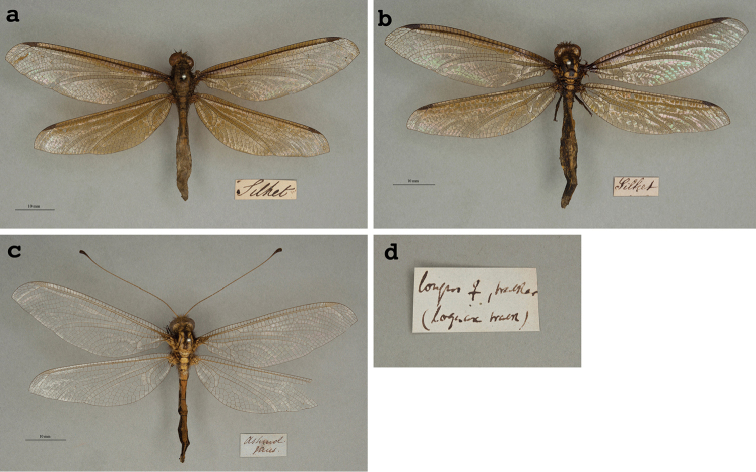
**a, b, c** Three historical OUMNH specimens of *Ascalaphuslongus* Walker, 1853, probably all female **d** the head label that rests above all three specimens.

**8. *longus* Walker, 1853** (*Ascalaphus*) (Three historical specimens; Fig. 15)

**Original description.***List of the specimens of neuropterous insects in the collection of the British Museum. Part II.--(Sialides--Nemopterides). British Museum, London. 1853: 435*; “Bengal.”. Two specimens indicated as: “a” and “b”.

**Type series.**Walker (1853: 432, 434, 435) described four very similar species of *Ascalaphus*: three from Bengal, and one without locality data. Types of all four species, including *A.longus*, are in the NHMUK, and the *A.longus* type carries the label (NHMUK010212097). Both McLachlan (1873a: 265) and van der Weele (1908: 230) examined the types of all four species, but lectotypes have not been designated. Apparently, numerous specimens were also in the de Sélys collection (van der Weele 1908: 230).

We also found three additional specimens (probably all female, unconfirmed) in the OUMNH standing over the name “*Acheronlongus*”. Two of these specimens bear labels reading “Silhet” (Fig. 15a, b), and the third specimen has no data other than a label that reads “Ashmole Mus” (Fig. 15c). The locality “Silhet” is in Bangladesh (formerly East Bengal). Thus, it is possible that these specimens were included among the type series that Walker used in his original description. However, this suggestion is unconfirmed, and at this time we are not identifying them as syntypes. [Note: Walker (1853: 432) used the term “Group *Acheron*, Lefèbvre” to refer to *Ascalaphustrux* Walker, one of the four species that is very closely related to *A.longus*. Later, the group name *Acheron* replaced the genus name for both species].

The large label under all three specimens in the OUMNH reads “longus ♀, Walker (loquax Walk)”; it probably was written by McLachlan (Fig. 15d). The evidence (the position of the head label and specimens in the collection, and the locality labels on the specimens) indicates that these specimens likely were examined by McLachlan prior to his discussion of Walker’s descriptions (see below).

**Current name.***Acheronlongus* (Walker, 1853).

**Nomenclature.**McLachlan (1873a: 265) examined the variation among the four species that Walker described, and he synonymized three of the species (*A.trux, A.loquax*, and *A.anticus*) under *A.longus*. He specifically indicated that *A.longus* was the senior synonym of the four. He also transferred *A.longus* to the genus *Acheron* Lefèbvre, 1842 and designated it as the type species of the genus. According to ICZN Article 24, McLachlan, as First Reviser, was justified in establishing precedence among the simultaneously published names. Thus, the name (*longus*) that he selected as the senior synonym has priority over the other three names.

Later, van der Weele (1908: 228) similarly recognized the four species names as synonymous, but he contradicted McLachlan’s earlier (1873a) work that already had established precedence among the names. Unfortunately, subsequent authors also appear to have ignored McLachlan’s early synonymies; as a result, the species with its various synonyms is erroneously listed in later systematic work and catalogs as *A.trux* (e.g., Fraser 1922: 516, Ghosh and Sen 1977: 321, Ghosh 1988: 175–179, Ghosh 2000: 105, Oswald 2018). Although the junior synonym has been used frequently, the reversal of precedence has not been exclusive (e.g., Needham 1909: 198), and, to our knowledge, it has not been in prevailing usage as defined by ICZN Article 23.9. Thus, the original revisionary work of McLachlan retains its priority, and *A.longus* (not *A.trux*) remains as the valid name.

**Figure 16.**
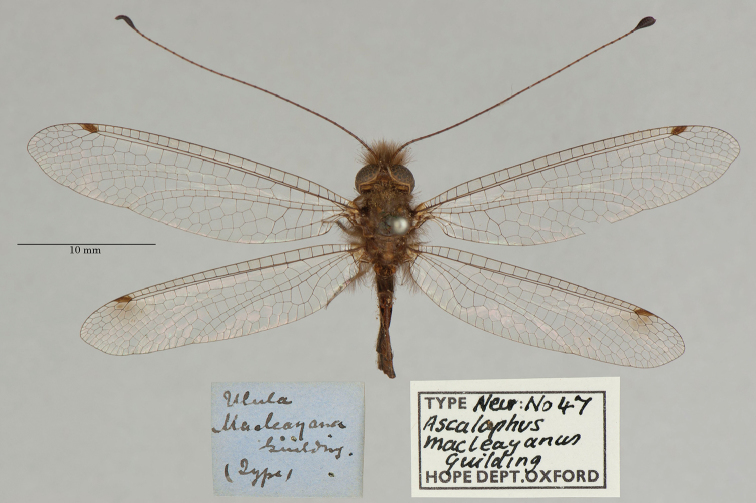
First of two OUMNH adult syntypes of *Ascalaphusmacleayanus* Guilding, 1823 (NEUR0047-01, sex undetermined).

**Figure 17.**
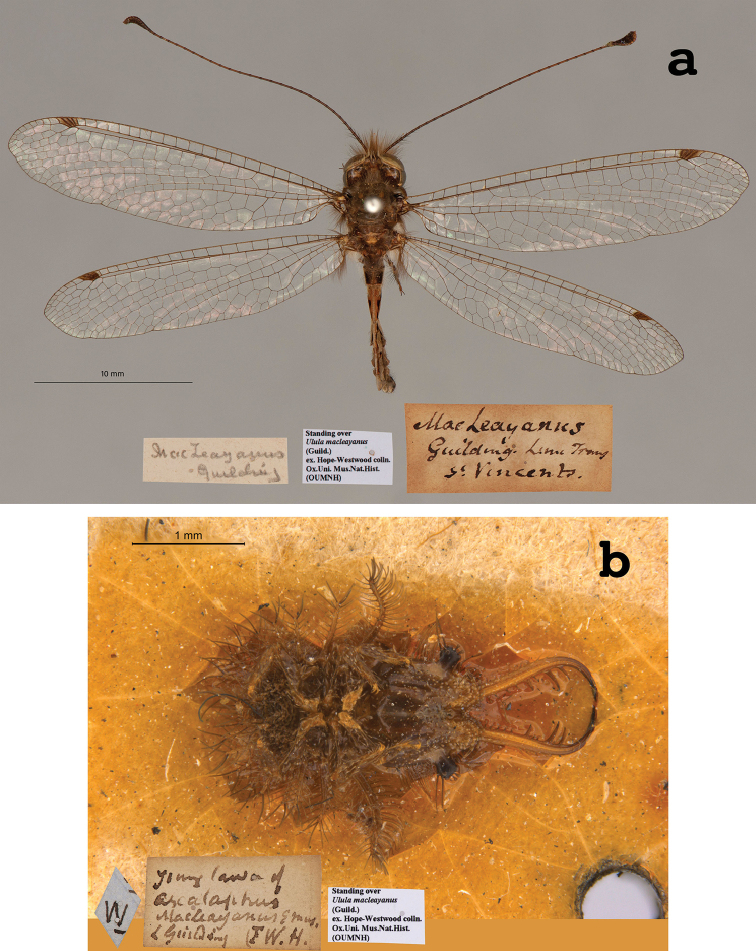
Second and third OUMNH syntypes of *Ascalaphusmacleayanus* Guilding, 1823 **a** adult (NEUR0047-02, sex undetermined) **b** larva (NEUR0047-03).

**9. *macleayanus* Guilding, 1823** (*Ascalaphus*) (Two adult syntypes and one larval syntype; Figs 16, 17)

**Original description.***Trans. Linn. Soc. Lond., 1825 [1823], 14: 140*; “… Ste Vincentii; … Mensibus Maio, Jan., Feb., occurrit. [Saint Vincent in the Lesser Antilles, Caribbean Region, flight during May, Jan., Feb.]”.

**Type series.** The description’s notations – “Varietas β. (an sexus alter?)”, and “*Ova* oblonga …”, “*Larva Pupa*que latent” – indicate that during the description Guilding had studied more than one adult specimen, as well as eggs and immatures. Later, McLachlan (1873a: 247) also reported that there were several specimens (including larvae) in the OUMNH. Subsequently, van der Weele (1908: 101–102) stated, in the plural, that “Die Typen, Larven, etc. sind im Oxford Museum.” In contrast, Penny (1981b: 646) and Penny et al. (1997: 42) indicated that no type was seen in the OUMNH, but that there was a “Holotype male” in the NHMUK.

We found three specimens in the OUMNH. One adult, with a museum type label (NEUR0047-01, Fig. 16), was in the type collection. The tip of its abdomen is missing or damaged, and its sex is unknown. Two additional specimens with appropriate label data were found near the type collection. One (NEUR0047-02, Fig. 17a) is an adult, probably of the same species as the primary type (sex undetermined). It does not correspond to the description of Var. β mentioned by Guilding. The other specimen is a larva (first instar, NEUR0047-03, Fig. 17b). We consider that both of these specimens are part of the type series.

Our reasons for including the larval specimen as part of the type series are several. First, Guilding (1825) provided a clear description of the egg and also included categories for larvae and pupae in his description. Second, the larval specimen in the collection is a newly hatched first instar that most likely emerged from an egg that Guilding obtained from one of his adult specimens. Thus, it was available at the time he prepared the description of the adult. Third, the term “latent” that Guilding used to describe the larva and/or pupa has several meanings, including (but not limited to) “hidden”, “concealed”, “lying”, “unknown”, “escaping notice”. Given this range of meanings, he may have used the term “latent” to refer to the larval habit of concealing itself. Fourth, the larval specimen carries a Westwood label and another old label clearly identifying it as *Ascalaphusmacleayanus*. Together, these clues indicate that in all probability the larval specimen was examined by Guilding at the time of the description and should be included as a syntype.

We are unaware of a lectotype designation for this species. At this point, the three specimens in the OUMNH are the only confirmed syntypes. However, there are multiple specimens of the species in the NHMUK collection that should be examined.

**Current name.***Ululodesmacleayanus* (Guilding, 1823).

**Nomenclature.** Originally assigned to *Ascalaphus*, the species was listed in *Suhpalacsa* by Hagen (1866: 385, as *Suphalasca*). Later, McLachlan (1873a: 247) moved it to the genus *Ulula*, and subsequently Banks (1907: 32) synonymized it under *Ululodeshyalinus* (Latreille, 1817, as *hyalina*). However, the species name with the current combination was reinstated as valid by van der Weele (1908: 101). Six subspecific names have been proposed for this species (van der Weele 1908: 101); apparently not all are valid (see Penny 1981b: 648).

**Figure 18.**
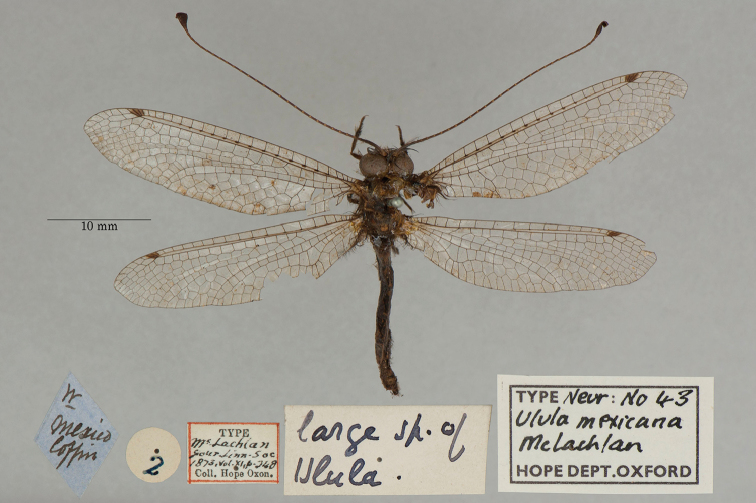
OUMNH syntype of *Ululamexicana* McLachlan, 1871 (NEUR0043, probably male).

**10. *mexicana* McLachlan, 1871** (*Ulula*) (One syntype; Fig. 18)

**Original description.***J. Linn. Soc. Lond., Zoology, 1873a [1871], 11: 248*; “*Hab.* Mexico. In De Sélys’s collection and in the Oxford Museum.”. Sexes and number of specimens not specified.

**Type series.** Because the statement in the original description indicates two depositories, clearly the type series contained more than one specimen. We found one syntype in the OUMNH (NEUR0043, Fig. 18). The male hindwing of this species typically is unmarked (van der Weele 1908: 116, Figs 77, 78), whereas the female hindwing bears a heavy mark. We have not examined the abdomen of the type, but based on the lack of a wing mark, we expect that the specimen is male. The notes by van der Weele (1908: 116; also see Oswald 2018) provide considerable information on the type specimens in the de Sélys collection.

**Current name.***Ululodesmexicanus* (McLachlan, 1871).

**Nomenclature.** The combination *Ululodesmexicanus* was first used by van der Weele (1908: 116, as “*U.mexicana*”). The Greek suffix “-odes” of the genus name “*Ululodes*” is considered masculine (ICZN Article 30.1.4.4), and therefore the species name was altered to conform.

**Figure 19.**
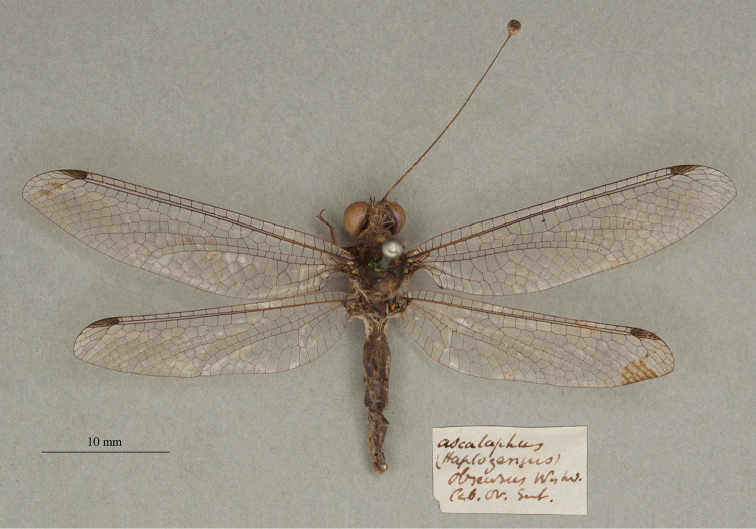
OUMNH syntype of Ascalaphus (Haploglenius) obscurus Westwood, 1847 (NEUR0073, sex undetermined).

**11. *obscurus* Westwood, 1847** [Ascalaphus (Haploglenius)] (One syntype; Fig. 19)

**Original description.***The Cabinet of Oriental Entomology; being a selection of the rarer and more beautiful species of insects, natives of India and the adjacent islands. The greater portion of which are now, for the first time, described and figured. Smith, London, 1848 [1847]: 69*; “Inhabits the East Indies. Col. Hearsey.”. Sexes and number of specimens not specified.

**Type series.** The type of this species, like those of some other species that were in the Cabinet of Oriental Entomology, at one time was considered lost (McLachlan 1891: 513, van der Weele 1908: 68, others listed in Oswald 2018). However, specimens eventually turned up in various collections. Thus, we were not surprised when we found a type for *A.obscurus* in the OUMNH; we consider it to be a syntype, sex undetermined (NEUR0073, Fig. 19). Although its label carries no locality data, it does state the species name and “Cab. Or. Ent.” as per the description, and the handwriting is similar to that of other Westwood types (e.g., *Ascalaphusdentifer* Westwood). Moreover, the specimen is surrounded by, and appears to have been prepared in a manner typical of, other specimens collected at the time in the East Indies by Col. Hearsey.

**Current name.***Stylascalaphusobscurus* (Westwood, 1847).

**Nomenclature.** Previous combinations include *Haploglenius* (?) *obscurus* (Westwood, 1847) by Hagen (1866: 386), *Idricerus? obscurus* Mac Lachlan [sic] by van der Weele (1908: 68), and *Stylonotusobscurus* (Westwood, 1847) by Needham (1909: 199). The current combination is attributed to Sziráki (1998: 65), who proposed *Stylascalaphus* as the replacement name for *Stylonotus* Needham, a junior homonym.

**Figure 20.**
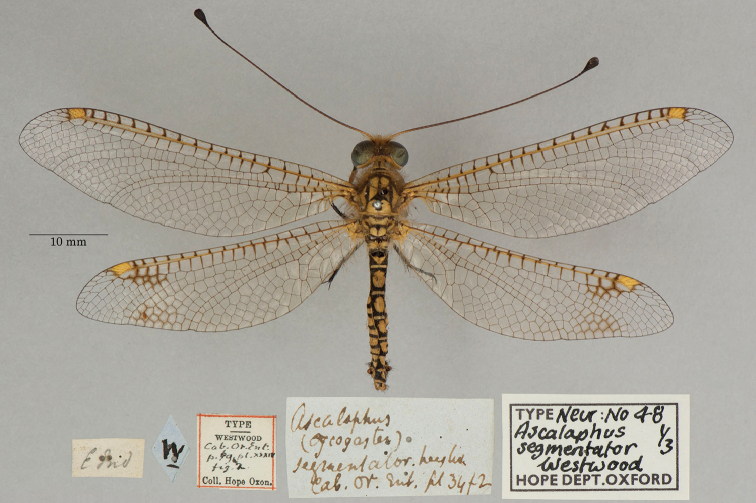
First of three OUMNH syntypes for Ascalaphus (Ogcogaster) segmentator Westwood, 1847 (NEUR0048-01, probably male).

**Figure 21.**
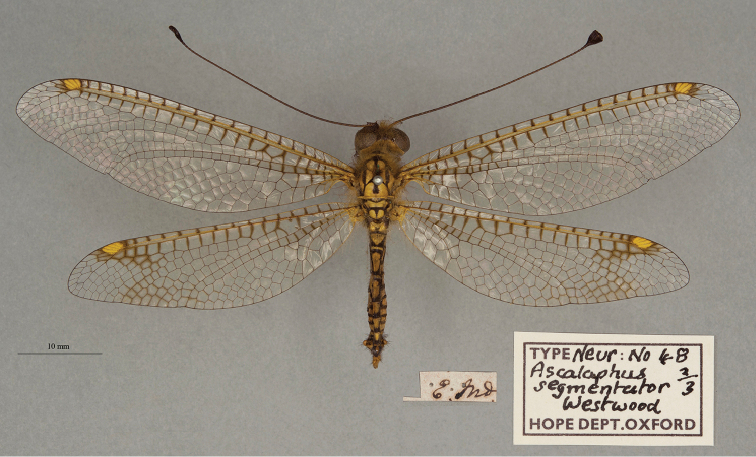
Second of three OUMNH syntypes for Ascalaphus (Ogcogaster) segmentator Westwood, 1847 (NEUR0048-02, probably male).

**Figure 22.**
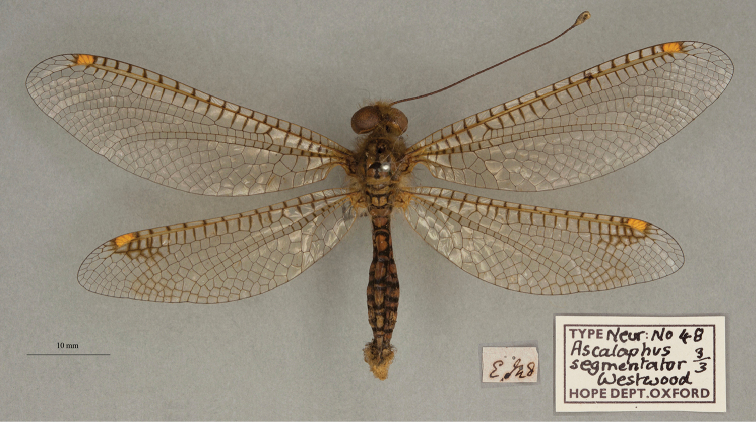
Third of three OUMNH syntypes for Ascalaphus (Ogcogaster) segmentator Westwood, 1847 (NEUR0048-03, probably female).

**12. *segmentator* Westwood, 1847** [Ascalaphus (Ogcogaster)] (Three syntypes; Figs 20, 21, 22)

**Original description.***The Cabinet of Oriental Entomology; being a selection of the rarer and more beautiful species of insects, natives of India and the adjacent islands. The greater portion of which are now, for the first time, described and figured. Smith, London, 1848 [1847]: 69, fig. 2.* “Inhabits the East Indies. In the Collection of W. W. Saunders, Esq., and my own.”. Sexes and number of specimens not specified.

**Type series.** Westwood’s reference to two depositories indicates that he had more than one specimen in his type series. Three syntypes are in the OUMNH; the first two appear to be males (NEUR0048-01, -02; Figs 20, 21), the third a female (NEUR0048-03, Fig. 22) (sexes unconfirmed). The first specimen (NEUR0048-01) carries four labels, probably all in Westwood’s handwriting, that indicate the locality data, species name, Westwood’s collection, and the specimen’s association with the Cabinet of Oriental Entomology.

In addition to the three OUMNH types above, Walker (1853: 421) reported six specimens collected in the East Indies that are housed in the NHMUK (from the collections of F. C. Dale, Mr. Sowerby, and Mr. Stevens). We found numerous *O.segmentator* specimens in the NHMUK (at least seven males and three females) that could be syntypes, but none are identified as such. It is clear that the history of these specimens should be explored. As far as we know, a lectotype has not been designated.

**Current name.***Ogcogastersegmentator* (Westwood, 1847).

**Nomenclature.** Westwood described the species within the subspecies Ascalaphus (Ogcogaster). Later Hagen (1866: 386) referred to it as *Ogcogastersegmentator* Westwood. Hagen’s combination was cited subsequently by McLachlan (1873a: 265) and Sziráki (1998: 64).

**Figure 23.**
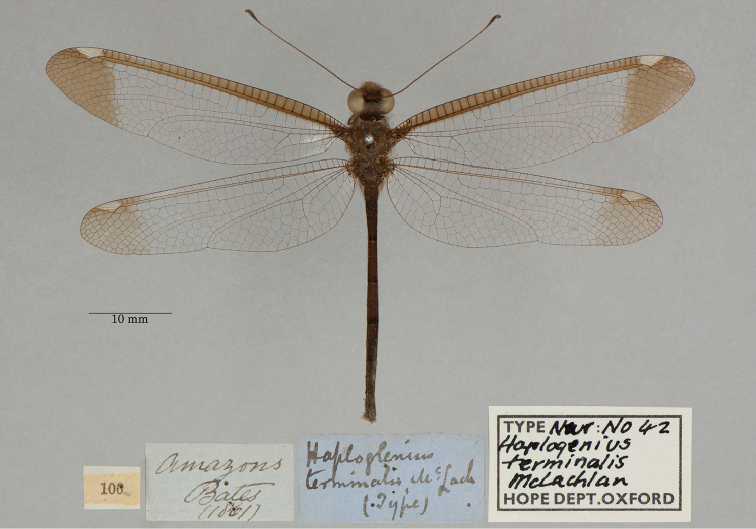
OUMNH syntype *of Haploglenius terminalis* McLachlan, 1871 (NEUR0042, probably male).

**13. *terminalis* McLachlan, 1871** (*Haploglenius*) (One syntype; Fig. 23)

**Original description.***J. Linn. Soc. Lond., Zoology, 1873a [1871], 11: 235*; “*Hab.* Tapajos. (*Bates*). In the British and Oxford Museums.”. Sexes and number of specimens not specified. [“Tapajós” refers to a tributary that joins the Amazon River near Santarém, one of Bates’ primary collecting areas].

**Type series.**Penny (1981b: 610) reported seeing two syntype males in the NHMUK; currently those specimens are present (NHMUK010212101, NHMUK010212102). Because McLachlan (1873a: 235) reported types in two depositories, the type series must have contained at least one more specimen.

We found a syntype, probably male (unconfirmed), in the OUMNH (NEUR0042, Fig. 23). To our knowledge, no lectotype has been designated; thus the three specimens (NHMUK and OUMNH) remain as syntypes.

**Current name.***Ascalobyasalbistigma* (Walker, 1853).

**Nomenclature.** The species was synonymized with *Byasalbistigma* (Walker, 1853) by van der Weele (1908: 33). Subsequently, Penny (1981a: 395) recognized the generic name *Byas* Rambur as a junior homonym and proposed *Ascalobyas* as the generic replacement name. The current name was cited by Penny (1981a: 609) and Penny et al. (1997: 41).

**Figure 24.**
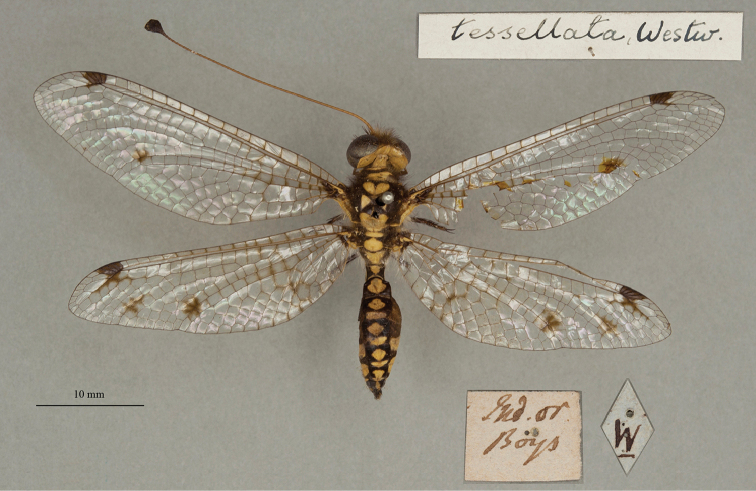
First of three OUMNH syntypes (or possible syntypes) for Ascalaphus (Ogcogaster) tessellatus Westwood, 1847 (NEUR0074-01, probably female), with head label (upper right).

**Figure 25.**
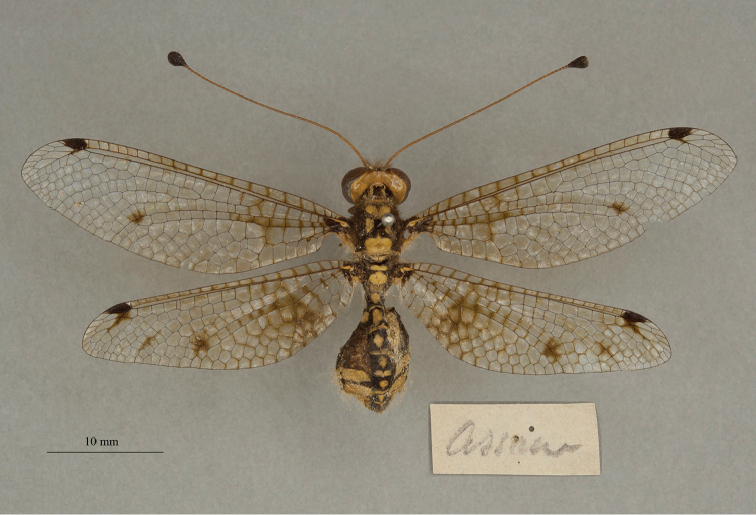
Second of three OUMNH syntypes (or possible syntypes) for Ascalaphus (Ogcogaster) tessellatus Westwood, 1847 (NEUR0074-02, probably female).

**Figure 26.**
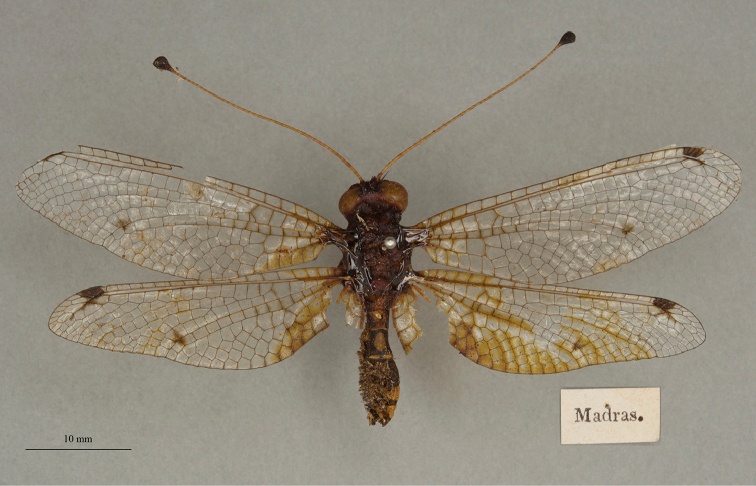
Third of three OUMNH syntypes (or possible syntypes) for Ascalaphus (Ogcogaster) tessellatus Westwood, 1847 (NEUR0074-03, probably female).

**14. *tessellatus* Westwood 1847** [Ascalaphus (Ogcogaster)] (Three syntypes or possible syntypes; Figs 24, 25, 26)

**Original description.***The Cabinet of Oriental Entomology; being a selection of the rarer and more beautiful species of insects, natives of India and the adjacent islands. The greater portion of which are now, for the first time, described and figured. Smith, London, 1848 [1847]: 69, fig. 1.* “Inhabits the East Indies. In the Collection of W. W. Saunders, Esq.”. Sexes and number of specimens not specified.

**Type series.** Apparently lacking information on Westwood’s types in the OUMNH, van der Weele (1908: 253–254, as *Ogcogastertessellata*) stated: “Die Typen [plural] sind im Londoner Museum.”, and “…is nur in ♀♀ bekannt, … [… is only known from ♀♀, …]”. Subsequently, a single female type was reported in the NHMUK by Fraser (1922: 518, as *Ogcogastertessalata*).

We also found three specimens in the OUMNH uncatalogued type collection. They were curated under a modern, handwritten label with the name *tessellata* (Fig. 24, upper right corner), and all appear to be females (sexes unconfirmed). One (NEUR0074-01, Fig. 24) bears two labels, both in Westwood’s handwriting: a locality label reading “Ind. or. / Boys”, and a typical Westwood label – a diamond with the letter “W” in the center. Given its labels, we believe that it probably is a syntype. Although the other two specimens lack Westwood’s diamond-shaped label, they also might be from the type series. They each bear a single label: one reading “Assam” (NEUR0074-02, Fig. 25), and the other reading “Madras” (NEUR0074-03, Fig. 26). At the time the species was described, both localities were considered to be within the East Indies.

Among the specimens of *A.tessellatus* in the NHMUK, one is labeled as a type; it carries the museum number (NHMUK010288521) and has handwritten identification labels. Also, two specimens are present without type labels, but they have labels that are very similar to those on the specimens with type labels. One (NHMUK010212100) is a plump female; the other (NHMUK010212099) is more slender and could be a male. We suggest that the history and type status of these specimens be explored before a lectotype is designated for this species.

Note: The Lacewing Digital Library (Oswald 2018) states that the type is a “holotype, by implicit monotypy”; it cites the article by Fraser (1922) in which the author reports: “Type in Brit. Mus., a female”. We do not agree that Fraser necessarily assumed that the specimen he examined in the NHMUK was the only type for this species. Indeed, if he had, he would have been in conflict with published information available at the time, e.g., van der Weele (1908: 253–254). Moreover, Fraser did not specify which of the possible type specimens in the NHMUK he considered to be the primary type; there are at least three possibilites (see above). Thus, under ICZN Article 74.5, Fraser’s reference to a type does not constitute a valid lectotype designation, and the three specimens in the NHMUK and the three in the OUMNH remain as syntypes or possible syntypes.

**Current name.***Ogcogastertessellata* Westwood, 1847.

**Nomenclature.** The original name Ascalaphus (Ogcogaster) tessellatus remains as an unreplaced junior homonym of *Ascalaphustessellatus* described by Lichtenstein (1796: 192). The homonym represents a valid biological entity that is treated as such until replacement. See Oswald (2018) for details.

Originally assigned to Ascalaphus (Ogcogaster), the current combination, *Ogcogastertessellata* Westwood, was first used by Hagen (1866: 387, as *tessellatus*).

**Note.**Fraser (1922: 518), Sziráki (1998: 64), and Ghosh (2000: 107) either wrote or quoted “*tessellatus*” or “*tessellata*” as “*tessalatus*”, “*tessalata*”, “*tesselatus*”, “*tesselata*”, all of which are incorrect subsequent spellings.

#### Chrysopidae (Green lacewings)

With approximately 1200 species, the Chrysopidae is the second largest neuropteran family. It is distributed worldwide; specimens are frequently encountered in nature; and some species are used in the biological control of agricultural and horticultural arthropod pests. The classification of the chrysopid species inhabiting some parts of the world (e.g., Europe, South Africa, Australia, Japan, North America - north of Mexico) is reasonably well developed. However, the faunae from other regions are in need of considerable systematic work. At this time, the revision of the Chrysopidae by Brooks and Barnard (1990) provides the only comprehensive (worldwide) systematic coverage of the genera in the family, and it is now almost 30 years old. Moreover, modern revisions are greatly needed for most genera.

Despite the large size and wide distribution of the family, the OUMNH houses only five chrysopid type specimens, all of which are name bearing. Most are from the Old World. Four were described by J. P. Rambur in 1842, and one by L. Navás in 1913. Taxonomic issues associated with two of the species (*Hemerobiusbrevicollis* Rambur and *H.conformis* Rambur) were treated earlier (Tauber 2017).

**Figure 27.**
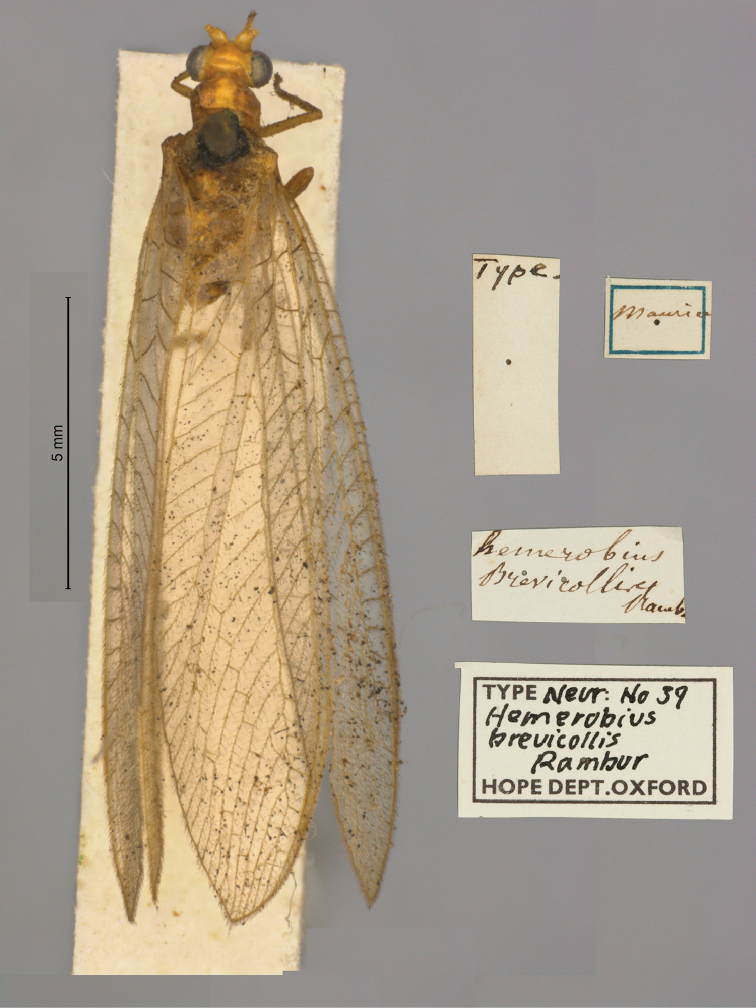
Lectotype of *Hemerobiusbrevicollis* Rambur, 1842 (NEUR0039, female).

**1. *brevicollis* Rambur, 1842** (*Hemerobius*) (Lectotype; Fig. 27)

**Original description.***Libr. encycl. Roret, 1842: 427*; “Rapporté de l’ile de France par M. Marchal.”. Sexes and number of specimens not specified.

**Type series.** The female specimen in the OUMNH is the lectotype (NEUR0039, Fig. 27); additional images and taxonomic notes are provided by Tauber (2017: 81). The specimen is in reasonably good condition, except that most of its abdomen is missing; basal pieces of the abdomen are in a microvial with glycerol.

**Current name.***Chrysoperlabrevicollis* (Rambur, 1842).

**Nomenclature.** This species was previously known as *Chrysopabrevicollis* (Rambur) and erroneously listed as *Chrysoperlapudica* (Navás). See Brooks (1994: 162) and Tauber (2017: 83) for taxonomic history and status.

**Figure 28.**
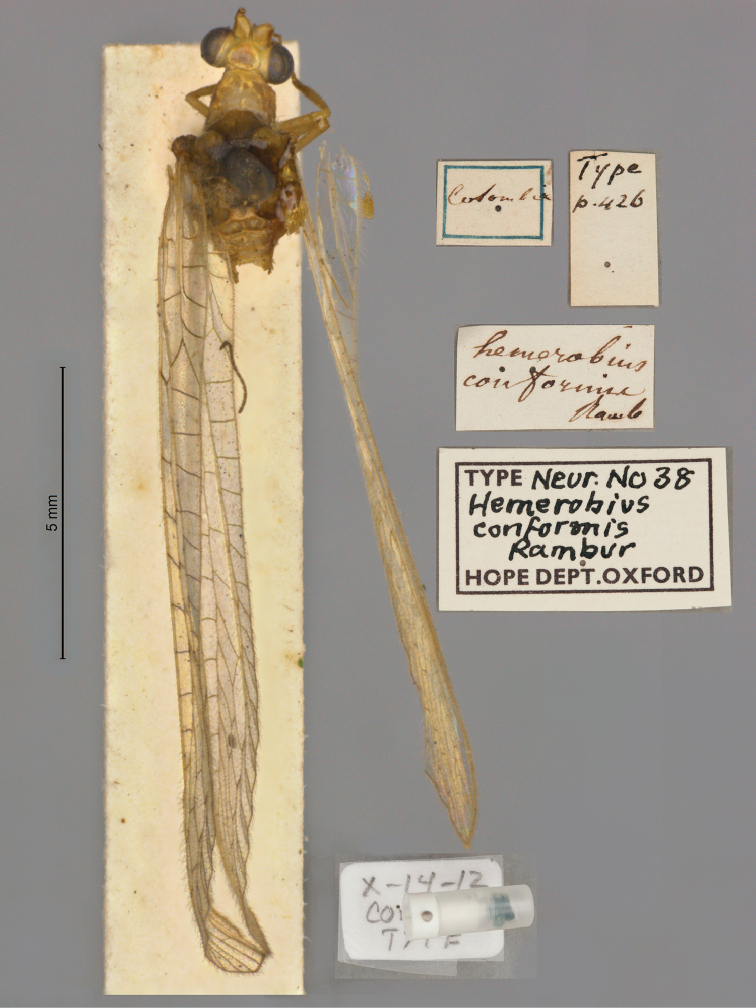
Lectotype of *Hemerobiusconformis* Rambur, 1842 (NEUR0038, female).

**2. *conformis* Rambur, 1842** (*Hemerobius*) (Lectotype; Fig. 28)

**Original description.***Libr. encycl. Roret, 1842: 426–7*; “De la Colombia; communiqué par M. Marchal.”. Sexes and number of specimens not specified.

**Type series.** A single type (female, NEUR0038, Fig. 28) exists in the OUMNH. Tauber (2017: 83) designated it as the lectotype and provided a redescription with images. The specimen is in good condition; the abdomen is dissected and held in a microvial with glycerol.

**Current name.***Ceraeochrysaconformis* (Rambur, 1842).

**Nomenclature.** Previously known as *Chrysopaconformis* (Rambur), the species was transferred to the large genus *Ceraeochrysa* (Tauber 2017: 83). Given that it is now the oldest in the genus, the species is not a junior synonym.

**Figure 29.**
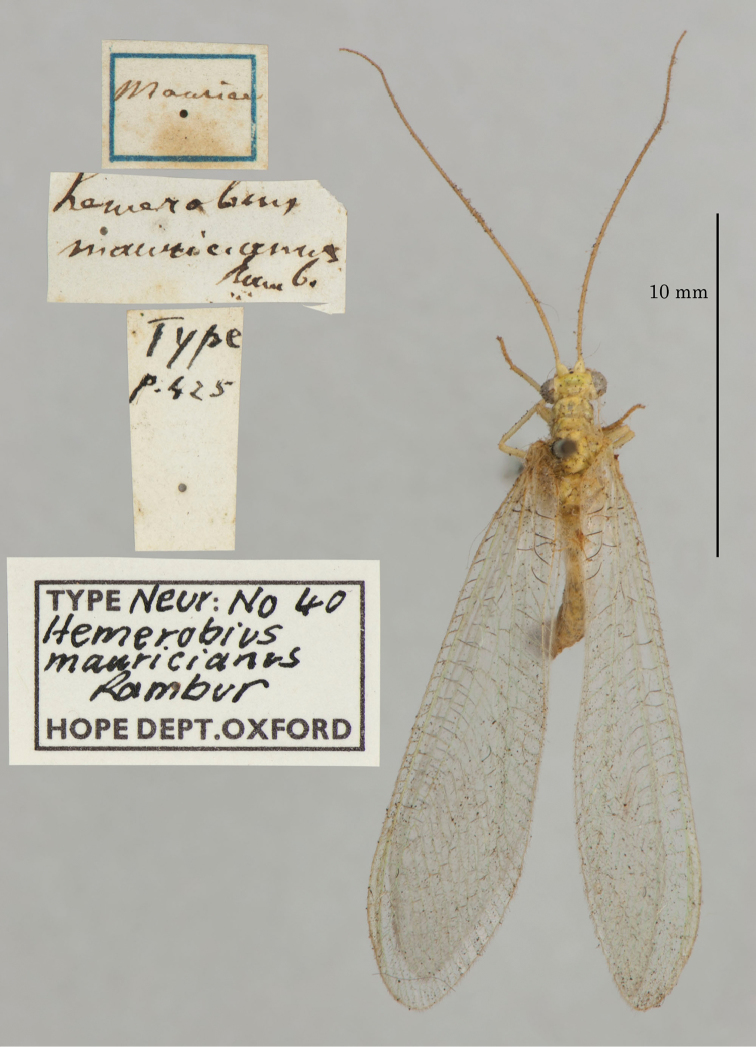
Holotype of *Hemerobiusmauricianus* Rambur, 1842 (NEUR0040, sex undetermined).

**3. *mauricianus* Rambur, 1842** (*Hemerobius*) (Holotype; Fig. 29)

**Original description.***Libr. encycl. Roret, 1842: 425–6*; “Habite Maurice. M. Marchal m’a communiqué un individu qu’il a pris lui-même dans cette ile. [Locality Mauritius. Mr. Marchal sent an individual to me that he collected on this island himself.]”.

**Type series.** The original description explicitly mentions one specimen; thus the OUMNH type, sex unknown, is the holotype (by explicit monotypy) (NEUR0040, Fig. 29). It looks very old but is in good condition.

**Current name.***Chrysopapallens* (Rambur, 1838).

**Nomenclature.***Hemerobiusmauricianus* was synonymized with *Chrysopaseptempunctata* Wesmael by Schneider (1851: 101), and later listed as such by Killington (1936–37: 194), Aspöck et al. (1980: 254), and others. Soon after Schneider’s publication, *C.septempunctata* was identified as a senior synonym of *Chrysopapallens* (Rambur, 1838) (see Hagen 1866: 395); later the synonymy was reversed (Leraut 1980: 242). Now, both *C.septempunctata* and *H.mauricianus* are regarded as synonyms of *C.pallens* and are cited as such by Brooks and Barnard (1990: 270), Aspöck et al. (2001: 91), and others. However, the nomenclature of these and other closely related species is not yet settled. [Much of the above information was generously provided by R. A. Pantaleoni].

**Figure 30.**
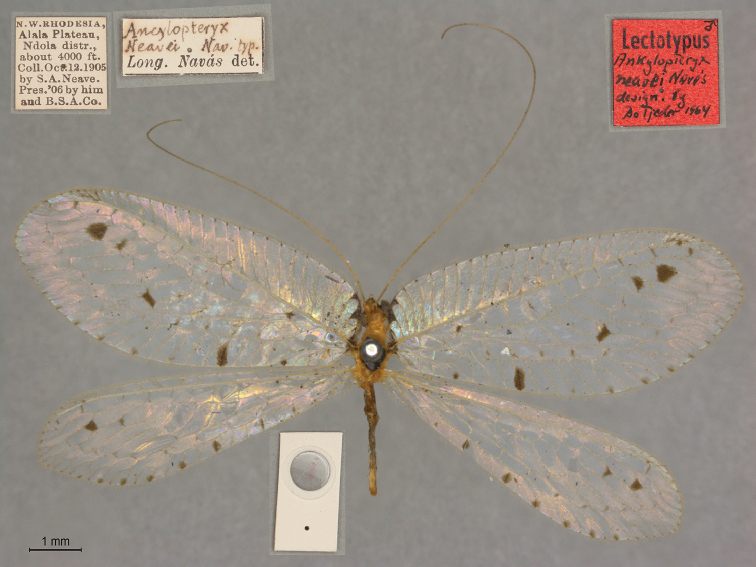
Lectotype of *Ankylopteryxneavei* Navás, 1913 (NEUR0075, male).

**4. *neavei* Navás, 1913** (*Ancyclopteryx*, a subsequent spelling of *Ankylopteryx*) (Lectotype; Fig. 30)

**Original description.***Ann. Soc. sci. Bruxelles, 1913b: 37 (pt. 1): 93*; “Rhodesia, Nord-Ouest, Plateau Alala, Ndola (environ 400 ft.), 11 oct. 1905, S. A. Neave coll. (Mus. d’Oxford).”. Sexes and number of specimens not specified.

**Type series.**Tjeder (1966: 509) designated the sole specimen, a male, in the OUMNH as the lectotype (NEUR0075, Fig. 30). The tip of the abdomen is cleared, stained, and mounted on a disc that is attached to the pin. Tjeder (1966: 509) also noted that the elevation and date reported in the original description differ from those on the insect labels (Oct. 12 and 4,000 ft. on the label versus Oct. 11 and 400 ft. in the original description). He attributed the discrepancies to a possible printer’s error.

**Current name.***Parankylopteryxpolysticta* (Navás, 1910).

**Nomenclature.** The original description assigned the species to the genus *Ankylopteryx* (as “*Ancyclopteryx*”, a subsequent incorrect spelling). It later was included in the new subgenus Ankylopteryx (Parankylopteryx) by Tjeder (1966: 508) and synonymized with *Parankylopteryxpolysticta* (Navás, 1910) by Hölzel and Ohm (1991: 59).

**Figure 31.**
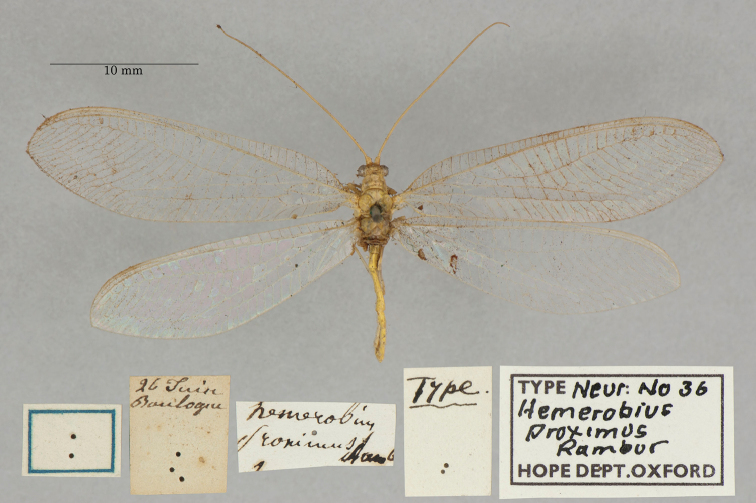
OUMNH syntype *of Hemerobiusproximus* Rambur, 1842 (NEUR0036, sex undetermined).

**5. *proximus* Rambur, 1842** (*Hemerobius*) (One syntype; Fig. 31)

**Original description.***Libr. encycl. Roret, 1842: 425*; “Se trouve dans les bois moins communément que le *Perla*. [Found in the woods less commonly than *perla*.]”. The description provides neither locality data nor any information about the number or sexes of the types that Rambur had.

**Type series.** One type (unknown sex), with label reading “Boulogne” [probably Boulogne-sur-Mer, in northern France], is in the OUMNH (NEUR0036, Fig. 31). Although we could find no published references to other type specimens for this species, at this point we consider the OUMNH specimen to be a syntype.

**Current name**: *Ninetavittata* (Wesmael, 1841).

**Nomenclature.***Hemerobiusproximus* was synonymized with *Chrysopavittata* Wesmael by Schneider (1851: 65). Later, Navas (1912b: 99) moved *C.vittata* into his new genus *Nineta* Navás without mention of *H.proximus*. Subsequently, he and others (Navás 1915a: 87, Aspöck et al. 1980: 240) listed the synonymy.

#### Coniopterygidae (Dusty wings)

The Coniopterygidae is a medium-sized family of small-bodied, cryptic insects (2–5 mm wing length). The wings and bodies of adults generally have a covering of light gray to white (“dusty”) wax that is produced by hypodermal glands. Larvae are predaceous on small arthropods, such as homopterans and mites; some species are considered important as natural biological control agents. The revision of the family Coniopterygidae by Meinander (1972) remains a gold standard for neuropteran systematics. It contains information on all species known at the time of publication, their synonymies, the location of type(s), descriptions, drawings, and keys.

Only 18 coniopterygid species were described before 1900. Three of these were described by McLachlan, and the OUMNH contains the holotype of one, a species from Australia. Types of the other two McLachlan species are in the NHMUK: (a) *Coniopteryxpulchella* McLachlan, 1882: 173, lectotype and paralectotype (Meinander 1972: 340), and (b) *Coniopteryxhaematica* McLachlan, 1868a: 193, lectotype (Meinander 1972: 270).

**Figure 32.**
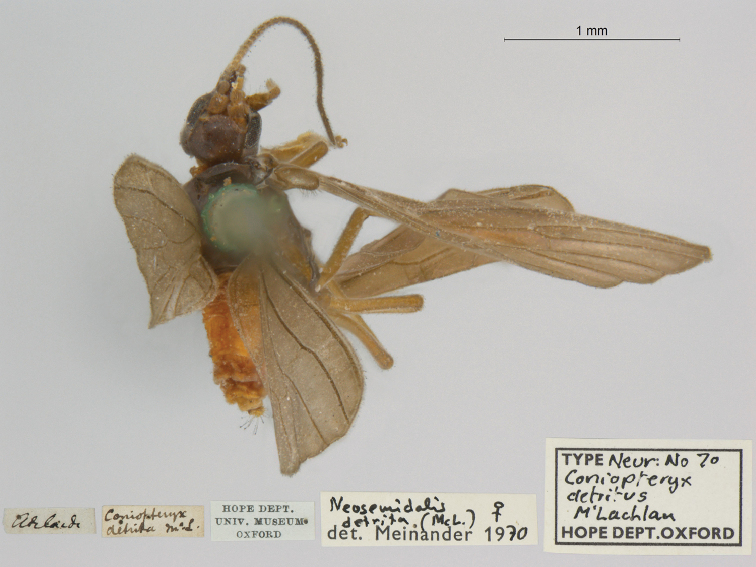
Holotype of *Coniopteryxdetrita* McLachlan, 1867 (NEUR0070, female).

**1. *detrita* McLachlan, 1867** (*Coniopteryx*) (Holotype; Fig. 32)

**Original description.***Entomol. Monthly Mag., 4: 151*; “Habitat ad Adelaide in Australia meridionali. In collect. Mus Oxon. One example in good condition.”.

**Type series.** In the OUMNH, there is one specimen, a female, with data matching the original description. It is the holotype by original designation (NEUR0070, Fig. 32).

**Current name.**Neosemidalis (Neosemidalis) detrita (McLachlan, 1867).

**Nomenclature.** The current combination was first used by Enderlein (1930: 107). The species was also previously known as *Parasemidalisdetrita* (McLachlan). For additional information, see Meinander (1972: 174) and New (1996: 7).

#### Hemerobiidae (Brown lacewings)

Hemerobiidae is the third largest family in the order Neuroptera; it contains approximately 550 species. Adults are generally small to medium sized and inconspicuous; as their name implies, the wings of most species are brown. However, wing shape and size vary markedly among groups, as do their habitats and plant/host associations. In general, larvae feed on small, soft-bodied arthropods. A comprehensive taxonomic study by Oswald (1993) provides comparative morphological (adult) data and a phylogenetic classification for hemerobiid genera worldwide. In contrast, the quality of information at the species level varies regionally, with most work centered in Europe. Overall, the group is in need of much taxonomic work.

Although brown lacewings are numerous and diverse in Europe, the family is represented by only one type in the OUMNH. The specimen is the holotype of a species that was collected in France and described by Killington.

**Figure 33.**
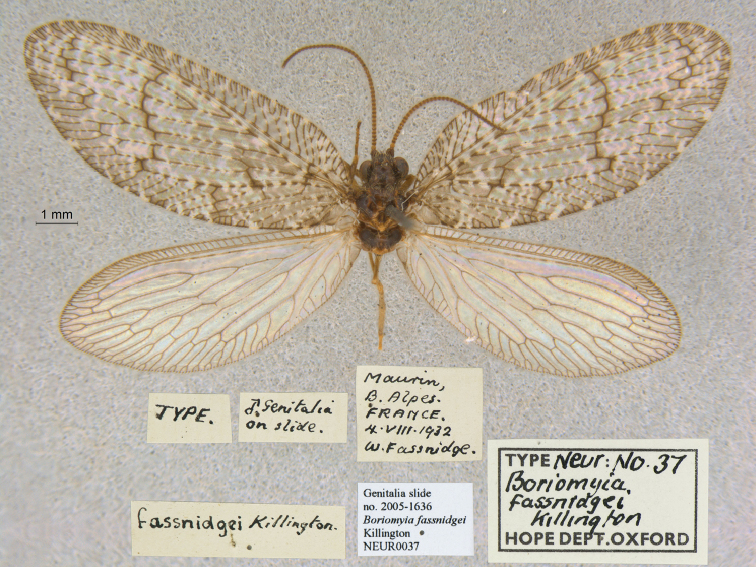
Holotype of *Boriomyiafassnidgei* Killington, 1933 (NEUR0037, male).

1. ***fassnidgei*** Killington, 1933 (*Boriomyia*) (Holotype; Fig. 33)

**Original description.***Entomol. Monthly Mag., 69: 57, figs 1–4.* “FRANCE, Maurin (5,000 ft.), Basses-Alpes. Taken at light, 4^th^ Aug., 1932, by Mr. Wm. Fassnidge. … ♂ in my collection; genitalia preserved as a microscope preparation.”.

**Type series.** Killington’s specimen, the holotype by original designation, is in the OUMNH (NEUR0037, Fig. 33). Both the specimen and Killington’s slide with the mounted male genitalia are as stated in the original description. The database of the NHMUK also lists a type (NHMUK010110716); however, the collection data (locality, date) and the mount of the specimen are not consistent with the original description. Thus, the identification of this specimen and its report as the holotype in the online Lacewing Digital Library (Oswald 2018) are in error.

**Current name.***Wesmaeliusfassnidgei* (Killington, 1933).

**Nomenclature.** The current combination was first used by Kis et al. (1970: 179). The species was also known as *Kimminsiafassnidgei* [combination by Aspöck (1963: 53)]. See Aspöck et al. (1980: 200; 2001: 139) for more information.

#### Mantispidae (Mantidflies)

Among the Neuroptera, Mantispidae is a moderately large family, with approximately 400 species. It occurs throughout the warm regions of the world, with particularly rich faunae in the tropical regions of Australasia, Africa, and Central and South America. Adults are easily recognized by their raptorial forelegs that resemble those of preying mantids (Mantodea: Mantidae). Larvae are hypermetamorphic; first instars are campodeiform and probably do not feed. The later two instars are grub-like and are known to feed on concealed prey. Some have been reported to prey on encased spider eggs, and others consume aculeate hymenopteran larvae in their nests, in a manner similar to parasitoidism.

The Hope Collection contains a rich assemblage of types and historically important specimens of mantispids. Types of 29 species are reported to be in the collection, and we found type specimens for all of them, including 26 species with primary types (holotype, syntype, or lectotype). Altogether, a total of 49 mantispid type specimens reside in the collection, with 14 from the Old World (Middle East, southern and western Africa, India, Australia, and Oceania) and 15 from the New World (mostly Brazil with H. W. Bates as collector, also Colombia and Venezuela). The authors of the descriptions include Westwood, Navás, Hagen, McLachlan, and Rambur; the range in years of publication is from 1842 to 1914.

The mantispid types presented some of the most challenging nomenclatural issues, probably because they tended to receive more attention from earlier entomologists than did the types in other families. Previous treatment was not always consistent among species, nor with the current ICZN Rules. For quite a few mantispid species, holotype determinations were made (and published) with no or very limited evidence concerning the possible existence of other syntypes. In general, these determinations were accepted into the literature and common usage. In contrast, syntypes of other species that were not studied in the past now tend to receive more rigorous treatment, and holotype assignations are rarely applied. Here, we made a concerted effort to treat all the species similarly, based on their history and a uniform application of the ICZN Rules. After our study, of the 26 species of mantispids with primary types in the OUMNH, twelve are represented by holotypes, nine by lectotypes, and, in stark contrast with the Ascalaphidae (see above), only five by syntypes.

A recent catalog of the world’s Mantispidae (Ohl 2004) provides a useful record of known mantispid names, along with their synonymies, the locations of their types, and other pertinent information. It does not provide references for most synonymies or name changes; to obtain that information, we conducted our own search of the literature, with the help of the online Lacewing Digital Library (Oswald 2018).

**Figure 34.**
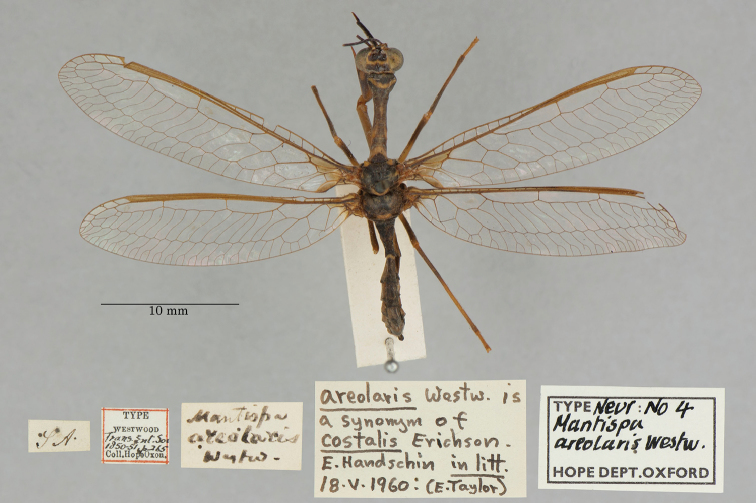
OUMNH syntype of *Mantispaareolaris* Westwood, 1852 (NEUR0004, sex undetermined).

**1. *areolaris* Westwood, 1852** (*Mantispa*) (Syntype; Fig. 34)

**Original description.***Trans. R. Entomol. Soc. Lond., 6 [1]: 265, tab. 18. fig. 3*; “Habitat in Brasilia. Mus. Hope.”. Sexes and number of specimens not specified.

**Type series.**Ohl (2004: 170) reported seeing a holotype (or syntype), sex unknown, in the OUMNH. Oswald (2018) classified the specimen similarly. We found a single type in the collection (NEUR0004, Fig. 34); its sex is unconfirmed. Given that there is no information on the original number of specimens in the type series, we too consider the specimen to be a syntype.

**Current name.***Entanoneuracostalis* (Erichson, 1839).

**Nomenclature.** The synonymy of *M.areolaris* under *E.costalis* was proposed by Handschin (1960b: 534); listed as such by Ohl (2004: 170). The species was previously known as *Entanoneuraareolaris* [combination by Enderlein (1910: 359)] and Mantispa (Entanoneura) areolaris [combination by Williner and Kormilev (1958: 8)].

**Figure 35.**
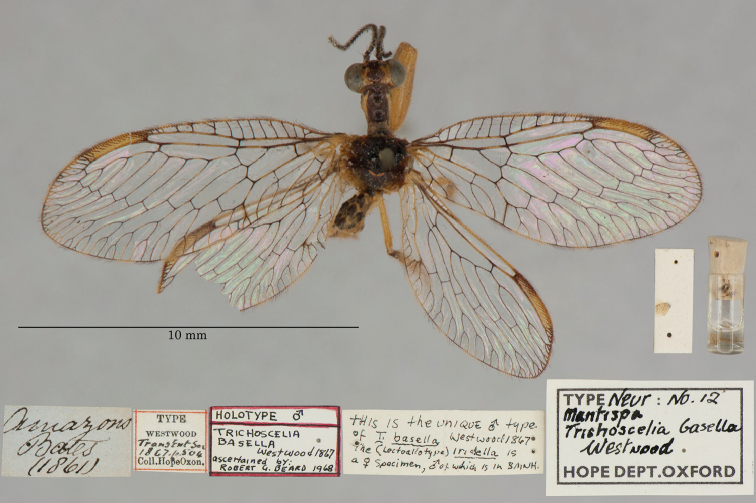
Holotype of Mantispa (Trichoscelia) basella Westwood, 1867 (NEUR0012, male).

**2. *basella* Westwood, 1867** [Mantispa (Trichoscelia)] (Holotype; Fig. 35)

**Original description.***Trans. R. Entomol. Soc. Lond., 15: 504*; “(Mas.) [Male] … Habitat in Amazonia. D. Bates. In Mus. Oxon.”.

**Type series.** Westwood did not explicitly identify the number of specimens in the type series. However, he designated only one depository, and he referred to one male specimen. Currrently, a single type (male) is in the OUMNH (NEUR0012); it was labeled as the holotype by R. G. Beard in 1968 (Fig. 35). Later, Penny (1982b: 431) and Ohl (2004: 149) examined the specimen and confirmed it as the holotype. Oswald (2018) referred to it as the holotype (by implicit monophyly).

**Current name.***Trichosceliairidella* (Westwood, 1867).

**Nomenclature.**Penny (1982b: 431) proposed the synonymy; it was listed by Ohl (2004: 149).

**Figure 36.**
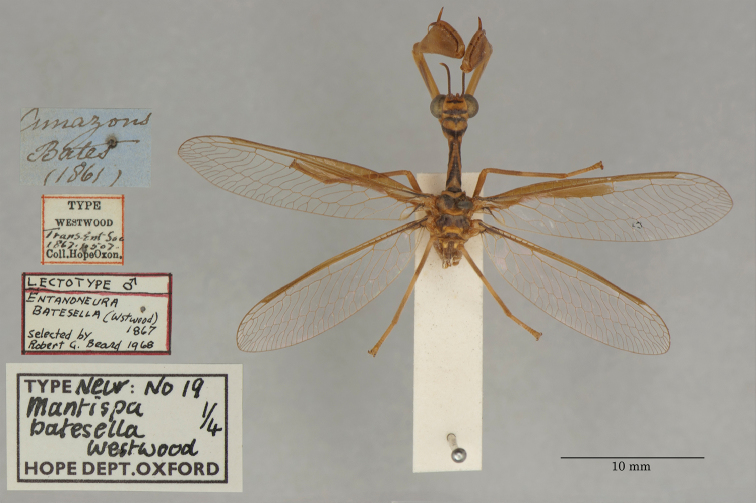
Lectotype (new designation) of *Mantispabatesella* Westwood, 1867 (NEUR0019-01, male).

**Figure 37.**
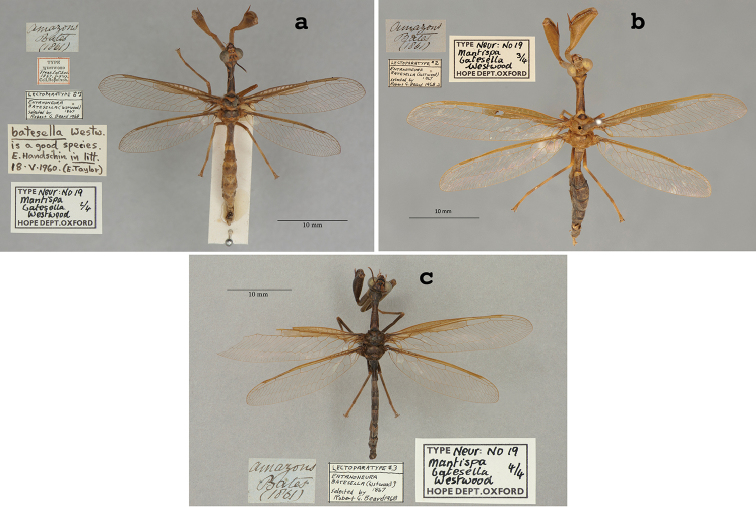
Three of three OUMNH paralectotypes of *Mantispabatesella* Westwood, 1867 **a** NEUR0019-02, male **b** NEUR0019-03, female **c** NEUR0019-04, sex undetermined.

**3. *batesella* Westwood, 1867** (*Mantispa*) (Lectotype, new designation; three paralectotypes; Figs 36, 37)

**Original description.***Trans. R. Entomol. Soc. Lond., 15: 507*; “(Mas et foem.) [Male and female] … Habitat in Amazonia. D. Bates. In Mus. Oxon.”. Sexes and number of specimens not specified.

**Type series.** Westwood’s description did not specifically state the number of types he studied. However, he did mention seeing a male and a female, so there was more than one. Penny (1982b: 448) and Penny and de Costa (1983: 668) stated that Westwood’s types were not in the OUMNH, but that they might be in the NHMUK. However, Ohl (2004: 169) and Ardila-Camacho and García (2015: 441) later reported seeing types in the OUMNH.

Four syntypes are held in the OUMNH. We did not find any in the NHMUK. In 1986, R. G. Beard applied a lectotype label to one of the OUMNH specimens, a male (NEUR0019-01, Fig. 36), and paralectotype labels to the three others: one male (NEUR0019-02, Fig. 37a), one female (NEUR0019-03, Fig. 37b), and one unknown sex (NEUR0019-04, Fig. 37c). However, he did not publish his designations. Here we confirm Beard’s choice of the above male OUMNH NEUR0019-01 as the lectotype (present designation) of *Mantispabatesella* Westwood. The other three specimens become paralectotypes as labeled.

**Current name.***Entanoneurabatesella* (Westwood, 1867).

**Nomenclature.** The first use of the current combination was by Enderlein (1910: 359) [cited by Handschin (1960b: 536), Penny and de Costa (1983: 665), Ardila-Camacho and García (2015: 441), and Ardila-Camacho et al. (2018: 321)]. Note: According to the Lacewing Digital Library (Oswald 2018), the species was also known previously as *Sagittalatabatesella* (Westwood). We did not find a reference for this combination, nor is it listed by Ohl (2004).

**Figure 38.**
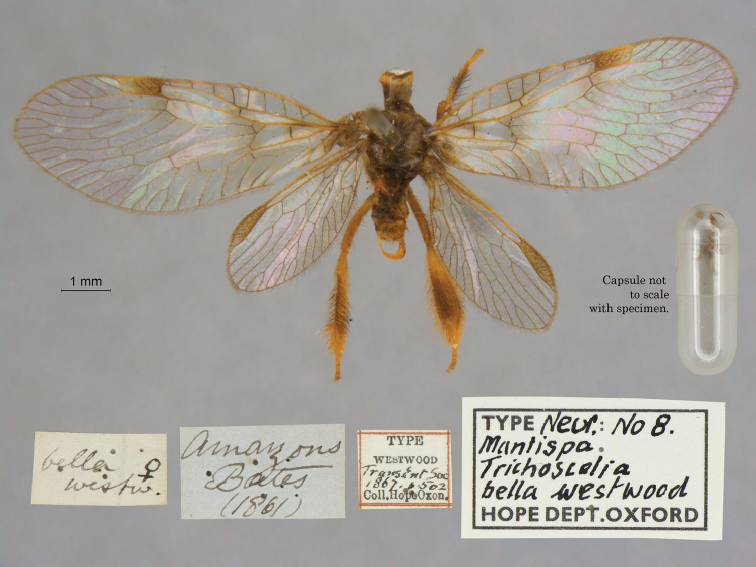
Holotype of Mantispa (Trichoscelia) bella Westwood, 1867 (NEUR0008, female).

**4. *bella* Westwood, 1867** [Mantispa (Trichoscelia)] (Holotype; Fig. 38)

**Original description.***Trans. R. Entomol. Soc. Lond., 15: 502*; “(Foem.) [Female] … Habitat in Amazonia. D. Bates. In Mus. Oxon.”.

**Type series.** Although Westwood’s description did not specifically state how many type specimens he had, he referred to a female, and he designated only one depository.

A single type (female) is in the OUMNH (NEUR0008, Fig. 38). It was idenitfied as the holotype by Penny (1982b: 418) and subsequently listed as such by Ohl (2004: 146). Oswald (2018) also listed it as the holotype (by implicit monotypy).

**Current name.***Anchietabella* (Westwood, 1867).

**Nomenclature.***Anchieta* became the generic replacement name after *Anisoptera* Schneider was identified as a junior homonym (Penny 1982a: 216–217). The first use of the current combination was by Penny (1982b: 418). The species was also previously known as *Trichosceliabella* (Westwood) [generic assignment by Enderlein (1910: 376), but without use of the combination] and *Anisopterabella* (Westwood) [generic assignment by Gerstaecker (1887: 117), without use of the combination].

**Figure 39.**
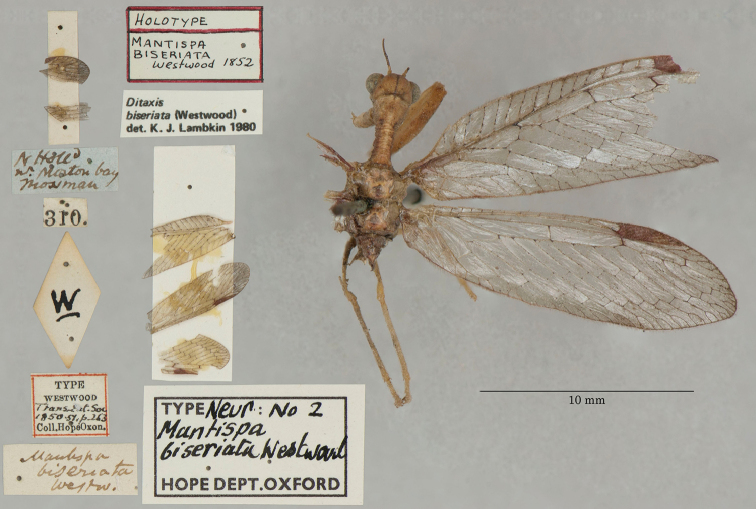
Lectotype of *Mantispabiseriata* Westwood, 1852 (NEUR0002, sex undetermined).

**5. *biseriata* Westwood, 1852** (*Mantispa*) (Lectotype; Fig. 39)

**Original description.***Trans. R. Entomol. Soc. Lond., 6 [1]: 263, tab. 17, fig. 7*; “Habitat in Australia, Moreton Bay. D. Mossman. Mus. Westw.”. Sexes and number of specimens not specified.

**Type series.** We found no information on how many type specimens Westwood had, nor the sex(es). However, his original description does indicate one depository, his personal collection.

One type, in relatively poor condition and with sex unknown, is in the OUMNH (NEUR0002, Fig. 39). It carries a holotype label applied by R. G. Beard in 1968. Lambkin (1986a: 36) identified this specimen as the holotype and provided full information on its condition. Subsequently, Oswald (2018) listed it as the holotype (by implicit monotypy). However, none of the above individuals stated how he determined that the specimen was the sole member of Westwood’s type series or if it was Westwood’s choice as the primary type among syntypes.

In addition to the specimen in the OUMNH, a specimen in the NHMUK carries a label that is typical of NHMUK type specimens (circular, with a red margin). Apparently, this is the specimen that Ohl (2004: 151) listed as the holotype. However, it does not carry original locality or other labels that identify it as a type, and we cannot confirm type status for it. R. G. Beard’s label on this specimen (NHMUK) states that he did not consider it to be a primary type, but rather a male topotype. We agree with Beard’s conclusion here.

Thus, we now know that the single OUMNH specimen is the only verified name-bearing type for this species, but we see a lack of support either for or against monotypy. It appears that Lambkin's identification of the OUMNH specimen as the holotype serves to fix it as the lectotype, by subsequent designation.

**Current name.***Ditaxisbiseriata* (Westwood, 1852).

**Nomenclature.** Originally, Brauer (1867: 285) transferred *M.biseriata* to the genus *Drepanicus* Blanchard. However, McLachlan (1868b: 262) felt that the species warranted a separate genus, and he assigned the current combination. Lambkin (1986a: 36) provides a full list of synonymies.

**Figure 40.**
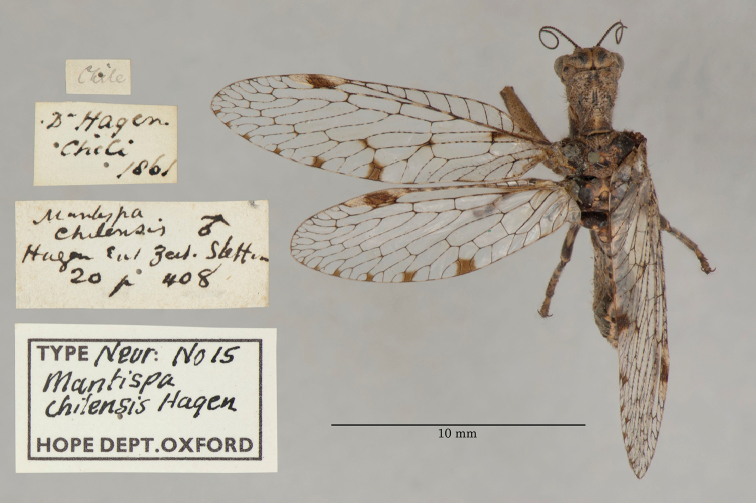
OUMNH syntype of *Mantispachilensis* Hagen, 1859 (NEUR0015, male).

**6. *chilensis* Hagen, 1859** (*Mantispa*) (Syntype; Fig. 40)

**Original description.***Entomol. Zeit., 20: 408*; “Patria Chili. Zwei Stücke von Dohrn mitgetheilt.”.

**Type series.** In his original description, Hagen stated that he received two specimens from Dohrn, one of which probably was a male and the primary specimen that he described. Apparently, that specimen remained in his own collection in the MCZ. He sent the other specimen, which he reported as larger and possibly a female, to Westwood perhaps even before he wrote his description of *M.chilensis*. Ohl (2004: 152 and Footnote 15) noted Hagen’s report of two specimens, and he identified the type in the MCZ as a syntype (MCZT-10428; images not available, sex not reported). The whereabouts of the second specimen has long been unknown.

There is a male specimen in the OUMNH (NEUR0015, Fig. 40) that is identified as a type, and that carries locality data and other labels consistent with the original description. Given that this specimen is male, and that neither it nor the type in the MCZ have been studied in detail, for now we consider them to be syntypes. A lectotype has not been selected.

**Current name.***Gerstaeckerellachilensis* (Hagen, 1859).

**Nomenclature.** The current combination was proposed by Enderlein (1910: 373), confirmed by Banks (1913a: 207), and listed by Penny (1977: 34). The species previously resided in *Symphrasis* Hagen (see Hagen 1877: 211, Enderlein 1910: 373), and later in *Anisoptera* Schneider, after *Symphrasis* was synonymized with *Anisoptera* (see Gerstaecker 1887: 117; Penny 1982a: 212).

**Figure 41.**
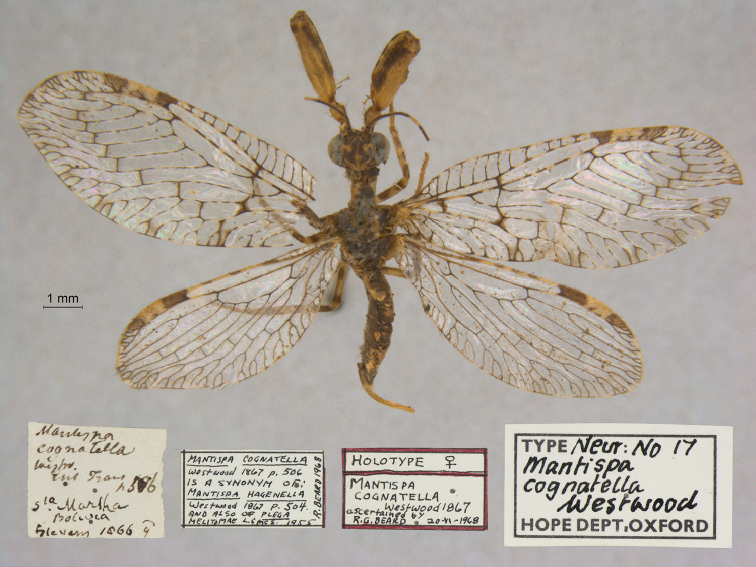
Lectotype of *Mantispacognatella* Westwood, 1867 (NEUR0017, female).

**Figure 42.**
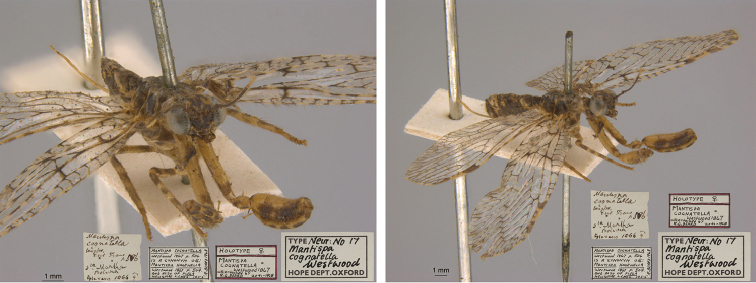
Lectotype of *Mantispacognatella* Westwood, 1867 (NEUR0017, female) **a** frontolateral view **b** dorsolateral view.

**7. *cognatella* Westwood, 1867** (*Mantispa*) (Lectotype; Figs 41, 42)

**Original description.***Trans. R. Entomol. Soc. Lond., 15: 506*; “Habitat apud Sanctam Martham, Venezuelae. In Mus. Oxon.”. Sexes and number of specimens not specified.

**Type series.** There is one Westwood specimen, a female, in the OUMNH (NEUR0017, Figs 41, 42). In 1968, R. G. Beard identified this specimen as the holotype, and both Penny (1982b: 425) and Ohl (2004: 148) refer to it as the holotype. None of these three individuals provided any information as to why they considered this specimen to be the only one that Westwood had in the type series. In the absence of any evidence for monotypy, we conclude that Penny’s identification of the OUMNH specimen as the holotype served to fix it as the lectotype, by subsequent designation.

The information on the locality label of the OUMNH specimen differs somewhat from that reported by Westwood. The label reads: “Sta. Martha Bolivia”, not “Sanctam Martham, Venezuelae” as reported in the original description (see Figs 41, 42). Penny (1982b: 425) noted the anomaly; he suggested that specimens with such locality data might be from Santa Marta, a colonial city on Colombia’s Caribbean coast near Venezuela; this site was frequented by insect collectors.

**Current name.***Plegahagenella* (Westwood, 1867).

**Nomenclature.**Penny (1982b: 425) proposed the synonymy with *Plegahagenella* (Westwood, 1867); it was listed by Ohl (2004: 148) and confirmed by Ardila-Camacho and García (2015: 415), using OUMNH images of types. The species was also known as *Symphrasiscognatella* (Westwood) [combination by Hagen (1877: 211); see Oswald (2018)].

**Figure 43.**
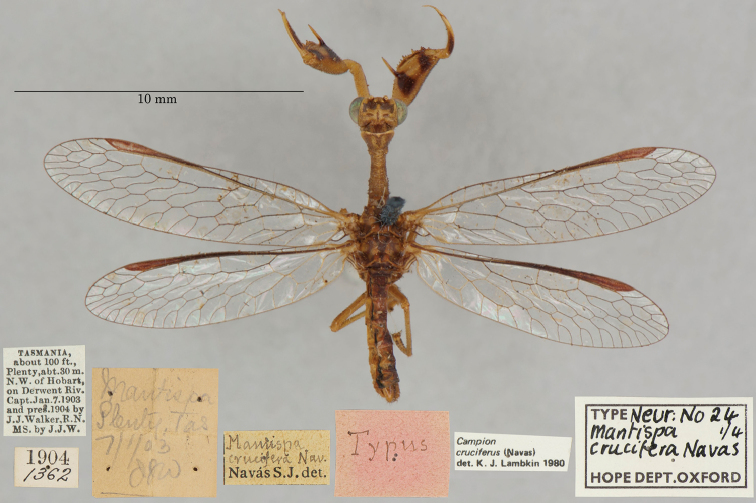
Lectotype of *Mantispacrucifera* Navás, 1914 (NEUR0024-01, male).

**Figure 44.**
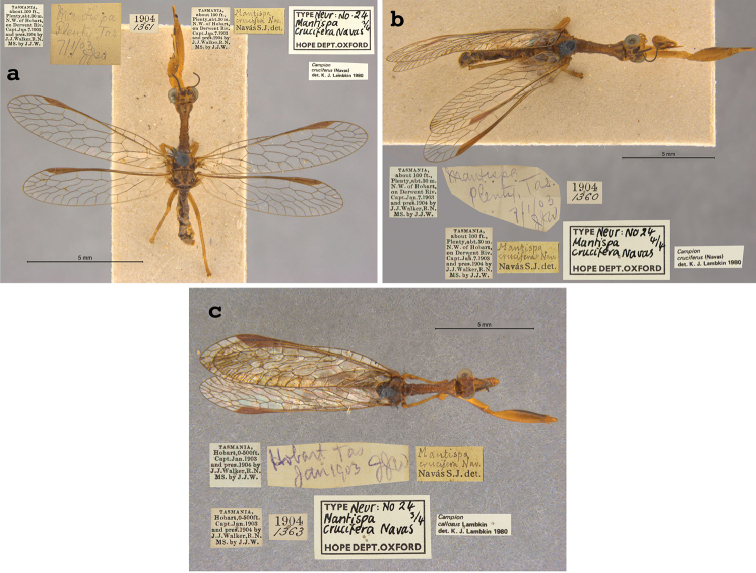
Three OUMNH paralectotypes of *Mantispacrucifera* Navás, 1914, **a** NEUR0024-02 (male) **b** NEUR0024-04 (male) **c** NEUR0024-03 (sex undetermined), now considered not to be conspecific with the lectotype.

**8. *crucifera* Navás, 1914** (*Mantispa*) (Lectotype, three paralectotypes; Figs 43, 44)

**Original description.***Bol. Soc. Aragonesa Cien. Nat., 1914c, 13: 61*; “‘Tasmania, about 100 ft. Plenty, abt. 30 m. N. W. of Hobart, on Derwent Riv.’ Julio 1902, J. J. Walker.”. Sexes and number of specimens not specified.

**Type series.** From Navás’ statements in the description, clearly his type series contained more than one specimen, and his specimens showed variation in venation and size. Lambkin (1986b: 22) identified four specimens in the type series. The OUMNH has all four, and they have identification labels with Navás’ handwriting. Only one specimen (a male) carries a Navás “Type” label, and Lambkin identified this male as the holotype (NEUR0024-01, Fig. 43). He identified two other male specimens as paratypes (NEUR0024-02, NEUR0024-04; Fig. 44a, b), and a fourth specimen (NEUR0024-03, Fig. 44c) as belonging to another species: *Campioncallosus* Lambkin. It is labeled as such.

Here we are in agreement with Ohl (2004: 163 and Footnote 33) who noted that Lambkin’s holotype identification served to fix the lectotype designation. This designation is justified, because Lambkin (1986b: 22) clearly indicated that he selected a specific specimen as the holotype over the other syntypes available. Thus, ICZN Article 74.5 applies to his use of the term holotype, and the lectotype designation is valid. The remaining specimens (NEUR0024-02, -03, -04), including the misidentified one, are all regarded as paralectotypes.

**Current name.***Campioncruciferus* (Navás, 1914).

**Nomenclature.** The current combination was proposed by Lambkin (1986b: 21) and listed by Ohl (2004: 163).

**Figure 45.**
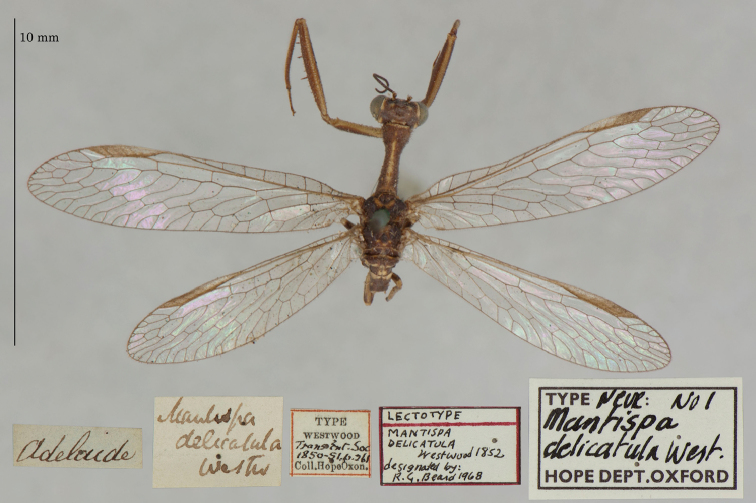
Lectotype of *Mantispadelicatula* Westwood, 1852 (NEUR0001-01, sex undetermined).

**Figure 46.**
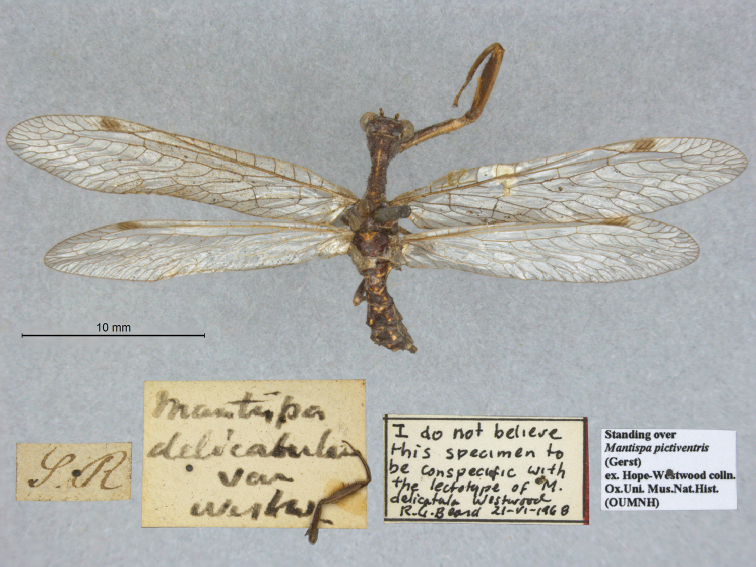
OUMNH paralectotype of *Mantispadelicatula* Westwood, 1852 (NEUR0001-02, sex undetermined), now considered not to be conspecific with the lectotype.

**9. *delicatula* Westwood, 1852** (*Mantispa*) (Lectotype, one paralectotype; Figs 45, 46)

**Original description.***Trans. R. Entomol. Soc. Lond., 6 [1]: 261, Tab. nostr. 17, fig. 5*; “Habitat apud Adelaidam. D. Fortnum. In Mus. Hope.”. Sexes and number of specimens not specified.

**Type series.** Westwood cited one depository, the OUMNH, and he noted that the specimens he studied displayed a substantial range of variation in body size, coloration, and wing venation. For example, note the size range of the scale bars on fig. 5 of Tab. nostr. 17 and the comments in the last paragraph of the original description. Lambkin (1986a: 62) reported two specimens in the OUMNH. Later, Banks (1939: 479) reported a type for this species in the Hagen collection at the MCZ (currently listed in the MCZ Type Database, type 10420; see Ohl 2004: Footnote 19). Therefore, we know that there were at least two, and probably more, specimens in Westwood’s type series. He did not specify a primary type.

We found the two aforementioned types in the OUMNH. The body length of the smaller one (NEUR0001-01, Fig. 45) is similar to that measured by Westwood for his smaller specimen. In 1968, R. G. Beard labeled this specimen as the lectotype (sex unknown, abdomen missing). However, he never published his lectotype designation. Later, Lambkin (1986a: 62) identified it as the holotype – an action that fixed the specimen as the lectotype (ICZN Article 74.5).

The second type in the OUMNH (NEUR0001-02, Fig. 46), sex undetermined, is considerably larger than the first; it corresponds in size with the body length that Westwood reported for his larger specimen. This specimen carries a label reading “*Mantispadelicatula* var Westw”, as well as another label applied by R. G. Beard stating his opinion that this specimen was not conspecific with the first specimen. Indeed, Lambkin (1986a: 62) did not consider the larger, second specimen to be among the type series. Rather, he quoted Dr. M. W. R. de V. Graham who told him that this specimen never stood with the type specimen, that it was labeled “*delicatula* Westw. Var.”, and that it should not be considered a syntype.

Later, Ohl (2004: Footnote 19) disagreed with Lambkin’s interpretation. He felt that because Westwood’s description included information from more than one specimen, and that it cited a substantial range of size and color variation, the larger specimen should be included as a syntype. Although we found the large Westwood specimen (NEUR0001-02) in the main collection, not among the OUMNH types, we agree with Ohl’s stance and confirm that the situation is in conformance with Article 72.4.1 that requires the specimen to be included as a syntype. Westwood wrote “Var.” after the species name on his determination label, but there is no indication that he doubted the species identity of this specimen, or that he intended to exclude it from the type series. As a result, we agree with Ohl (2004: Footnote 19) that Westwood’s second specimen (NEUR0001-02), whether conspecific with the lectotype or not, is part of the type series, and we identify it as a paralectotype of *M.delicatula*.

We found one old specimen of *M.delicatula* in the NHMUK (NHMUK011250016). Although it has locality data that are consistent with the type series, it is not labeled as a type, and its possible paralectotype status needs further investigation.

**Current name.***Theristriadelicatula* (Westwood, 1852).

**Nomenclature.** The current combination was proposed by Gerstaecker (1884: 44), confirmed by Banks (1913a: 207), and listed by Ohl (2004: 154). See Lambkin (1986a: 61) for a full synonymy.

**Figure 47.**
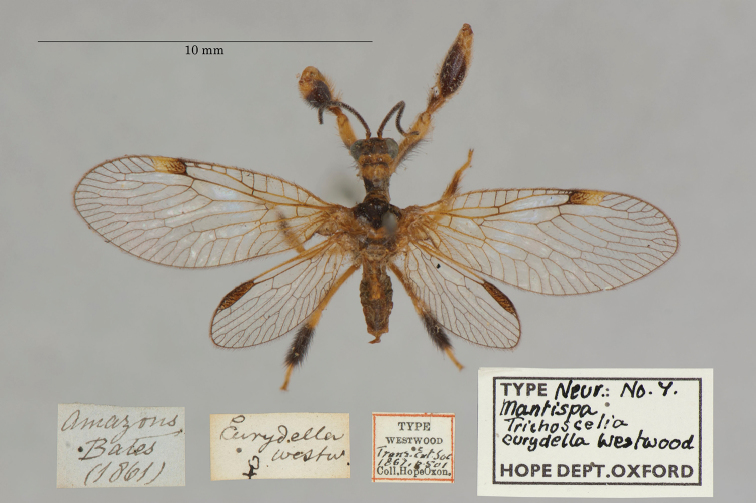
Holotype of Mantispa (Trichoscelia) eurydella Westwood, 1867 (NEUR0007, female).

**10. *eurydella* Westwood, 1867** [Mantispa (Trichoscelia)] (Holotype; Fig. 47)

**Original description.***Trans. R. Entomol. Soc. Lond., 15: 501*; “Foem. [Female] … Habitat in Amazonia. D. Bates. In Mus. Oxon.”.

**Type series.** Westwood’s description mentioned a female specimen and designated one depository. We found only one type in the OUMNH (NEUR0007, Fig. 47), and it is a female. Penny (1982b: 419), Ohl (2004: 146), and Ardila-Camacho and García (2015: 408) identified the specimen as the holotype, and Oswald (2018) listed it as the holotype (by implicit monotypy).

**Current name.***Anchietaeurydella* (Westwood, 1867).

**Nomenclature.** The current combination was offered by Penny (1982b: 419); cited by Ohl (2004: 146), Ardila-Camacho and García (2015: 408), and Ardila-Camacho et al. (2018: 321). The species was previously known as *Trichosceliaeurydella* Westwood [combination by Enderlein (1910: 376)] and *Anisopteraeurydella* (Westwood) [generic assignment by Gerstaecker (1887: 117), but without use of the combination]. *Anchieta* became the replacement name after *Anisoptera* Schneider was identified as a junior homonym (see Penny 1982a: 216–217).

**Figure 48.**
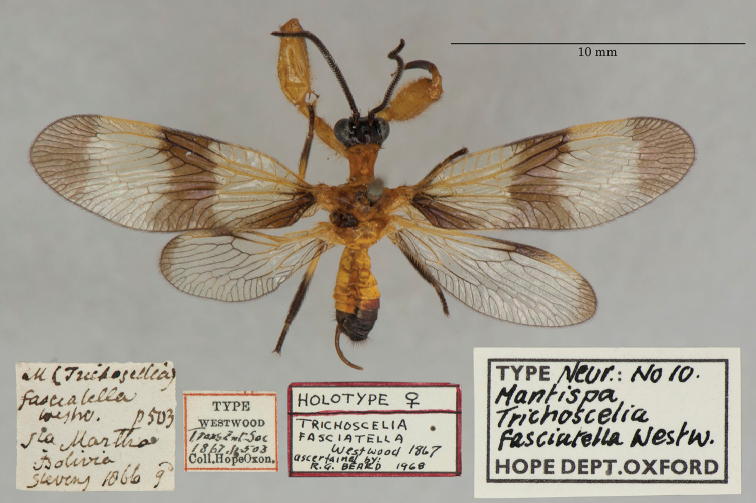
Holotype of Mantispa (Trichoscelia) fasciatella Westwood, 1867 (NEUR0010, female).

**11. *fasciatella* Westwood, 1867** [Mantispa (Trichoscelia)] (Holotype; Fig. 48)

**Original description.***Trans. R. Entomol. Soc. Lond., 15: 503*; “(Foem.) [Female] … Habitat apud Sanctam Martham, Venezuelae. In Mus. Oxon.”.

**Type series.** Westwood’s description did not state specifically how many type specimens he had. However, he mentioned one female, and he designated one depository, the OUMNH. The collection holds only one type of this species, a female (NEUR0010, Fig. 48). R. G. Beard had labeled it as the holotype, and Ohl (2004: 147), Ardila-Camacho and García (2015: 413), and Oswald (2018) listed both holotype and syntype assignations as possible. However, to be consistent with the other Westwood species described at the same time and in the same manner (e.g., *M.basella*, *M.bella*, *M.eurydella*, *M.fumosella*, *M.hagenella*, *M.sequella*), this specimen also should be considered a holotype (by implicit monotypy).

The specimen in the OUMNH carries labels that identify it as a type, but some of the information on the locality label differs from that in the original description. The label reads: “Bolivia", not "Venezuelae" as in the description. Penny (1982b: 425) noted the same anomaly for types of *Mantispacognatella* (Westwood) (above). He suggested that specimens carrying such locality data might be from the colonial city of Santa Marta, a frequent collecting site on Colombia’s Caribbean coast near Venezuela.

**Current name.***Anchietafasciatella* (Westwood, 1867).

**Nomenclature.**Penny (1982b: 422), Ohl (2004: 147), and Ardila-Camacho and García (2015: 413) included the species in the genus *Plega*. However, recently, Ardila-Camacho et al. (2018: 297) demonstrated that it belongs in the genus *Anchieta*. The species also was previously known as *Trichosceliafasciatella* Westwood [combination by Enderlein (1910: 376)] and *Anisopterafasciatella* (Westwood) [generic assignment by Gerstaecker (1887: 117), but the combination apparently was unused and the generic name is a junior homonym (Penny 1982a: 216–217)].

**Figure 49.**
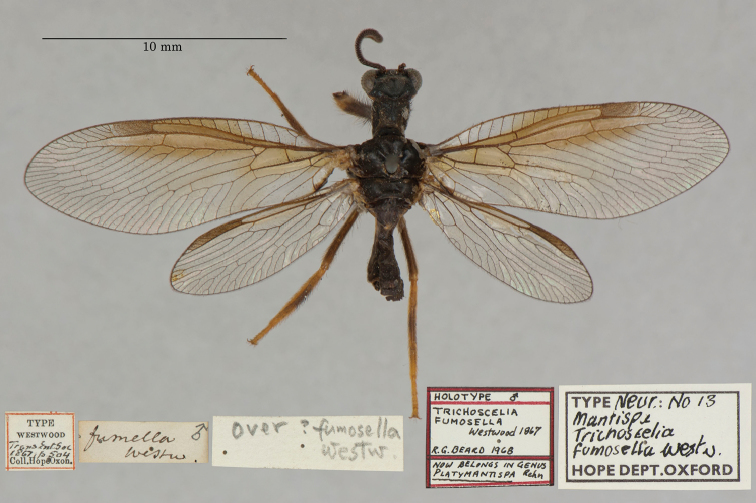
Holotype of Mantispa (Trichoscelia) fumosella Westwood, 1867 (NEUR0013, male).

**12. *fumosella* Westwood, 1867** [Mantispa (Trichoscelia)] (Holotype; Fig. 49)

**Original description.***Trans. R. Entomol. Soc. Lond., 15: 504*; “(Mas.) [Male] … Habitat in Amazonia. D. Bates. In Mus. Oxon.”.

**Type series.** Westwood’s description did not state specifically how many type specimens he had; however, he mentioned studying a male specimen, and he designated a single depository, the OUMNH. One type, a male, is in the OUMNH (NEUR0013, Fig. 49). It was labeled as the holotype by R. G. Beard in 1968, and later Penny (1982b: 420) referred to it as a “male holotype”. Subsequently, both Ohl (2004: 146) and Oswald (2018) indicated that holotype or syntype designations are possible. To be consistent with other specimens described by Westwood in the same publication and in the same manner (e.g., *M.fasciatella*, above), this specimen should be considered the holotype (by implicit monotypy).

**Current name.***Anchietafumosella* (Westwood, 1867).

**Nomenclature.** The current combination was proposed by Penny (1982b: 419) and listed by Ohl (2004: 146). The species was previously known as *Trichosceliafumosella* Westwood [generic assignment by Enderlein (1910: 376), but combination not used] and *Anisopterafumosella* (Westwood) [generic assignment by Gerstaecker (1887: 117), but combination not used]. *Anchietafumosella* (Westwood) became the replacement name after *Anisoptera* Schneider was identified as a homonym (see Penny 1982a: 216–217).

**Figure 50.**
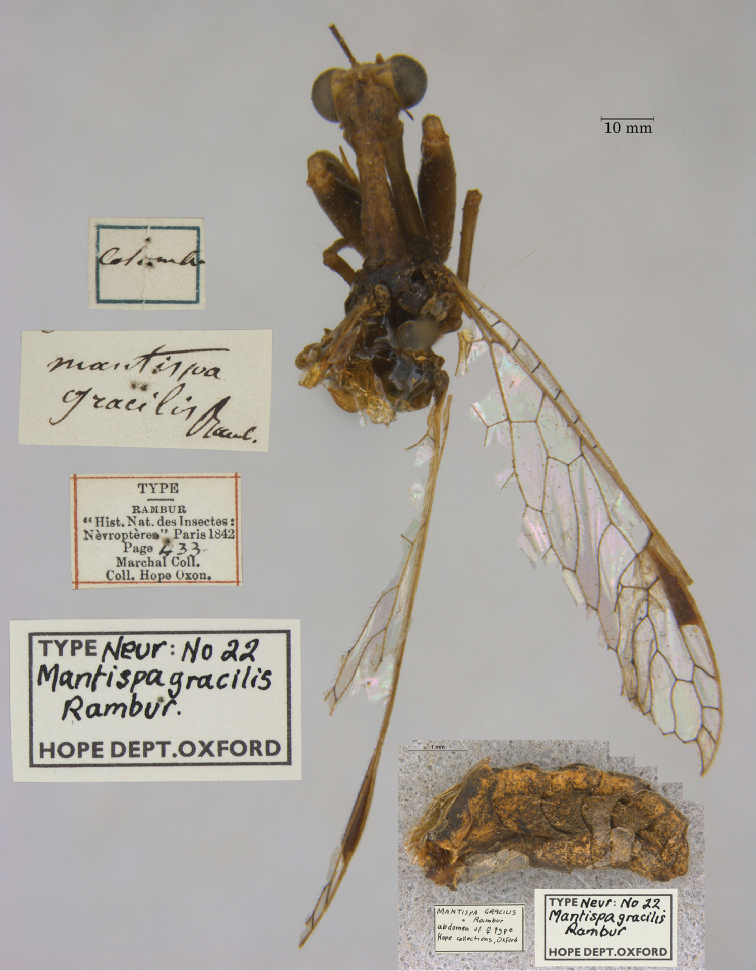
OUMNH syntype of *Mantispagracilis* Rambur, 1842 (NEUR0022, female).

**13. *gracilis* Rambur, 1842** (*Mantispa*) (One syntype; Fig. 50)

**Original description.***Libr. encycl. Roret, 1842: 433*; “Habite, dans la Colombie, les environs de Santa-Fé-de-Bogota.”. Sexes and number of specimens not specified.

**Type series.** A type (sex unknown) is reported from the IRSNB, Brussels (Ohl 2004: 184, Oswald 2018). We did not confirm this report. The OUMNH holds one syntype, a female in poor condition (NEUR0022, Fig. 50). It has not received much attention in the literature. Apparently, no lectotype has been designated.

**Current name.***Mantispairidipennis* Guérin-Méneville, 1844.

**Nomenclature.** A tentative synonymy was offered by Westwood (1852: 256); subsequently it was listed by Penny (1977: 35) and Ohl (2004: 184). The original name is a junior homonym of *Mantispagracilis* Erichson, 1839 [now *Dicromantispagracilis* (Erichson, 1839)] (see Oswald 2018).

**Figure 51.**
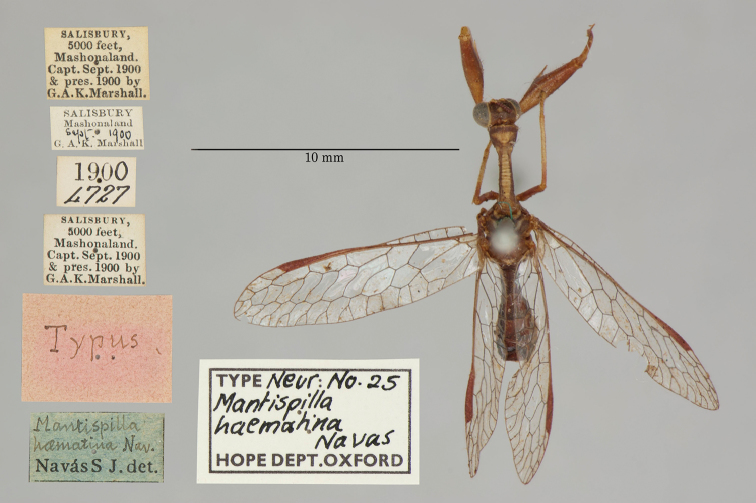
Holotype of *Mantispillahaematina* Navás, 1914 (NEUR0025, female).

**14. *haematina* Navás, 1914** (*Mantispilla*) (Holotype; Fig. 51)

**Original description.***Bol. Soc. Aragonesa Cien. Nat., [1914c] 13: 62*; “Africa meridional: ‘Salisbury, 5.000 feet, Mashonaland’. Sept. 1900, G. A. K. Marshall.”. Number of specimens not specified.

**Type series.** Navás’ article (by title) is focused on specimens in the OUMNH, and it did not state specifically how many type specimens of this species he had. However, the original description gave measurements for a single female. We found one type with a Navás type label in the OUMNH, a female (NEUR0025, Fig. 51). Ohl (2004: 183) identified it as the holotype, and Oswald (2018) referred to holotype or syntype assignments as being possible. To be consistent with other mantispid species treated here (mostly described by Westwood), we consider the specimen to be a holotype (by implicit monotypy).

**Current name.***Mantispahaematina* (Navás, 1914).

**Nomenclature.***Mantispilla* was considered a subgenus of *Mantispa* by Enderlein (1910: 346), and the two generic level names, *Mantispilla* and Mantispa (Mantispilla), were synonymized under *Mantispa* by Penny (1982a: 217). Ohl (2004: 183) probably made the first subsequent use of the current combination.

**Figure 52.**
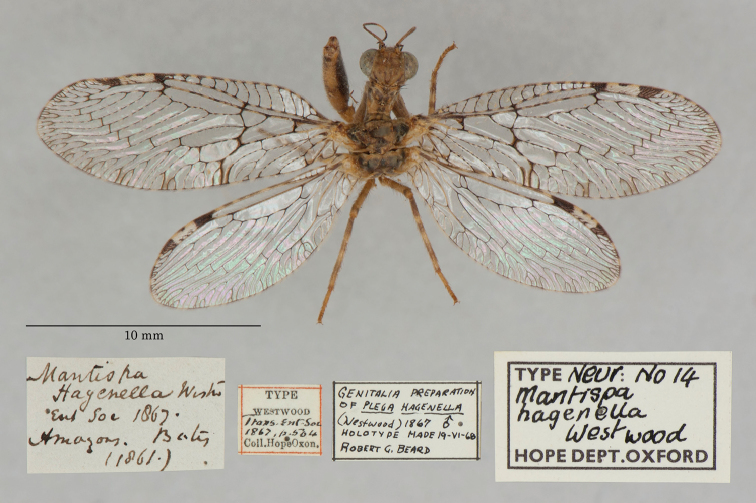
Holotype of *Mantispahagenella* Westwood, 1867 (NEUR0014, male).

**Figure 53.**
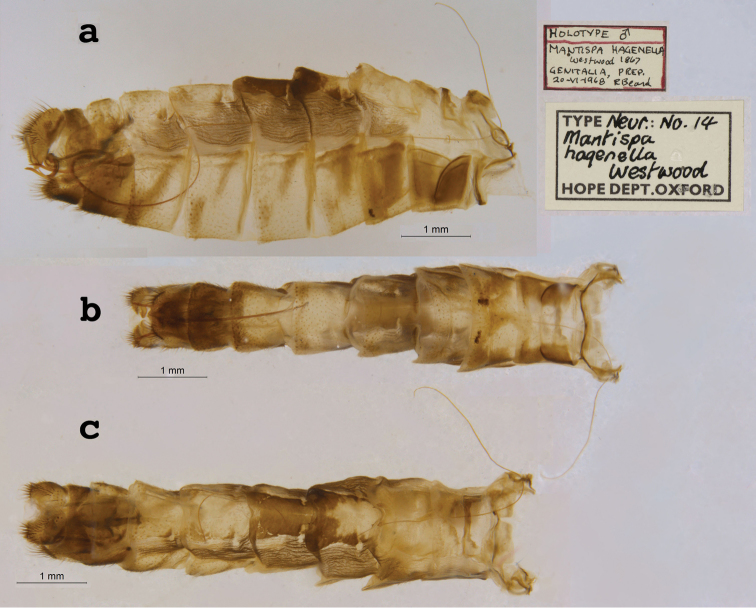
Holotype of *Mantispahagenella* Westwood, 1867 (NEUR0014, male), cleared abdomen **a** lateral **b** ventral **c** dorsal.

**15. *hagenella* Westwood, 1867** (*Mantispa*) (Holotype; Figs 52, 53)

**Original description.***Trans. R. Entomol. Soc. Lond., 15: 504*; “(Mas.) [Male] … Habitat in Amazonia. D. Bates. In Mus. Oxon.”.

**Type series.** Although Westwood’s description did not state specifically how many type specimens he used, he specified a single depository, the OUMNH, and he mentioned one male specimen. In 1968, R. G. Beard labeled the specimen (NEUR0014, Figs 52, 53) as the holotype. He also cleared the abdomen, which is preserved in a separate vial. Later, Penny (1982b: 425) referred to the specimen as the holotype; subsequently Ohl (2004: 148) and Ardila-Camacho and García (2015: 415) listed it as the holotype, and Oswald (2018) identified it as the holotype (by implicit monotypy).

**Current name.***Plegahagenella* (Westwood, 1867).

**Nomenclature.** The current combination is by Penny (1982b: 425); it was listed by Ohl (2004: 148), Ardila-Camacho and García (2015: 415), and Ardila-Camacho et al. (2018: 298). The species was also known previously as *Gerstaeckerellahagenella* (Westwood) [combination by Enderlein (1910: 370)].

The Lacewing Digital Library (Oswald 2018) lists the combination *Trichosceliahagenella* (Westwood) as a synonym of this species. In his original description, Westwood (1867: 504) expressly excluded *M.hagenella* from subgenus Trichoscelia, and we have not seen the combination used elsewhere. Neither Ohl (2004: 148), Ardila-Camacho and García (2015: 415), nor Ardila-Camacho et al. (2018: 298) listed the combination among the synonyms of *P.hagenella*.

**Figure 54.**
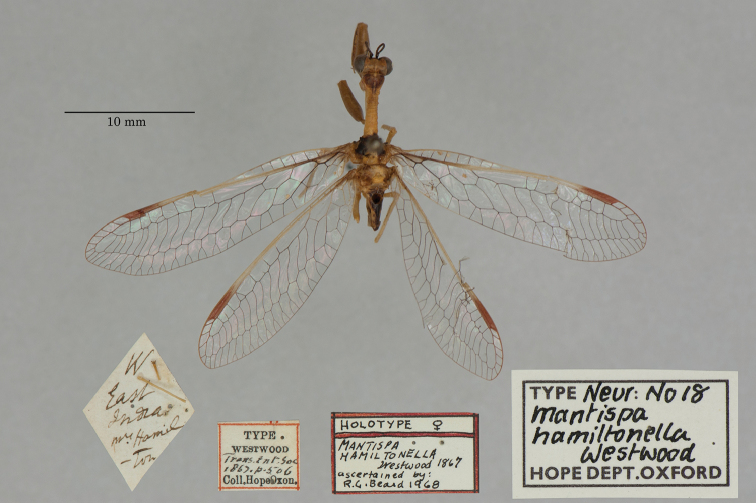
OUMNH syntype of *Mantispahamiltonella* Westwood, 1867 (NEUR0018, female).

**16. *hamiltonella* Westwood, 1867** (*Mantispa*) (Syntype; Fig. 54)

**Original description.***Trans. R. Entomol. Soc. Lond., 15: 506*; “Habitat in India orientali. Dna. Hamilton. In Mus. Oxon.”. Sexes and number of specimens not specified.

**Type series.** A single type, a female, is in the OUMNH (NEUR0018, Fig. 54). It was labeled as the holotype in 1968 by R. G. Beard. Subsequently, Ohl (2004: 162) and Oswald (2018) stated that one or more syntypes may exist. Given the lack of any supporting evidence of monotypy, we consider that syntypes are possible.

**Current name.***Campanacellahamiltonella* (Westwood, 1867).

**Nomenclature.** The genus name was most recently reassigned by Handschin (1961: 280) and listed by Ohl (2004: 162). The species was also known as *Entanoneurahamiltonella* (Westwood) [combination by Enderlein (1910: 359)] and *Eumantispahamiltonella* (Westwood) [name used by Banks (1934: 568), but not cited as a new combination].

**Figure 55.**
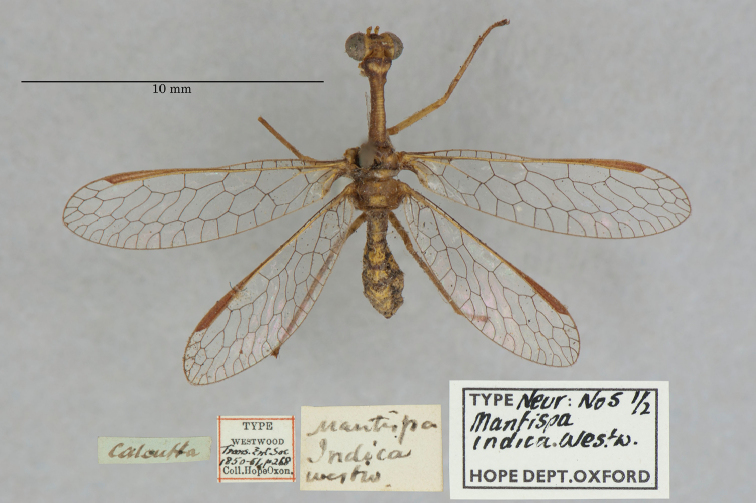
First of two OUMNH paralectotypes for *Mantispaindica* Westwood, 1852 (NEUR0005-01, probably female).

**Figure 56.**
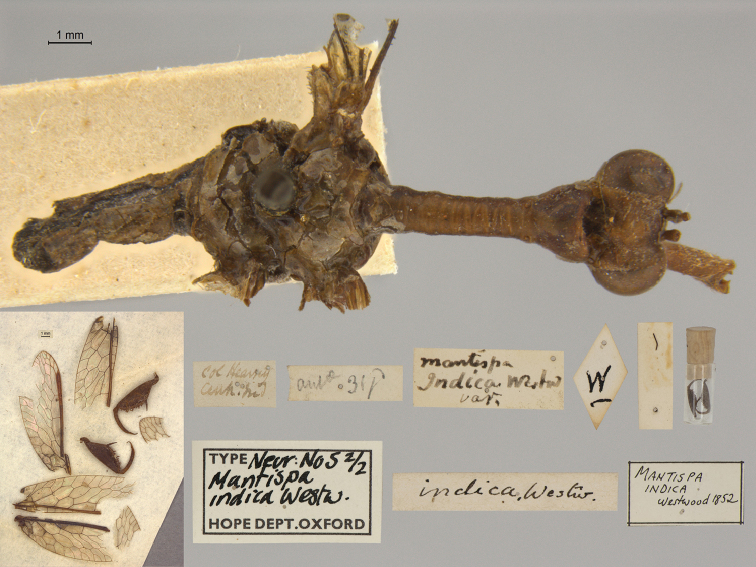
Second of two OUMNH paralectotypes for *Mantispaindica W*estwood, 1852 (NEUR0005-02, sex undetermined).

**17. *indica* Westwood, 1852** (*Mantispa*) (Two paralectotypes; Figs 55, 56)

**Original description.***Trans. R. Entomol. Soc. Lond., 6 [1]: 268, tab. 18, fig. 5*; “Habitat in India orientali, Calcutta, Nepalia. (D. Hardwicke, &c.) In Mus. Britann., Westwood.”. Sexes and number of specimens not specified.

**Type series.** Westwood’s description mentioned two depositories (British Museum and his own collection), and Ohl (2004: 184) reported seeing types at the OUMNH and the NHMUK. Two types (sexes unconfirmed, one probably female) are in the OUMNH (NEUR0005-01, -02; Figs 55, 56).

**New lectotype designation**, NHMUK specimen. We also found a type identified as the lectotype by R. G. Beard in the NHMUK; however, his designation was never published. Here, we designate this specimen (labeled male) as the lectotype (present designation). Its labels read: (1) “Type”, (2) “Nepal” on one side, “Hardwicke Beq” on the other side, (3) “indica Westw”, (4) “LECTOTYPE ♂, MANTISPA INDICA Westwood 1852, designated by R. G. Beard 1968”, (5) “BMNH(E) 1253432”. With the above designation, the two specimens in the OUMNH become paralectotypes.

**Current name.***Mantispaindica* Westwood, 1852.

**Nomenclature.** The name is now the same as the original, and it is listed as such by Ohl (2004: 184). For a while, the species was known as Mantispa (Mantispilla) indica (Westwood) [combination by Enderlein (1910: 346)]. Later, the name *Mantispilla* was synonymized with *Mantispa* by Penny (1982a: 217).

**Figure 57.**
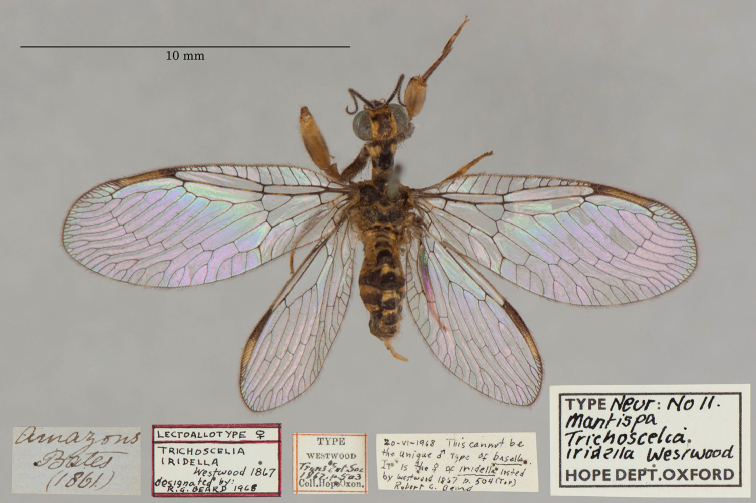
OUMNH paralectotype of Mantispa (Trichoscelia) iridella Westwood, 1867 (NEUR0011, female).

**18. *iridella* Westwood, 1867** [Mantispa (Trichoscelia)] (One paralectotype; Fig. 57)

**Original description.***Trans. R. Entomol. Soc. Lond., 15: 503*; “(Mas et foem.) [Male and female] … Habitat in Amazonia. D. Bates. In Muss. Brit. et Oxon.”.

**Type series.** Westwood’s description did not state specifically how many type specimens he had; however, he indicated that he had both a male and a female specimen, and he also indicated two depositories (British Museum and Oxford).

The NHMUK houses a male type (NHMUK011250017), labeled (but not published) in 1968 as the lectotype by R. G. Beard, and subsequently designated as such by Penny (1982b: 431). Also, there is a female type in the OUMNH (NEUR0011, Fig. 57). It bears a “lectoallotype” label, also applied by R. G. Beard in 1968. It is a paralectotype.

**Current name.***Trichosceliairidella* (Westwood, 1867).

**Nomenclature.** Westwood’s subgenus Trichoscelia was given genus-level stature by Enderlein (1910: 376); the first use of the current combination probably was by Penny (1982b: 431). It was listed by Ohl (2004: 149) and Ardila-Camacho et al. (2018: 299, 306).

**Figure 58.**
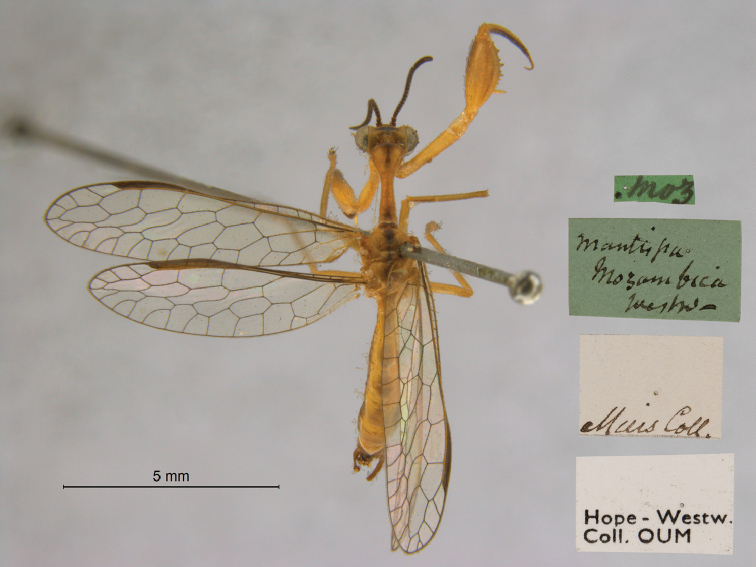
Holotype of *Mantispamozambica* Westwood, 1852 (NEUR0079, male).

**19. *mozambica* Westwood, 1852** (*Mantispa*) (Holotype; Fig. 58)

**Original description.***Trans. R. Entomol. Soc. Lond., 6 [1]: 269, Tab. 18, fig. 6*; “Habitat in Mozambica. In Mus. D. Miers.”. Sexes and number of specimens not specified.

**Type series.** Westwood’s description did not state specifically how many type specimens he had; the description of the abdomen indicates a male specimen. According to Ohl (2004: Footnote 36), the entire collection of D. Miers was transferred to the OUMNH; however, he did not report seeing the type of *M.mozambica*. Apparently, it was unnoticed for many years.

After a lengthy search, we found a single specimen, a male, with labels indicating that it is the type (NEUR0079, Fig. 58). Other types are unlikely to be found in the collection. Both Ohl (2004: 246) and Oswald (2018) indicate that holotype or syntype assignations are possible. We consider it a holotype.

**Current name.***Cercomantispamozambica* (Westwood, 1852).

**Nomenclature.** The current combination was proposed by Handschin (1959: 224) and listed by Ohl (2004: 164); the species is the type species of the genus *Cercomantispa* Handschin. Previously, the species also was known as Mantispa (Mantispilla) mozambica (Westwood) [combination by Enderlein (1910: 346)], *Mantispillamozambica* (Westwood) [combination by Navás (1914d: 40)], and *Necylamozambica* (Westwood) [source unknown, perhaps Navás 1915c: 178]. Apparently, the genera *Cercomantispa*, *Necyla*, and *Orientispa* have not been well delineated; additional study is needed (see Snyman et al. 2012).

**Figure 59.**
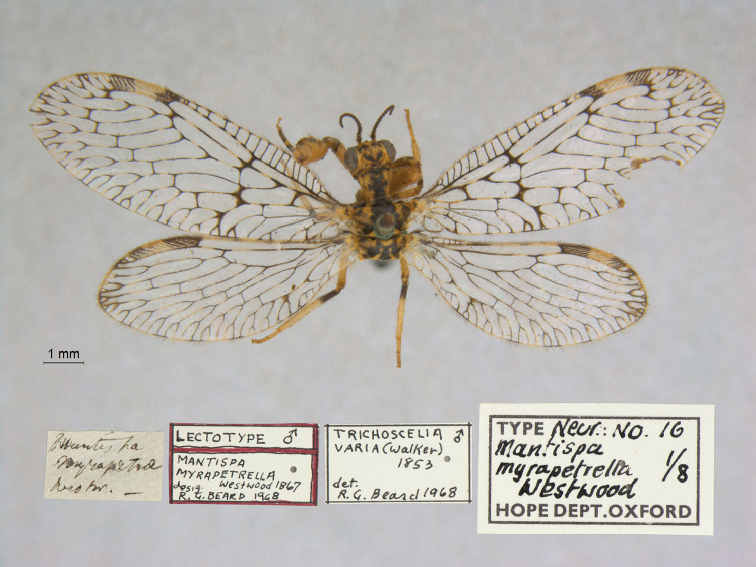
Lectotype (new designation) of *Mantispamyrapetrella* Westwood, 1867 (NEUR0016-01, male).

**Figure 60.**
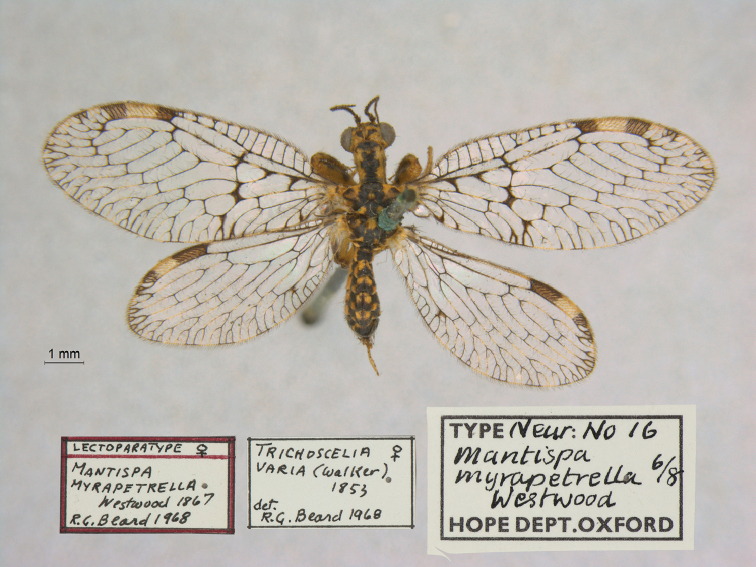
One of seven OUMNH paralectotypes for *Mantispamyrapetrella* Westwood, 1867 (NEUR0016-06, female).

**Figure 61.**
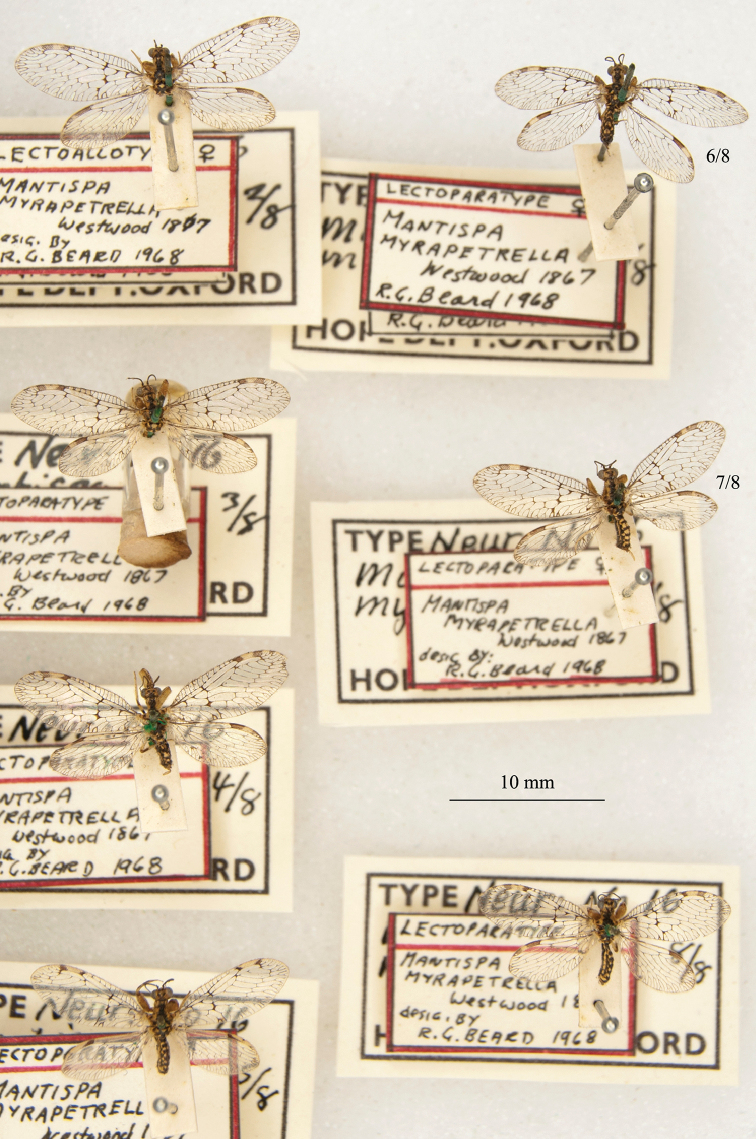
The seven OUMNH paralectotypes for *Mantispamyrapetrella* Westwood, 1867 (NEUR0016-02 through NEUR0016-08, two females, others unconfirmed).

**20. *myrapetrella* Westwood, 1867** (*Mantispa*) (Lectotype, new designation; seven paralectotypes; Figs 59, 60, 61)

**Original description.***Trans. R. Entomol. Soc. Lond., 15: 505*; “Habitat in nidis vespae [… in the nests of wasps] (*Myrapetraescutellaris*) Americae meridionalis [South America]. Vide White in Ann. Nat. Hist. vii. P. 322. In Muss. Brit. et Oxon.”. Sexes and number of specimens not specified.

**Type series.** Westwood’s description designated two depositories – NHMUK and OUMNH. Our less-than-exhaustive search did not find types of this species in the NHMUK.

Eight specimens were found in the OUMNH. One (a male, NEUR0016-01, Fig. 59) carries a lectotype label applied by R. G. Beard in 1968; however, this lectotype designation was not published. The other seven (NEUR0016-02 through NEUR0016-08; Figs 60, 61) carry Beard's paralectotype labels; two are females and the sexes of the others are not confirmed. Later, Penny (1982b: 436) referred to these specimens, but he did not identify a specific specimen to be the lectotype. In his research on mantispids, Penny usually followed the fine information on Beard’s labels. Thus, out of respect to both previous workers, here we designate the specimen in the OUMNH (NEUR0016-01) as the lectotype (present designation). The specimen carries no locality data, but it has Westwood’s, Beard’s, and the OUMNH’s identification labels. The abdomen of the specimen is held in glycerol in a genitalia vial on a separate pin.

The seven paralectotype specimens lack locality labels, but all have Hope Museum labels and Beard’s identification labels similar to those on the lectotype.

**Current name.***Trichosceliavaria* (Walker, 1853).

**Nomenclature.** The synonymy and current combination, *Trichosceliavaria* (Walker), were proposed by McLachlan (1868b: 261). Previously, the species was called *Symphrasismyrapetrella* (Westwood) [combination by Hagen (1877: 210)].

Unfortunately, the type locality, “Americae meridionalis” [South America], refers to a very large area, and neither the lectotype nor any of the paralectotypes carry locality labels. *Trichosceliavaria* is reported from Argentina and Brazil; early records of the species from Suriname and Venezuela are questioned (see Oswald 2018).

**Figure 62.**
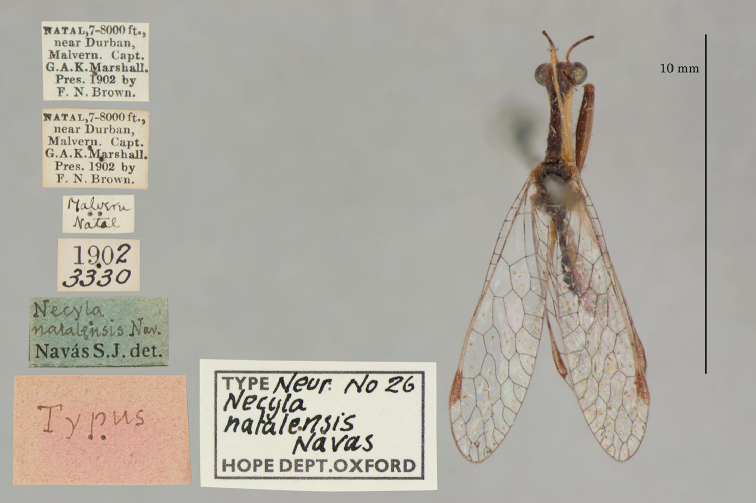
OUMNH syntype of *Necylanatalensis* Navás, 1914 (NEUR0026, sex undetermined).

**21. *natalensis* Navás, 1914** (*Necyla*) (Holotype/Syntype; Fig. 62)

**Original description.***Bol. Soc. Aragonesa Cien. Nat., 1914c, 13: 64*; “Africa meridional. ‘Natal, 7–800 ft., near Durban’, Mavern’ G. A. K. Marshall.”. Sexes and number of specimens not specified.

**Type series.** Navás’ article (by title) is focused on specimens in the OUMNH, and the description contained in the article gives measurements from one male specimen. The OUMNH holds only one specimen carrying Navás type labels for this species (NEUR0026, Fig. 62). It is unlikely that there are other types, and if this specimen were confirmed as a male, we would identify it as the holotype. However, the sex is now undetermined; so, in agreement with both Ohl (2004: 183) and Oswald (2018), it remains as a syntype, with a holotype assignment possible.

**Current name.***Afromantispanatalensis* Navás, 1914.

**Nomenclature.** The current combination was proposed by Snyman et al. (2015: 95).

**Figure 63.**
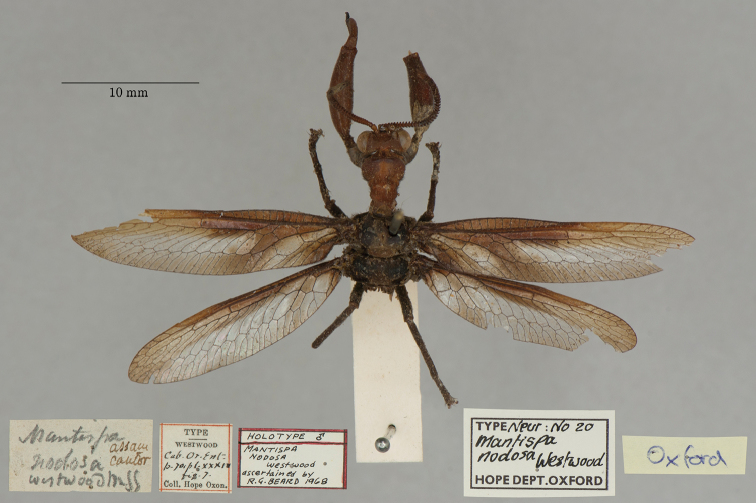
Holotype of *Mantispanodosa* Westwood, 1847 (NEUR0020, male).

**22. *nodosa* Westwood, 1847** (*Mantispa*) (Holotype; Fig. 63)

**Original description.***The Cabinet of Oriental Entomology; being a selection of the rarer and more beautiful species of insects, natives of India and the adjacent islands. The greater portion of which are now, for the first time, described and figured. Smith, London, 1848 [1847]: 70, fig. 7*; “Inhabits Assam. Dr. Cantor. In Mus. Hope.”. Sexes and number of specimens not specified.

**Type series.** Westwood indicated only the OUMNH as a depository, and the collection has only one type specimen. It is a male, with abdomen missing, that carries a label indicating the locality and collector as in the original description. It also has a label pointing out its association with the Cabinet of Oriental Entomology and a holotype label applied by R. G. Beard in 1968 (NEUR0020, Fig. 63). Ohl (2004: 172) reported that one or more syntypes may be present. Oswald (2018) called the specimen a holotype (by implicit monotypy), and as evidence of monotypy he cited a report by Shelford (1902: 236) that mentioned one type. At this time, we see no reason to question the holotype determination.

**Current name.***Euclimacianodosa* (Westwood, 1847).

**Nomenclature.**Ohl (2004: 172) proposed the current combination; it was subsequently cited by Bhattacharjee et al. (2010: 221–224).

**Figure 64.**
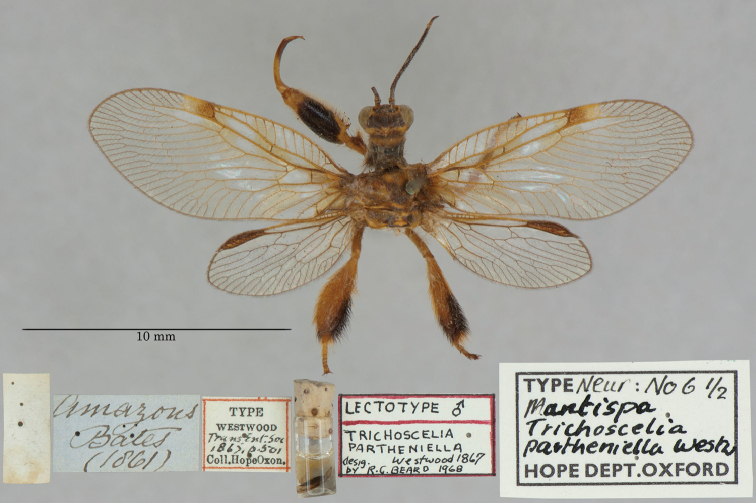
Lectotype of Mantispa (Trichoscelia) partheniella Westwood, 1867 (NEUR0006-01, male).

**Figure 65.**
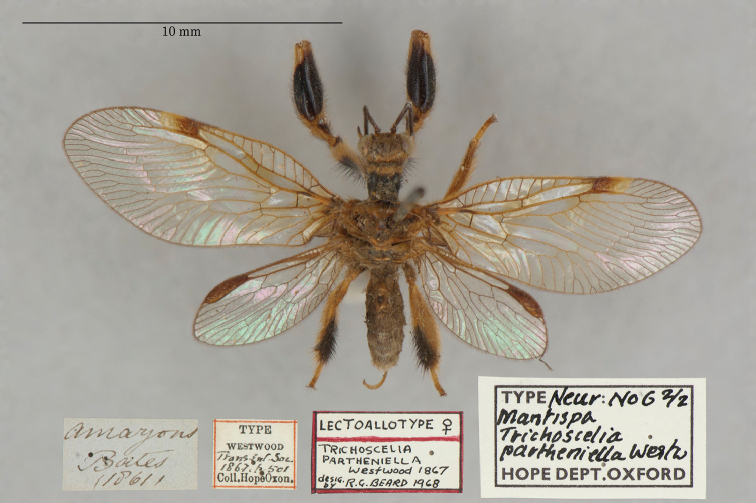
OUMNH paralectotype of Mantispa (Trichoscelia) partheniella Westwood, 1867 (NEUR0006-02, female).

**23. *partheniella* Westwood, 1867** [Mantispa (Trichoscelia)] (Lectotype, one paralectotype; Figs 64, 65)

**Original description.***Trans. R. Entomol. Soc. Lond., 15: 501*; “(Mas et foem.) [Male and female] … Habitat in Amazonia. D. Bates. In Mus. Oxon.”.

**Type series.** Although Westwood did not indicate how many type specimens he used, he did mention seeing both a male and a female specimen. Ohl (2004: 147) suggested the possibility of one or more syntypes.

Two specimens are found in the OUMNH: a male (NEUR0006-01, Fig. 64) with a lectotype label, and a female (NEUR0006-02, Fig. 65) with a paralectotype label. These type labels were applied by R. G. Beard in 1968, but he did not publish the information. However, Penny’s (1982b: 421) reference to the “Lectotype male and paralectotype female in the Hope Entomology Collection” served to fix the lectotype designation. Oswald (2018) listed the type as a lectotype (by explicit designation).

**Current name.***Anchietapartheniella* (Westwood, 1867).

**Nomenclature.** The current combination was proposed by Penny (1982b: 421) and listed by Ohl (2004: 147). Previously the species was known as *Trichosceliapartheniella* (Westwood) [generic assignment by Enderlein (1910: 376), but without immediate use of the combination; subsequent use of the combination by Penny (1982b: 421)]. *Anchietapartheniella* (Westwood) became the correct name when the name *Anisoptera* Schneider was identified as a junior homonym of *Anisoptera* Berthold, 1827, and its junior synonym *Anchieta* Navás, 1909 was recognized as the valid generic name (see Penny 1982a: 216–217).

**Figure 66.**
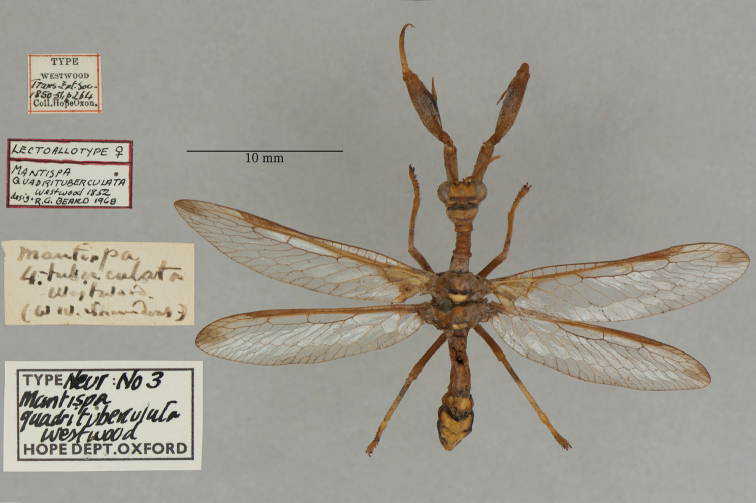
OUMNH paralectotype of *Mantispaquadrituberculata* Westwood, 1852 (NEUR0003, female).

**24. *quadrituberculata* Westwood, 1852** (*Mantispa*) (One paralectotype; Fig. 66)

**Original description.***Trans. R. Entomol. Soc. Lond., 6 [1]: 264, tab. 18, fig. 1*; “Habitat Northern India. Mus. W. W. Saunders.”. Sexes and number of specimens not specified.

**Type series.** Although Westwood did not state the number of specimens in the type series, he provided ranges for the morphological measurements that he made. Thus, clearly there was more than one specimen. Ohl (2004: 160) confirmed seeing a type in the OUMNH; he left open the question of additional syntypes. And, Oswald (2018) indicated that syntypes existed.

**Figure 67.**
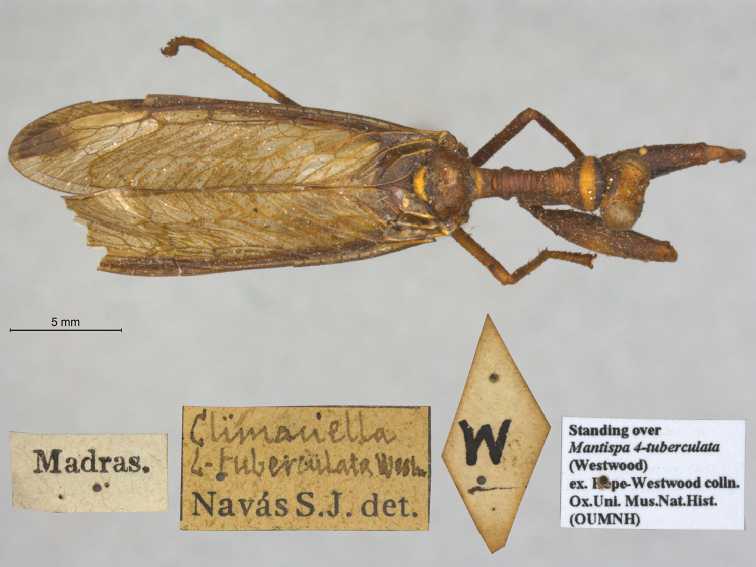
Historic, nontype specimen of *Mantispaquadrituberculata* Westwood, 1852. The specimen, sex undetermined, was studied by Westwood and Navás.

We identified two Westwood specimens in the OUMNH; the first one, a female, (NEUR0003, Fig. 66) was long considered to be an *M.quadrituberculata* syntype. R. G. Beard saw this specimen in 1986 and applied a “Lectoallotype ♀” label to it. He did not apply a similar label to the second specimen (Fig. 67), nor did Navás identify it as a type. These omissions imply that neither author considered the second specimen, sex undetermined, to be a type. Although it carries a typical diamond-shaped Westwood label, its locality data do not match those given in the original description of *M.quadrituberculata*. Thus, we also do not consider it part of the type series.

**New lectotype designation**, NHMUK specimen.The NHMUK has another type for this species (NHMUK011250015); its labels also confirm it as a syntype. The specimen is on a pin, and the left wings are mounted on a slide with a holotype label (NHMUK010110641). It is likely that Beard saw this specimen; however, surprisingly, it does not carry his lectotype label. We assume from Beard’s “Lectotallotype” label on the OUMNH specimen that he regarded the NHMUK specimen as the lectotype, but hesitated to identify it as such perhaps because of the holotype label on the slide. Given our confidence that the specimen in the OUMNH indeed was part of the type series, here we designate the specimen in the NHMUK as the lectotype (present designation). Its labels read: (1) “Type”, (2) not clear, (3) “Saunders 68-3”, (4) “Mantispa 4-tuberculata Westw.”, (5) “Wings in slide cabinet”, (6) “NHMUK011250015”. And, we confirm Beard’s identification of the OUMNH specimen as the paralectotype.

**Current name.***Austroclimaciellaquadrituberculata* (Westwood, 1852).

**Nomenclature.** The current combination was proposed by Handschin (1961: 287) and listed by Ohl (2004: 160). Previously, the species was known by a number of names: *Eumantispaquadrituberculata* (Westwood) [combination by Banks (1931: 386); combination used, but not cited as new]; *Ditaxisquadrituberculata* (Westwood) [combination by McLachlan (1868b: 262)]; *Climaciellaquadrituberculata* (Westwood) [combination by Enderlein (1910: 361)].

In the original description, the species name was given as “*4-tuberculata*”; this spelling was changed in compliance with ICZN Article 32.5.2.6.

**Figure 68.**
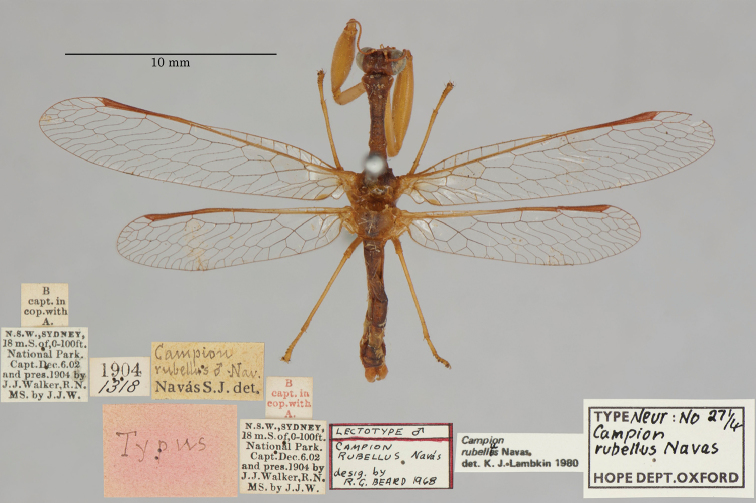
Lectotype of *Campionrubellus* Navás, 1914 (NEUR0027-01, male).

**Figure 69.**
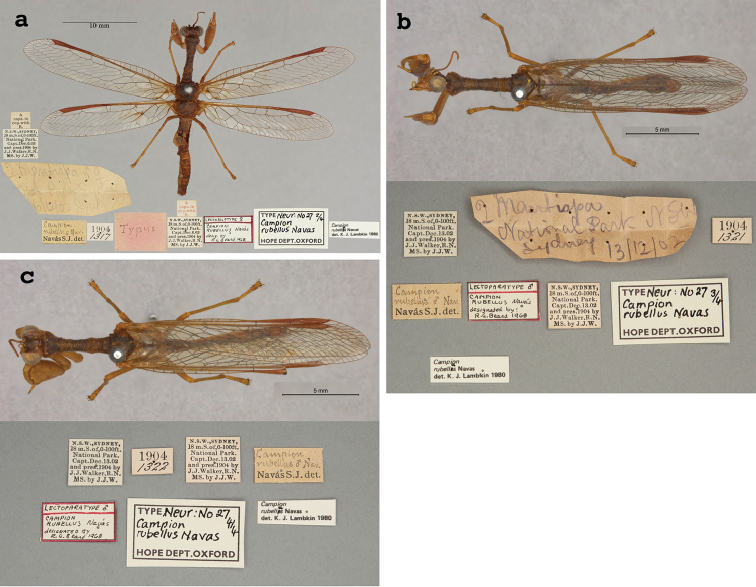
Three OUMNH paralectotypes of *Campionrubellus* Navás, 1914 **a** NEUR0027-02, female **b** NEUR0027-03, male **c** NEUR0027-04, male.

**25. *rubellus* Navás, 1914** (*Campion*) (Lectotype, three paralectotypes; Figs 68, 69)

**Original description.***Bol. Soc. Aragonesa Cien. Nat., 1914c, 13: 65, fig. 1*; “Australia: ‘N. S. W. Sydney. 18 m. S. of 0–100 ft., National Park’ 6 Diciembre 1902, J. J. Walker. He visto otro ejemplar ♂ de Australia del Museo de Londres.”. [The locality listed is probably near Audley in what is now called Australia’s Royal National Park. Established in 1870, it is Australia’s first national park, and one of the first in the world (information from Graham Owen, NSW)].

**Type series.** Navás’ article, as stated in the title, is focused on specimens in the OUMNH, and from his original description it is clear that he studied at least one male and one female specimen of *C.rubellus* there. He also mentioned a male specimen from Australia in the NHMUK. He did not designate a primary type.

In the OUMNH collection, there are four Navás specimens, two males and two females (Figs 68, 69). All four bear Navás’ determination labels; a male and a female also carry Navás’ “Typus” labels. In 1968, R. G. Beard applied his lectotype label to the male specimen in the OUMNH that carries a Navás “Typus” label (NEUR0027-01, Fig. 68), but he did not publish this designation. Later, Lambkin (1986b: 14) designated Beard’s specimen as the lectotype. The three other Navás syntypes in the OUMNH (female, NEUR0027-02, Fig. 69a; males, NEUR0027-03, -04, Fig. 69b, c) carry Beard’s paralectotype labels and are recognized as such (see New 1996: 30, Oswald 2018). Apparently, Ohl (2004: 163) erred in reporting the types; he reported male and female types from the NHMUK, but none from the OUMNH. Any NHMUK syntypes would be paralectotypes.

**Current name.***Campionrubellus* Navás, 1914.

**Nomenclature.** The name has remained unchanged; it was listed by Ohl (2004: 163).

**Figure 70.**
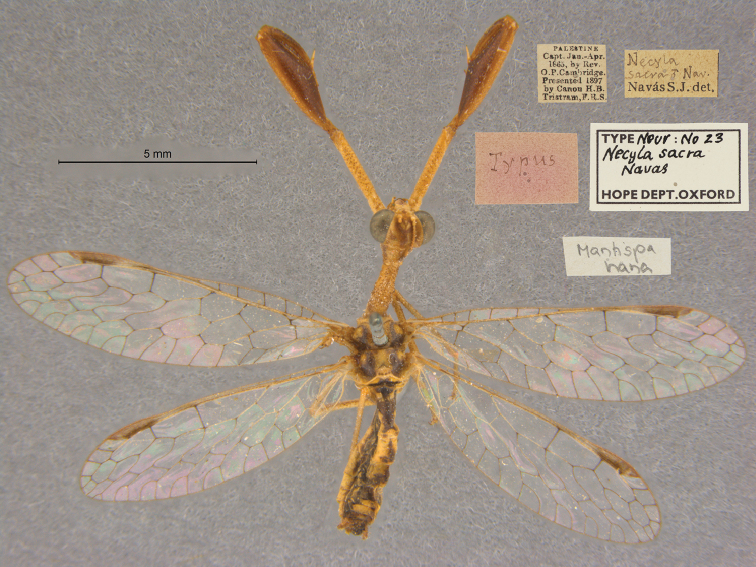
Holotype of *Necylasacra* Navás, 1914 (NEUR0023, male).

**26. *sacra* Navás, 1914** (*Necyla*) (Holotype; Fig. 70)

**Original description.***Bol. Soc. Aragonesa Cien. Nat., 1914c, 13: 63*; “Palestina. Rev. O. P. Cambridge [Rev. O. Pickard-Cambridge], 1865.”. Number of specimens not specified.

**Type series.** Navás’ article, as stated in the title, is focused on specimens from the OUMNH. In it, Navás gave measurements for one male *N.sacra* specimen, and he made no mention of other specimens. The collection in the OUMNH has one type specimen, a male with Navás’ labels (NEUR0023, Fig. 70). Both Ohl (2004: 194) and Oswald (2018) indicate that a syntype or a holotype assignation is possible. However, to be consistent with other mantispid species treated here (see explanation under *M.fasciatella*, above) this specimen should be considered the holotype (by implicit monotypy).

**Current name.***Necylasacra* Navás, 1914.

**Nomenclature.** The name has remained unchanged; it was listed by Ohl (2004: 194).

**Figure 71.**
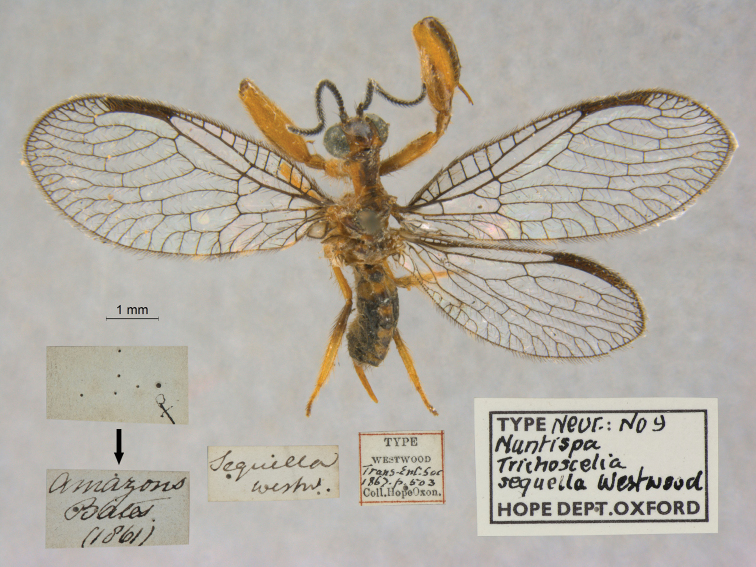
Holotype of Mantispa (Trichoscelia) sequella Westwood, 1867 (NEUR0009, female).

**27. *sequella* Westwood, 1867** [Mantispa (Trichoscelia)] (Holotype; Fig. 71)

**Original description.***Trans. R. Entomol. Soc. Lond., 15: 503*; “(Foem.) [Female] … Habitat in Amazonia. D. Bates. In Mus. Oxon.”.

**Type series.** In the original description, Westwood specified only one depository, the OUMNH, and he mentioned only one specimen, a female (NEUR0009, Fig. 71). This specimen is in the collection. Penny (1982b: 435) determined it to be the holotype; subsequently Ohl (2004: 150) listed it as such. Oswald (2018) listed it as the holotype (by explicit monotypy).

**Current name.***Trichosceliasequella* (Westwood, 1867).

**Nomenclature.** The combination was proposed by Penny (1982b: 435) and subsequently listed by Ohl (2004: 150). The species was previously known as *Trichosceliasequella* Westwood [generic assignment by Enderlein (1910: 376), but without use of the combination] and *Anisopterasequella* (Westwood) [generic assignment by Gerstaecker (1887: 117), but without use of the combination].

**Figure 72.**
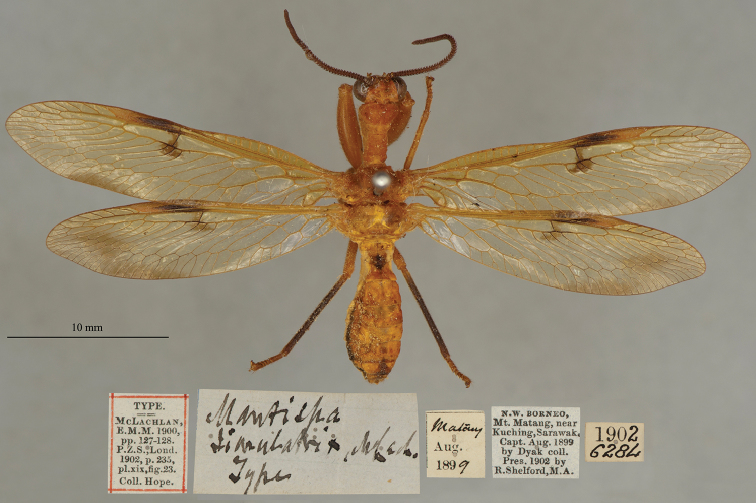
Holotype of *Mantispasimulatrix* McLachlan, 1900 (NEUR0071, female).

**28. *simulatrix* McLachlan, 1900** (*Mantispa*) (Holotype; Fig. 72)

**Original description.***Entomol. Monthly Mag., 36: 127–129, fig. unnumbered*; “Matang, Borneo, August, 1899. One ♀. The type may be seen for the present in the Hope Collection, …. It will ultimately be deposited in the Sarawak Museum, to which it belongs.”.

**Type series.** McLachlan’s original description stated that there was a single type (a female) and that it would be transferred to the Sarawak Museum. However, as reported by Ohl (2004: Footnote 90) and as we confirm here, the specimen (NEUR0071, Fig. 72; holotype, by original designation) remains in the OUMNH. The image (Fig. 72) and its label data clearly match those in the original description. The species’ mimicry was discussed, and the adult was illustrated by Shelford (1902: 235, Plate XIX, fig. 23), who originally provided McLachlan with the specimen.

**Current name.***Mimetispasimulatrix* (McLachlan, 1900).

**Nomenclature.** This species is now the sole member of the genus *Mimetispa* Handschin; the current combination was proposed by Handschin (1961: 276–7) and listed by Ohl (2004: 192). The species also was previously referred to as *Euclimaciasimulatrix* (McLachlan) by Banks (1931: 386), without comment. See Handschin (1961: 277) for a full synonymy.

**Figure 73.**
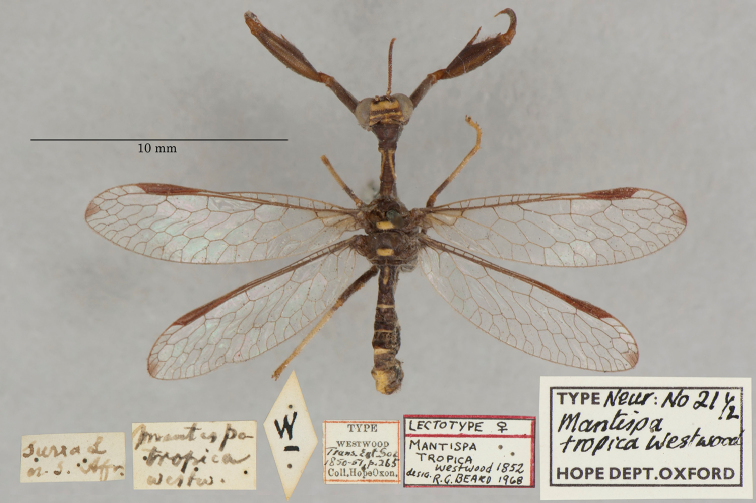
Lectotype (new designation) of *Mantispatropica* (*Mantispa*) Westwood, 1852 (NEUR0021-01, female).

**Figure 74.**
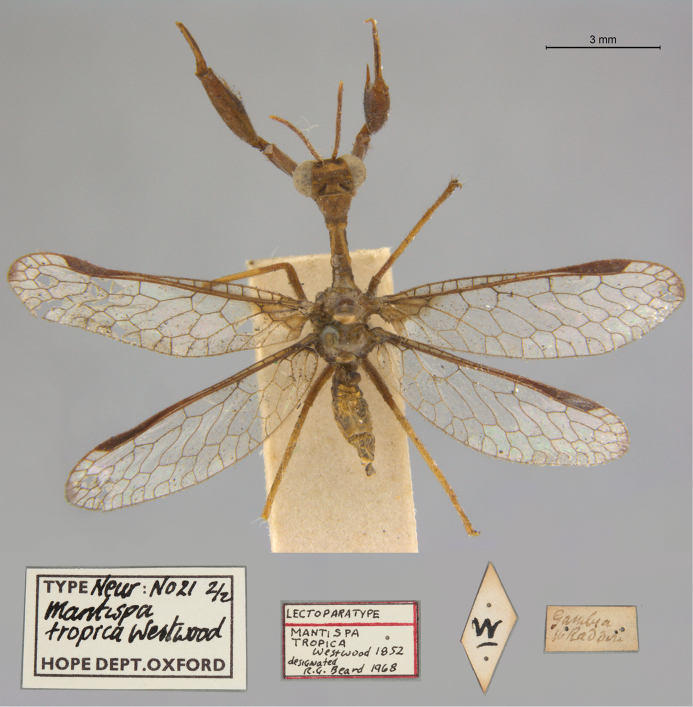
OUMNH paralectotype of *Mantispatropica* Westwood, 1852 (NEUR0021-02, sex undetermined).

**29. *tropica* Westwood, 1852** (*Mantispa*) (Lectotype, new designation; one paralectotype; Figs 73, 74)

**Original description.***Trans. R. Entomol. Soc. Lond., 6 [1]: 265, tab. 18, fig. 2*; “Habitat Africa tropicali occidentali, Gambia. Mus. Westwood.”. Sexes and number of specimens not specified.

**Type series.**Ohl (2004: 201) stated that there was one or more syntypes. Two syntypes are in the collection: One, a female (NEUR0021-01, Fig. 73), carries a lectotype label that was applied by R. G. Beard in 1968. The other (NEUR0021-02, Fig. 74, sex unknown) carries a paralectotype label. These designations were not published. Thus, here we confirm Beard’s choice of the female NEUR0021-01 as the lectotype (present designation) of *Mantispatropica* Westwood. The second specimen becomes a paralectotype as labeled.

**Current name.***Pseudoclimaciellatropica* (Westwood, 1852).

**Nomenclature.** The current combination was proposed by Handschin (1960a: 209, 227) and listed by Ohl (2004: 201). Oswald (2018) also lists the species name in two additional combinations: *Mantispillatropica* (Westwood) and *Necylatropica* (Westwood). Both of these combinations were used without comment by Handschin (1959: 187); they were not listed by Ohl (2004: 199). Note: When Enderlein (1910: 355) described subgenera for *Mantispa*, he explicitly assigned *M.tropica* to subgenus Mantispa, not *Mantispilla*.

#### Myrmeleontidae (Antlions, doodlebugs)

The Myrmeleontidae is the largest and perhaps best-known family of Neuroptera. It contains ~1600 extant species in slightly more than 200 genera, which traditionally have been grouped into three subfamilies. This classification may soon change when recent genetic data are taken into account (Winterton et al. 2018). The larvae are generally considered to be “sit and wait” predators that inhabitat bark, tree holes, debris, or soil. Some species build pit traps in sand to capture their prey. Adults are usually nocturnal, and their flight resembles that of damselflies.

The OUMNH is reported to hold types of 22 myrmeleontid species; we found types for 20, including 16 species with primary types (holotype, syntype, or lectotype) and four species with secondary types only (paratypes or paralectotypes). Types could not be located for two species; both were described by Navás in 1913, but in separate publications. Of the species with primary types in the OUMNH, almost half are syntypes, and there are more holotypes (n = 6) than lectotypes (n = 3). The species with OUMNH types were described by several systematists: nine by Navás, nine by Rambur, two by Kimmins, one by Esben-Petersen, and another by Westwood. Most are from Africa, the East Indies, and Oceania; only one is from the New World. In total, there are 26 type specimens of Myrmeleontidae in the collection.

In his comprehensive catalog of the world’s antlions, L. A. Stange provided full taxonomic information for the species described prior to 2001 (Stange 2004). Thus, for most species here, our section on “Nomenclature” provides only the current valid name, the original reference for the source of the current name, and a reference (page number) for the species’ full synonymy in Stange’s catalog. To search for name changes that occurred after Stange’s catalog was published, we used the online Neuroptera catalog (Oswald 2018) and also conducted our own review of recent literature.

**Figure 75.**
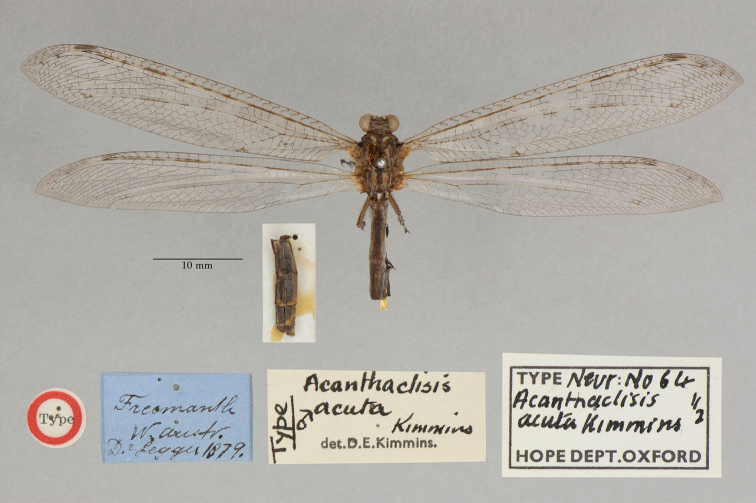
Holotype of *Acanthaclisisacuta* Kimmins, 1939 (NEUR0064-01, male).

**Figure 76.**
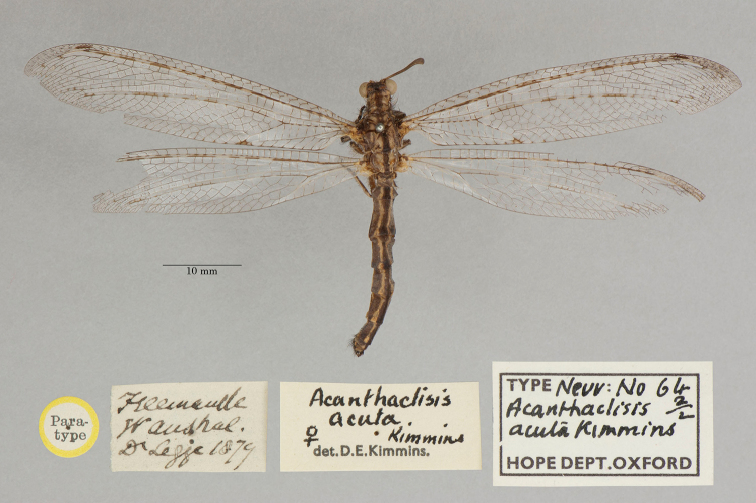
OUMNH paratype of *Acanthaclisisacuta* Kimmins, 1939 (NEUR0064-02, female).

**1. *acuta* Kimmins, 1939** (*Acanthaclisis*) (Holotype, one paratype; Figs 75, 76)

**Original description.***Ann. Mag. Nat. Hist., 1939: 588, Plate 18, fig. b.* “W. AUSTRALIA: Freemantle, 1879 (*Dr.* Legge), 1♂, 1♀. Type ♂ in the Hope Department, University Museum, Oxford.”.

**Type series.**Kimmins (1939: 588) indicated that there were only two specimens in the type series, and he identified the depository of the male, not the female. We assume that with this action, he intended to designate the male as the primary name-bearing specimen. Thus, in agreement with Stange (2004: 352) and Oswald (2018), we consider the specimens in the OUMNH to be a male holotype (by original designation) and a female paratype (NEUR0064-01, -02; Figs 75, 76).

**Current name.***Heoclisisacuta* (Kimmins, 1939).

**Nomenclature.** The current generic assignment was made by New (1985: 57); see Stange (2004: 352).

**Figure 77.**
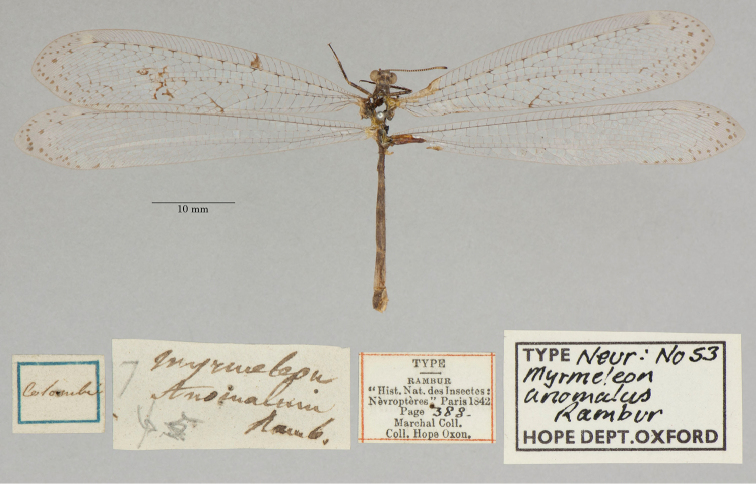
Lectotype (new designation) of *Myrmeleonanomalus* Rambur, 1842 (NEUR0053, sex undetermined).

**2. *anomalus* Rambur, 1842** (*Myrmeleon*) (Lectotype, new designation; Fig. 77)

**Original description.***Libr. encycl. Roret, 1842: 388.* “De la Colombie; collection de M. Marchal.”. Sexes and number of specimens not specified.

**Type series.**Stange (2004: 168) reported that the type(s) had not been located. Although there are specimens of this species in the NHMUK, none appears to be a type. However, there is one specimen labeled as a type (sex undetermined) in the OUMNH (NEUR0053, Fig. 77). To help stabilize the nomenclature associated with this species, and because of the clear identity of the OUMNH specimen (NEUR0053) as Rambur’s type, here we designate it as the lectotype (present designation).

**Current name.***Eremoleonanomalus* (Rambur, 1842).

**Nomenclature.** The current generic assignment was by Stange (1967: 57); also see Stange (2004: 168).

**Figure 78.**
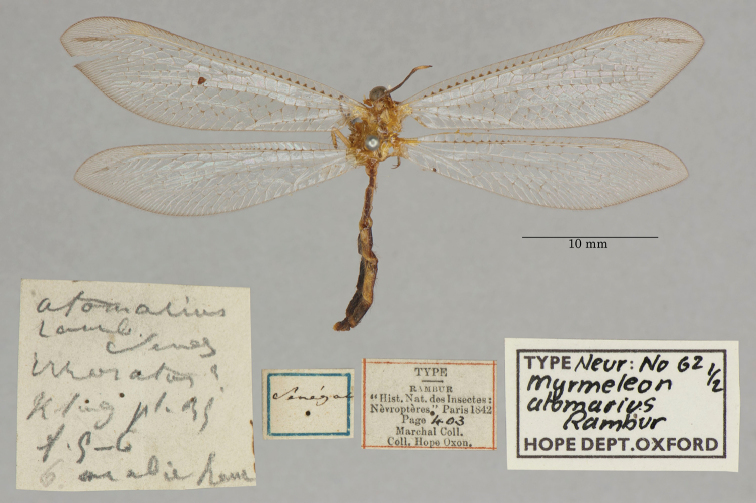
First of two OUMNH syntypes for *Myrmeleonatomarius* Rambur, 1842 (NEUR0062-01, probably female).

**Figure 79.**
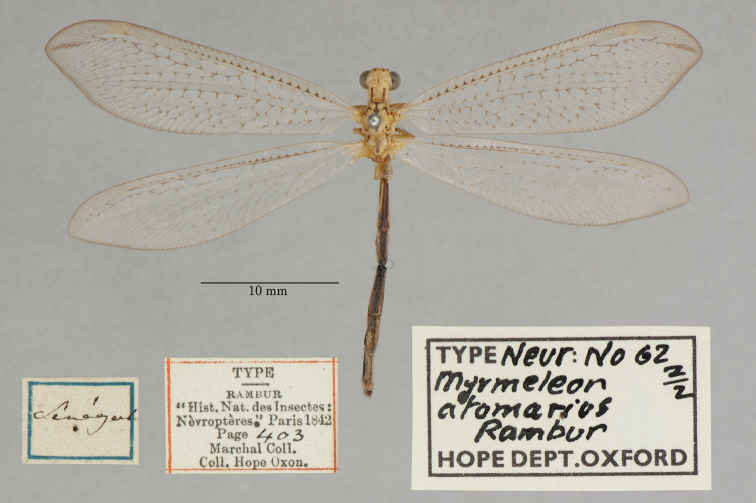
Second of two OUMNH syntypes for *Myrmeleonatomarius* Rambur, 1842 (NEUR0062-02, sex undetermined).

**3. *atomarius* Rambur, 1842** (*Myrmeleon*) (Two syntypes; Figs 78, 79)

**Original description.***Libr. encycl. Roret, 1842: 399.* “Habite le Sénégal.”. Sexes and number of specimens not specified.

**Type series.** The original description mentions both male and female features; thus it is clear that the type series contained more than one specimen. Later, McLachlan (1873b: 136) mentioned seeing a female specimen in the Marchal collection at Oxford; he did not see a male. Currently, there are two Rambur syntypes in the OUMNH. Both are labeled as types, both are from Senegal, and both were in the Marchal collection. One (NEUR0062-01, Fig. 78) probably is a female; the sex of the other (NEUR0062-02, Fig. 79) is undetermined.

A third syntype of this species is in the NHMUK (NHMUK010288325). It is missing its abdomen, and it is listed under its current genus name, *Myrmecaelurus*. Also, Stange (2004: 267) reported a holotype female in the IRSNB. In the absence of specific information on the specimen in the IRSNB, Stange’s report does not constitute a valid lectotype designation. Thus, syntypes of this species are reported from the OUMNH, NHMUK, and IRSNB.

**Current name.***Myrmecaelurusatomarius* (Rambur, 1842).

**Nomenclature.** The current generic assignment was proposed by Banks (1913b: 154); see Stange (2004: 267).

**Figure 80.**
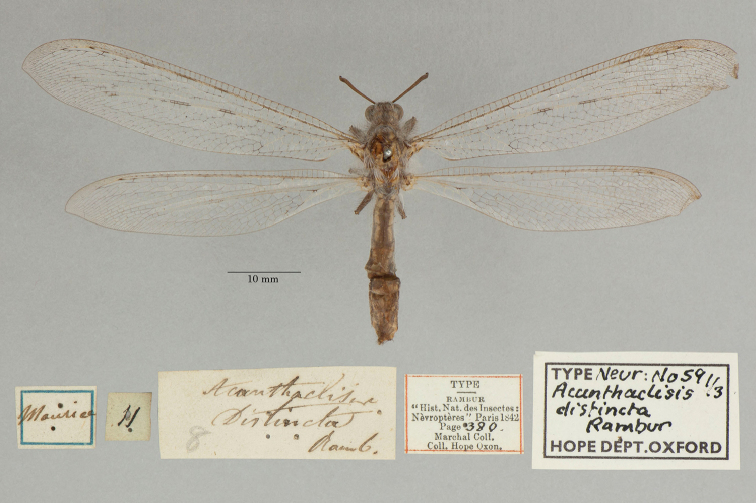
One of three OUMNH paralectotypes for *Acanthaclisisdistincta* Rambur, 1842 (NEUR0059-01, female). Note: This specimen appears to be Rambur’s female specimen from Mauritius.

**Figure 81.**
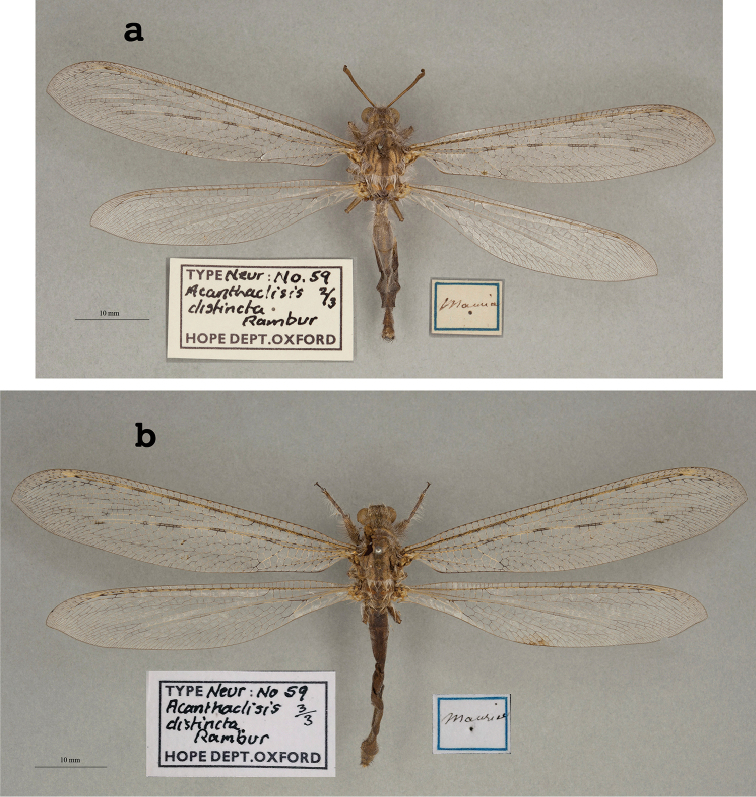
Two of three OUMNH paralectotypes for *Acanthaclisisdistincta* Rambur, 1842 **a** NEUR0059-02, sex undetermined **b** NEUR0059-03, sex undetermined.

**4. *distincta* Rambur, 1842** (*Acanthaclisis*) (Three paralectotypes; Figs 80, 81)

**Original description.***Libr. encycl. Roret, 1842: 380.* “Du Sénégal. M. Marchal m’a communiqué une femelle de Maurice, dont la teinte générale est .... [From Senegal. Mr. Marchal has given me a female from Mauritius, whose general color is ….]”.

**Type series.**Prost (1998: 162) studied Rambur’s specimens in the IRSNB, and from them he designated a lectotype: a male from Senegal with a damaged abdomen, in the “coll. Sélys”. It carried a red determination label in Rambur’s handwriting. He also noted that he had not found the female specimen from the Isle of Maurice (Mauritius) that Rambur mentioned. Based on the original description, he could not determine whether Rambur included this second Mauritian specimen in the type series, but he was certain that it did not belong to the same species as the other specimens.

Stange (2004: 344) confirmed Prost’s report and stated that he had examined the male lectotype in the IRSNB, probably in 1964; he did not mention the female specimen from Mauritius.

Three specimens are in the OUMNH. All three bear a label reading “Maurice”, and one of these, a female, bears an identification label in Rambur’s handwriting. This specimen appears to be the female that Rambur specifically mentioned in his description; it certainly is a paralectotype (NEUR0059-01, Fig. 80). The other two specimens are very similar in all respects; they also appear to have been part of the type series. We consider them to be paralectotypes (NEUR0059-02, -03; Fig. 81a, b). Their sexes and species identities are undetermined. In addition, four specimens of this species studied by McLachlan are in the NHMUK. Whether Rambur also studied them is unknown at this time.

**Current name.***Centroclisisdistincta* (Rambur, 1842).

**Nomenclature.** The genus name was reassigned by Prost (1998: 164); see Stange (2004: 344).

**Figure 82.**
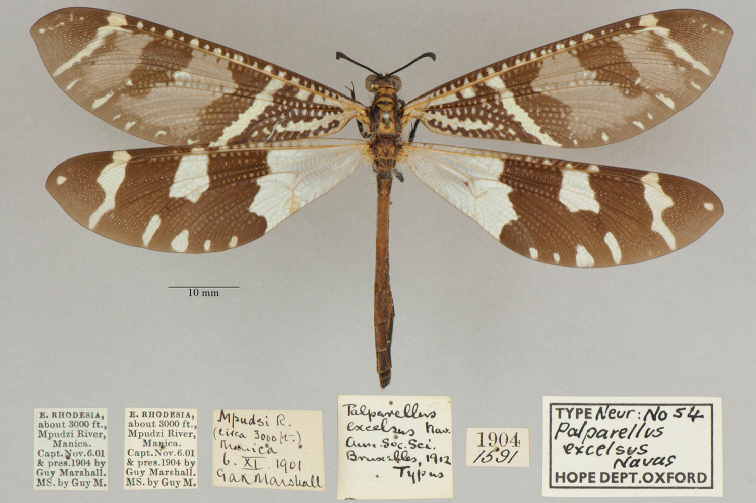
Holotype of *Palparellusexcelsus* Navás, 1913 (NEUR0054, female).

**5. *excelsus* Navás, 1913** (*Palparellus*) (Holotype; Fig. 82)

**Original description.***Ann. Soc. sci. Bruxelles, 1913b, 37 (pt. 1): 89, fig. 2.* “Afrique. Un échantillon au musée d’Oxford étiqueté: E. Rhodesia about 3000 ft., Mpudri [Sic!] River, Manica, Capt. Nov.6.01, Guy Marshall.”.

**Type series.** Navás stated that he had one specimen, and in the text he referred only to a female. We conclude that the sole type in the OUMNH, a female (NEUR0054, Fig. 82), is the holotype (by explicit monotypy) (in agreement with Stange 2004: 45, Oswald 2018). It was collected in 1901 and presented to the Museum in 1904 by Guy Marshall.

**Current name.***Palparellusnyassanus* (Navás, 1911).

**Nomenclature.***Palparellusexcelsus* was synonymized under *P.nyassanus* by Mansell (1996: 243); see Stange (2004: 45).

**Figure 83.**
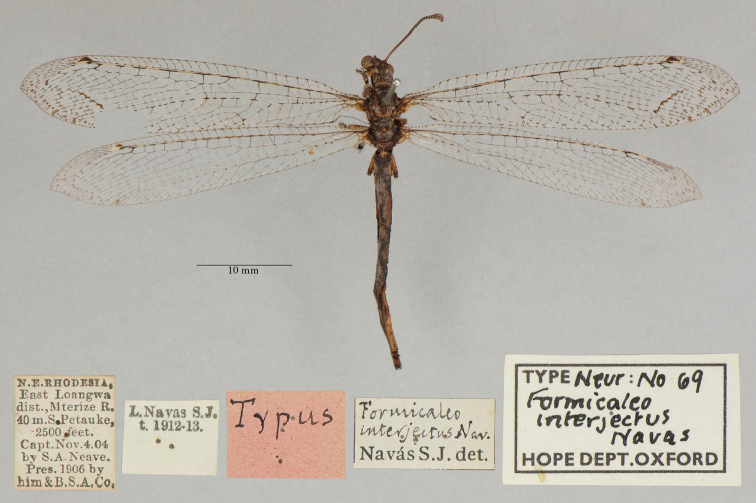
Holotype of *Formicaleointerjectus* Navás, 1913 (NEUR0069, female).

**6. *interjectus* Navás, 1913** (*Formicaleo*) (Holotype; Fig. 83)

**Original description.***Mem. R. Acad. Cien. Artes Barcelona, 1913a, (3) 10: 492 (fig. 6).* “Un ejemplar lleva esta rótulo: N. E. Rhodesia, East Loangwa dist. Mterize R. 40 m. S. Petauke, 2500 feet. Capt. Nov. 4. 04 by S. A. Neave.”.

**Type series.** Navás mentioned one specimen; he also referred to a female in the text of his description.

One type is in the OUMNH (NEUR0069, Fig. 83); it appears to be a female, and it carries a determination label and a Navás “Typus” label. It is the holotype (by explicit monotypy).

**Current name.***Distoleoninterjectus* (Navás, 1913).

**Nomenclature.** The current combination is by Stange (2014: 155).

**7. *isopterus* Navás, 1913** (*Gymnoleon*) (Holotype, not found)

**Original description.***Mem. R. Acad. Cien. Artes Barcelona, 1913a, (3) 10: 489* “Africa meridional. El rótulo dice: N. W. Rhodesia, Alala Plateau, Mkushi distr., about 4000 ft. 19-IX-1905. S. A. Neave. Un ejemplar (Mus. de Oxford).”.

**Type series.** Navás reported that he examined one specimen and that it was in the OUMNH. Stange (2004: 181) listed it as being in the OUMNH, but he did not report seeing a type. Thus far, we have been unable to find the specimen in the collection. If found, it would be a holotype (by explicit monotypy).

**Current name.***Gymnoleonisopterus* Navás, 1913.

**Nomenclature.** The name has remained unchanged; see Stange 2004: 181.

**Figure 84.**
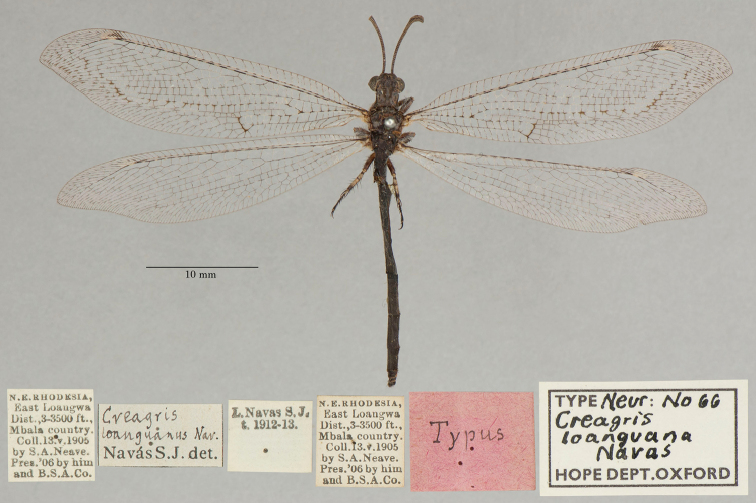
OUMNH syntype of *Creagrisloanguana* Navás, 1913 (NEUR0066, sex undetermined).

**8. *loanguana* Navás, 1913** (*Creagris*) (Syntype; Fig. 84)

**Original description.***Mem. R. Acad. Cien. Artes Barcelona, 1913a, (3) 10: 489.* “N. E. Rhodesia, East Loangwa, Dist. 3–3500 ft., Mbala country, Coll. 13-V-1905, S. A. Neave (Mus. de Oxford).”. Sexes and number of specimens not specified.

**Type series.** Navás did not indicate how many specimens he examined. A single type, sex undetermined, is in the OUMNH (NEUR0066, Fig. 84). In the absence of information on the number of specimens Navás studied, we consider this specimen to be a syntype. No lectotype has been designated.

**Current name.***Creoleonloanguanus* (Navás, 1913).

**Nomenclature.** The current combination was proposed by Stange (2004: 139, as *loanguana*).

**Figure 85.**
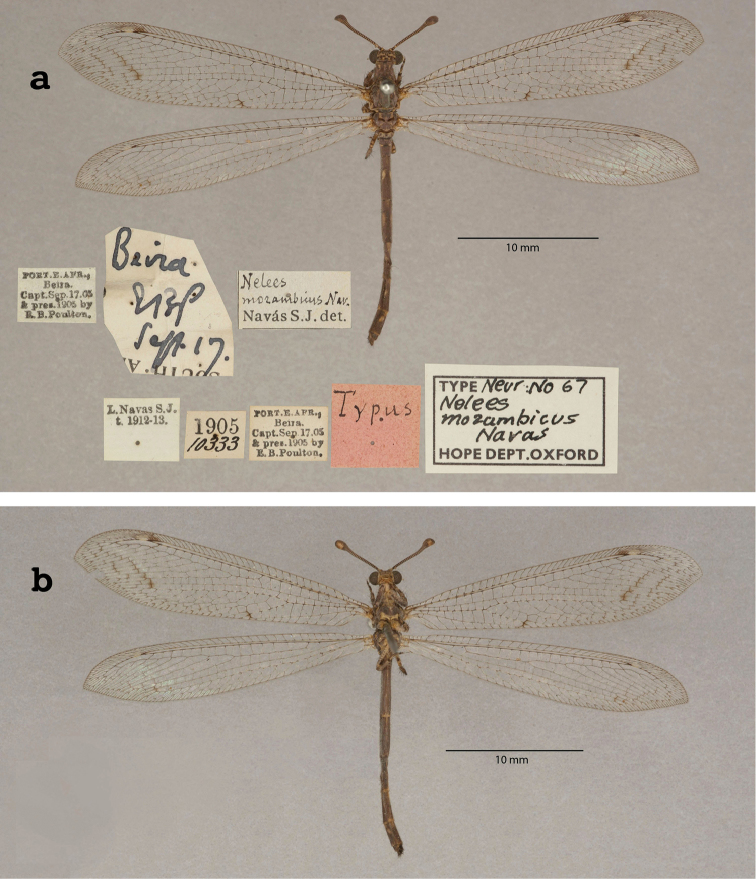
OUMNH syntype of *Neleesmozambicus* Navás, 1913 (NEUR0067, female) **a** dorsal **b** ventral.

**9. *mozambicus* Navás, 1913** (*Nelees*) (One syntype; Fig. 85)

**Original description.***Mem. R. Acad. Cien. Artes Barcelona, 1913a, (3) 10: 490.* “Africa Oriental portuguesa. Beira, 17 Septiembre de 1905.”. Sexes and number of specimens not specified.

**Type series.** One type, a female, is in the OUMNH (NEUR0067, Fig. 85a, b). Because there is no evidence that this was Navás’ only specimen, we consider it a syntype. No lectotype has been designated.

**Current name.***Neuroleonmozambicus* (Navás, 1913).

**Nomenclature.** The current combination was offered by Stange (2004: 207); it is listed as Neuroleon (Neuroleon) mozambicus (Navás, 1913) by Oswald (2018).

**Figure 86.**
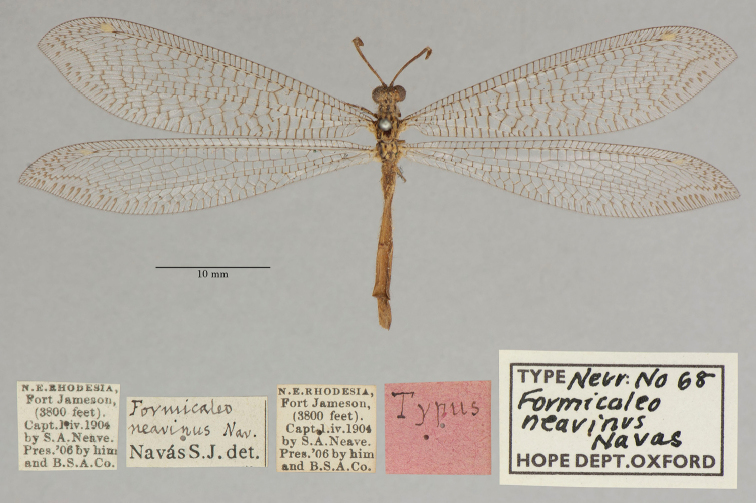
Lectotype (new designation) of *Formicaleoneavinus* Navás, 1913 (NEUR0068, sex undetermined).

**10. *neavinus* Navás, 1913** (*Formicaleo*) (Lectotype; Fig. 86)

**Original description.***Mem. R. Acad. Cien. Artes Barcelona, 1913a, (3) 10: 491.* “N. E. Rhodesia, Fort Jameson 3800 ft, 1-IV-1904, S. A. Neave (Mus. de Oxford).”. Sexes and number of specimens not specified.

**Type series.** Only one depository (OUMNH) is mentioned for this species, and only one specimen, sex undetermined, is present (NEUR0068, Fig. 86). We could find no other evidence for monotypy either in the original description or elsewhere. Thus, contrary to Stange (2004: 157) who identified the specimen as the holotype, and consistent with the manner that we have treated other specimens here, we consider the specimen to be a syntype. Stange’s identification of the specimen as the holotype fixes it as the lectotype (ICZN Article 74.7).

**Current name.***Distoleonneavinus* (Navás, 1913).

**Nomenclature.** The current combination was proposed by Stange (2004: 157).

**Figure 87.**
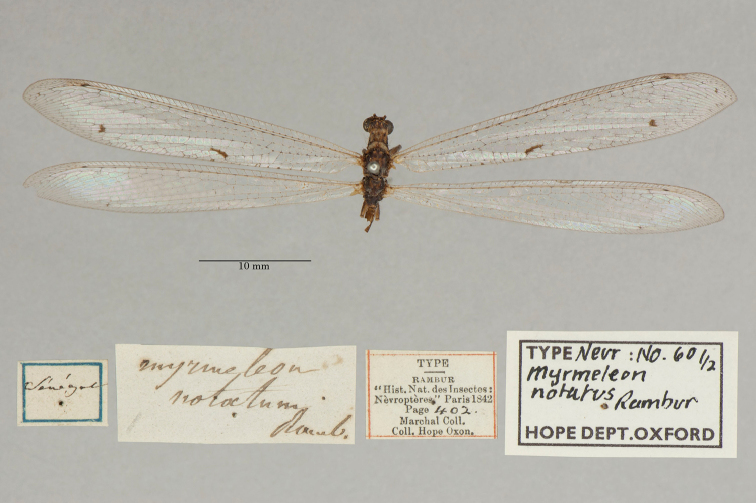
First of two OUMNH syntypes for *Myrmeleonnotatus* Rambur, 1842 (NEUR0060-01, sex undetermined).

**Figure 88.**
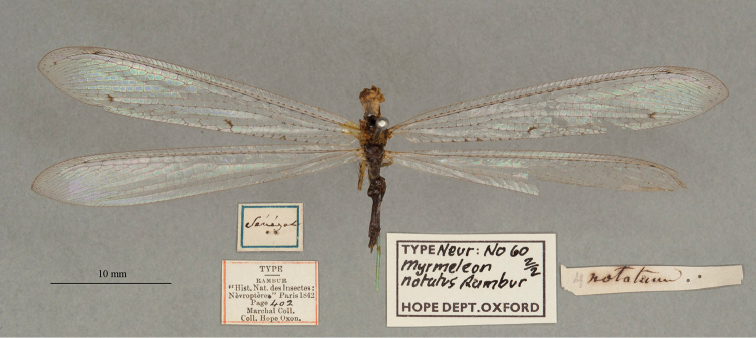
Second of two OUMNH syntypes for *Myrmeleonnotatus* Rambur, 1842 (NEUR0060-02, sex undetermined).

**11. *notatus* Rambur, 1842** (*Myrmeleon*) (Two syntypes; Figs 87, 88)

**Original description.***Libr. encycl. Roret, 1842: 402.* “Je l’ai pris dans les environs de Malaga, et M. Marchal me l’a communiqué du Sénégal.”.

**Type series.** Rambur mentioned two localities (Senegal and Málaga), but whether specimens were collected from both is unclear. McLachlan (1873b: 137) reported seeing three types: “J’en ai vu trois types, dont l’un m’a été envoyé par M. de Sélys, et ne porte point d’étiquette de la localité (probablement de Malaga), les autres sont dans la collection Marchal, et sont indiqués comme du Sénégal. [I saw three types, one of which was sent to me by M. de Sélys and bears no locality label (probably from Málaga), the others are in the Marchal collection and identified as from Senegal.]”. Thus, it appears that the description was based on specimens from both Senegal and Málaga.

The OUMNH holds the two syntypes (sexes unconfirmed) of this species that McLachlan reported from the Marchal Collection (NEUR0060-01, -02; Figs 87, 88). Both carry labels reading “Senegal”, and both bear identification labels in Rambur’s handwriting. We did not find the third type. Several specimens of this species are in the NHMUK, but none are identified as types.

We know of no published lectotype designations or holotype identifications. At this time, we consider the two OUMNH specimens to be syntypes.

**Current name.***Nemoleonnotatus* (Rambur, 1842).

**Nomenclature.** The current combination was offered by Stange (2004: 118).

**Figure 89.**
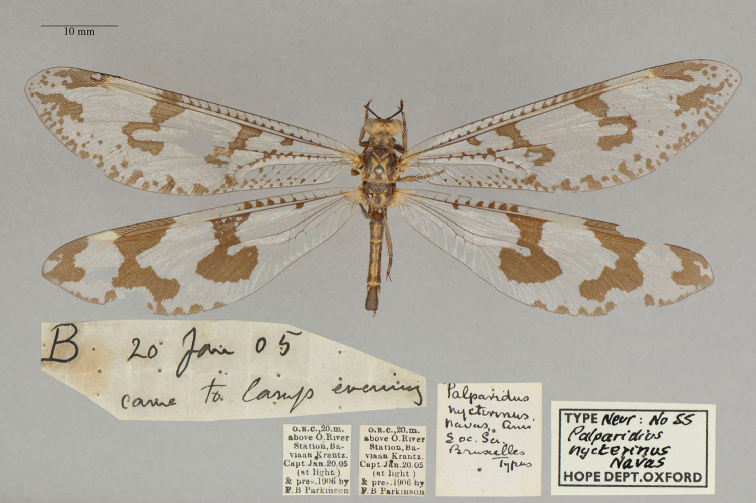
Holotype of *Palparidiusnycterinus* Navás, 1913 (NEUR0055, sex undetermined).

**12. *nycterinus* Navás, 1913** (*Palparidius*) (Holotype; Fig. 89)

**Original description.***Ann. Soc. sci. Bruxelles, 1913b, 37 (pt. 1): 90 (fig. 3).* “Zambèse. O. R. C. à 20 milles au-dessus du fleuve Orange, Station Baviaan Krantz, capturé à la lumière, 20 février 1905, F. B. Parkinson (Mus. d’Oxford).”; Footnote: “Le bout de l’abdomen manque. [The end of the abdomen is missing.]”. Sexes and number of specimens not specified.

**Type series.** The description contains measurements for one specimen and a footnote that implies Navás worked with one specimen from which the tip of the abdomen was missing. There is one type in the OUMNH (NEUR0055, Fig. 89), and the distal segments of its abdomen are missing (sex undetermined). We concur with Stange (2004: 36); it is a holotype (by implicit monotypy).

**Current name.***Palparidiuscapicola* Péringuey, 1910.

**Nomenclature.**Stange (2004: 36) and Oswald (2018) listed the synonymy without comment or reference. We did not find a reference for it elsewhere.

**Figure 90.**
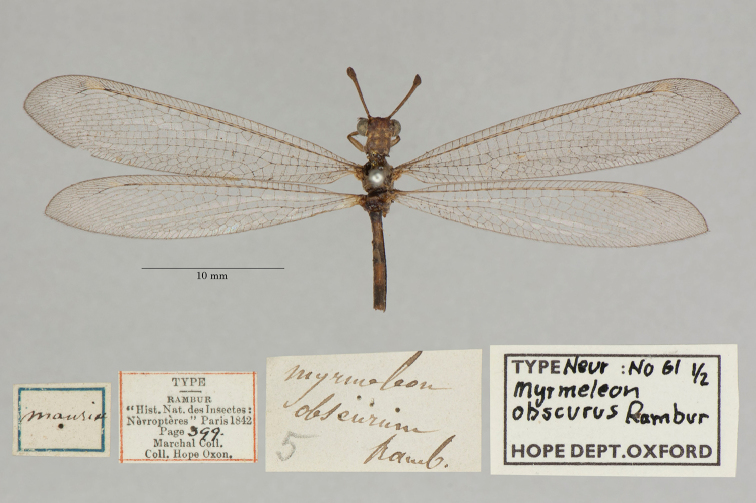
First of two OUMNH syntypes of *Myrmeleonobscurus* Rambur, 1842 (NEUR0061-1, sex undetermined).

**Figure 91.**
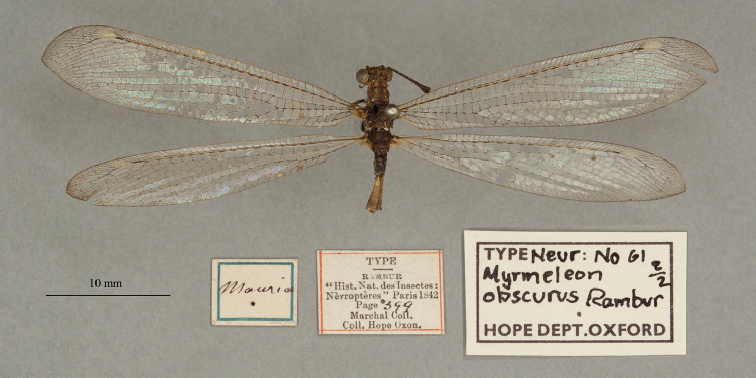
Second of two OUMNH syntypes of *Myrmeleonobscurus* Rambur, 1842 (NEUR0061-2, sex undetermined).

**13. *obscurus* Rambur, 1842** (*Myrmeleon*) (Two syntypes; Figs 90, 91)

**Original description.***Libr. encycl. Roret, 1842: 403.* “Habite l’Île Maurice; communiqué par M. Marchal.”. Sexes and number of specimens not specified.

**Type series.** Rambur did not indicate how many specimens he had. Stange (2004: 333) apparently did not see types for this species and questioned whether there were any in the IRSNB.

Two syntypes, sexes undetermined, are in the OUMNH; both are in fair condition and missing the distal segments of their abdomens (NEUR0061-01, -02; Figs 90, 91). Specimens of this species are also present in the NHMUK, but we did not identify any that would be considered types.

**Current name.***Myrmeleonobscurus* Rambur, 1842.

**Nomenclature.** The original name has remained unchanged; see Stange (2004: 333).

**Figure 92.**
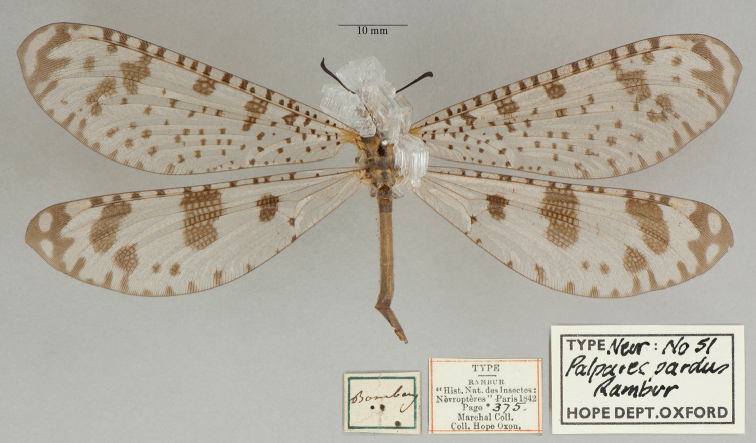
OUMNH syntype of *Palparespardus* Rambur, 1842 (NEUR0051, sex undetermined).

**14. *pardus* Rambur, 1842** (*Palpares*) (One syntype; Fig. 92)

**Original description.***Libr. encycl. Roret, 1842: 375.* “De Bombay.”. Sexes and number of specimens not specified.

**Type series.** Rambur mentioned features of both male and female specimens; thus it is clear that the type series had at least two specimens. Stange (2004: 40) indicated that he had seen male and female syntypes in the IRSNB, but he did not mention any types for this species in collections elsewhere. We found one Rambur type, sex undetermined, in the OUMNH (NEUR0051, Fig. 92) that we consider to be a syntype. In addition, numerous specimens are in the NHMUK, including several that are labeled as types (two with numbers NHMUK010288083, NHMUK010288084). Their type status should be examined before a lectotype is designated.

**Current name**: *Indopalparespardus* (Rambur, 1842).

**Nomenclature.** The genus name was reassigned by Insom and Carfì (1988: 77); see Stange (2004: 40).

**15. *poultoni* Navás, 1913** (*Cymothales*) (Holotype, not found)

**Original description.***Ann. Soc. sci. Bruxelles, 1913b, 91 (pt. 1): 91 (fig. 4).* “N. 2. Rhodesia, East Loangwa, Dist. 2400 feet, Petauke. 23 mars 1905, S. A. Neave, coll. (Mus. d’Oxford).”. Sexes and number of specimens not specified.

**Type series.** Navás did not mention explicitly how many specimens he used to prepare his original description. However, he noted only one depository, the OUMNH, and he provided measurements for only one specimen. Thus, Mansell (1987: 203) considered that Navás' description was based on a single type, the holotype. In any case, Mansell and we were unable to find a type for this species.

In an article that he wrote a year later, Navás (1914a: 113) referred to a specimen in the NHMUK as follows: “He visto otro ejemplar ♂ algo diferente del tipo un poco más obscuro. [I have seen another male specimen differing from the somewhat darker type.]”. Mansell (1987: 204) studied this specimen, a female labeled as a “cotype”; he reported that it and other specimens in the NHMUK identified by Navás have been useful in facilitating identification of the species. We suggest that one of these specimens be considered for designation as a neotype.

**Current name.***Cymothalespoultoni* Navás, 1913.

**Nomenclature.** The name of this species has not changed; see Mansell (1987: 203) and Stange (2004: 82).

**Figure 93.**
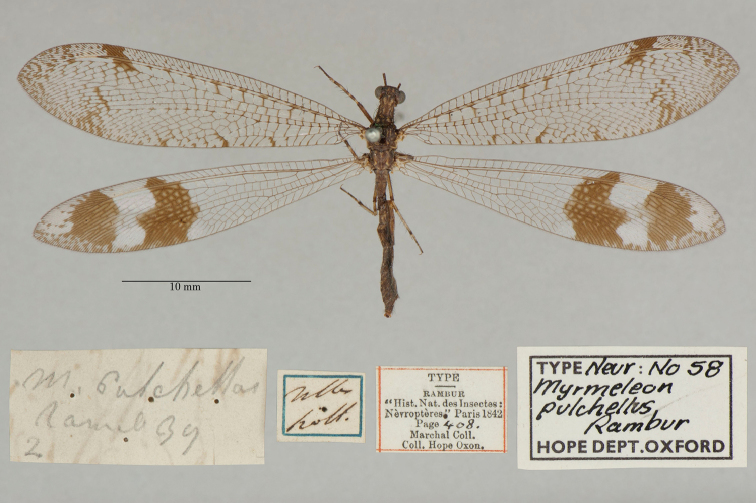
Holotype of *Myrmeleonpulchellus* Rambur, 1842 (NEUR0058, male).

**16. *pulchellus* Rambur, 1842** (*Myrmeleon*) (Holotype; Fig. 93)

**Original description.***Libr. encycl. Roret, 1842: 408.* “D’après un individu en assez nauvais état, venant de la Nouvelle-Hollande.”.

**Type series.** Rambur’s original description referred to a single specimen in rather poor condition; it did not indicate a sex or a depository. New (1985: 33) listed the “Types” as “?Paris, not seen”. Later, Stange (2004: 105) also listed the MNHN as the depository for the type of this species, but he did not report having seen it there, and neither did Miller and Stange (2012: 14). According to R. J. P. Machado (personal communication), the IRSNB reported a specimen that was considered the type. He examined an image of the specimen sent to him from the IRSNB and determined that it is not the type. It carries labels indicating that it was not associated with Rambur.

One specimen in the Hope Collection (NEUR0058, Fig. 93), a male, carries Rambur’s labels and is identified as a type. We consider it to be the *M.pulchellus* holotype (by explicit monotypy). The abdomen is damaged, and some legs are missing.

It is noteworthy that the NHMUK has a number of old *M.pulchellus* specimens from Australia (at least one with a Saunders label and some with McLachlan labels, including one with NHMUK catalog number NHMUK011250010). However, we could not confirm that Rambur had seen any of these specimens, and because he specifically mentioned only one specimen, we do not consider them to be part of the type series.

**Current name.***Glenoleonpulchellus* (Rambur, 1842).

**Nomenclature.** The generic reassignment was made by Banks (1913a: 224); also see Stange (2004: 105), Miller and Stange (2012: 14).

**Figure 94.**
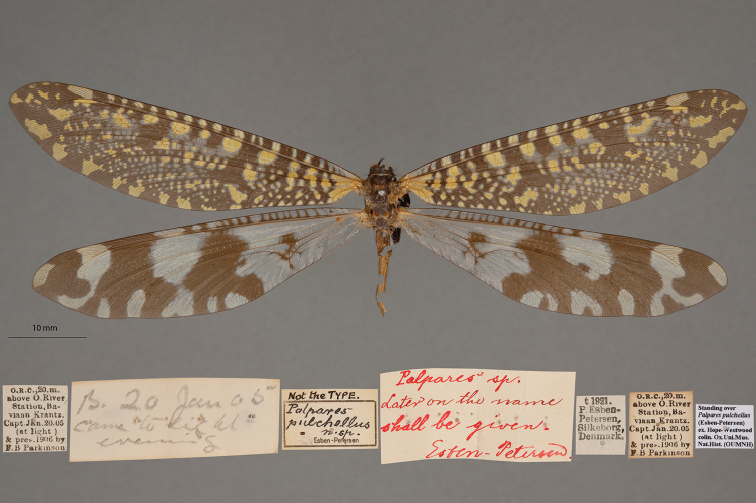
OUMNH paralectotype of *Palparespulchellus* Esben-Petersen, 1922 (NEUR0076, sex undetermined).

**17. *pulchellus* Esben-Petersen, 1922** (*Palpares*) (One paralectotype; Fig. 94)

**Original description.***Ann. Mag. Nat. Hist., 10: 618, fig. 2*; “1♂, 1♀, Deelfontein, South Africa (*Col. Sloggett*, 1903-109). Besides these two specimens, I have seen another specimen (head and abdomen lost) from Orange River Colony, 20 m. above Orange River Station, Baviaan Krantz, 20^th^ Jan., 1905 (at light) (*F. B. Parkinson* leg.).”. The caption on fig. 2 reads “… (from Baviaan Krantz: Oxford University Museum).”.

**Type series.** The description indicates that three specimens from two localities were in the type series; Esben-Petersen did not specify in his description which one he considered to be the holotype. Two of the types, the male and female from Deelfontein, South Africa, are in the NHMUK. Mansell (1996: 249) identified the male, which bears a “TYPE” label, as the holotype, and the female, which carries a “COTYPE” label, as a paratype (also see Stange 2004: 46). Given the information above, it appears that ICZN Article 74.5 applies in this case, and Mansell’s identification of the male in the NHMUK as the holotype fixes it as the lectotype. This type status is listed (with question) as such by Oswald (2018). The female specimen in the NHMUK is now a paralectotype.

We also found in the OUMNH the damaged specimen that Esben-Petersen reported from Orange River Colony and whose wings were shown in Fig. 2 of the original description (head and abdomen missing, sex undetermined). It was collected in 1905 and presented to Esben-Petersen in 1906; it carries labels that probably were applied as temporary labels early in his study. One of these labels states that the specimen is not the type. Clearly, however, from its inclusion with a figure in the original description, this specimen (NEUR0076, Fig. 94) was part of the type series. It is a paralectotype.

**Current name.***Palparelluspulchellus* (Esben-Petersen, 1922).

**Nomenclature.** The current combination was offered by Mansell (1996: 248); see Stange (2004: 46).

**Figure 95.**
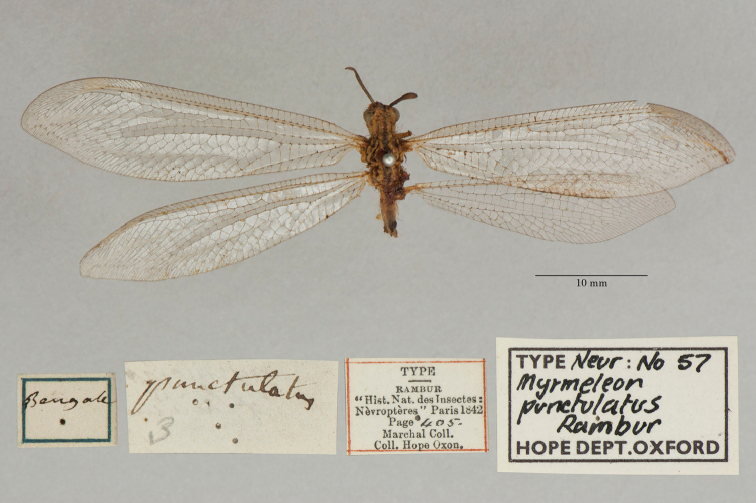
OUMNH syntype of *Myrmeleonpunctulatus* Rambur, 1842 (NEUR0057, sex undetermined).

**18. *punctulatus* Rambur, 1842** (*Myrmeleon*) (One syntype; Fig. 95)

**Original description.***Libr. encycl. Roret, 1842: 405.* “Communique par M. Marchal, et indiqué du Bengale.”. Sexes and number of specimens not specified.

**Type series.**Navás (1914b: 198) identified a specimen (with abdomen missing) in the MNHN that he thought might be the Rambur type. However, the presence of this specimen has not been confirmed, and Stange (2004: 291) stated that he had not located it in the collection.

A Rambur type with abdomen missing (sex undetermined) is in the OUMNH (NEUR0057, Fig. 95). It is from Bengal, and the identification label is consistent with Rambur’s handwriting.

Also, the NHMUK houses two specimens (NHMUK011250011, NHMUK011250012) with labels reading “Ind” and “E. Ind”. However, because there is no indication that Rambur saw these specimens, they do not appear to be types. We know of no published holotype identification or lectotype designation for this species. For now, until the MNHN and the NHMUK are searched thoroughly, the OUMNH specimen remains as the only confirmed syntype.

**Current name.***Cuetapunctulata* (Rambur, 1842).

**Nomenclature.** The generic name was reassigned by Navás (1914b: 198); see Stange (2004: 291).

**Figure 96.**
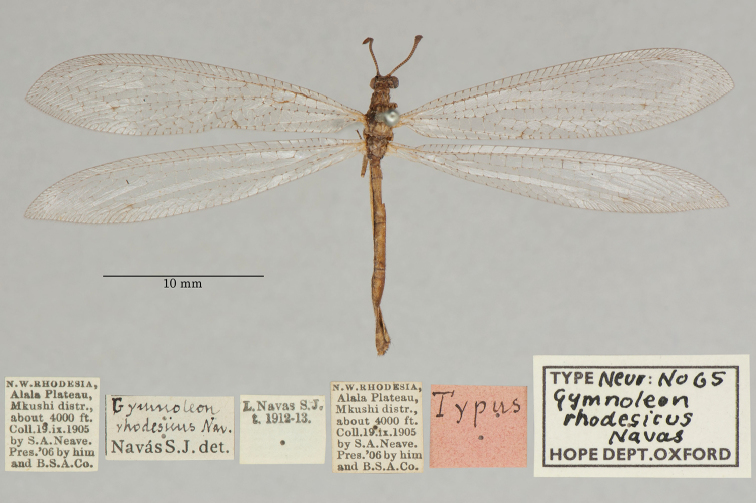
Holotype of *Gymnoleonrhodesicus* Navás, 1913 (NEUR0065, sex undetermined).

**19. *rhodesicus* Navás, 1913** (*Gymnoleon*) (Holotype; Fig. 96)

**Original description.***Mem. R. Acad. Cien. Artes Barcelona, 1913a, (3) 10: 488* “El rótulo resa: N. W. Rhodesia, Alala Plateau, Mkushi distr., about 4000 ft. Coll. 19-IX-1905 by S. A. Neave.”. Sexes and number of specimens not specified.

**Type series.** Although Navás did not state specifically how many specimens he examined, his reference to a “label” (singular) implies that he had only one. One specimen, sex undetermined, is present in the OUMNH (NEUR0065, Fig. 96), and we consider it to be the holotype (by implicit monotypy). Its locality label conforms with the type locality, and it carries Navás’ identification and type labels.

**Current name.***Gymnoleonrhodesicus* Navás, 1913.

**Nomenclature.** The original name has remained unchanged; see Stange (2004: 181).

**Figure 97.**
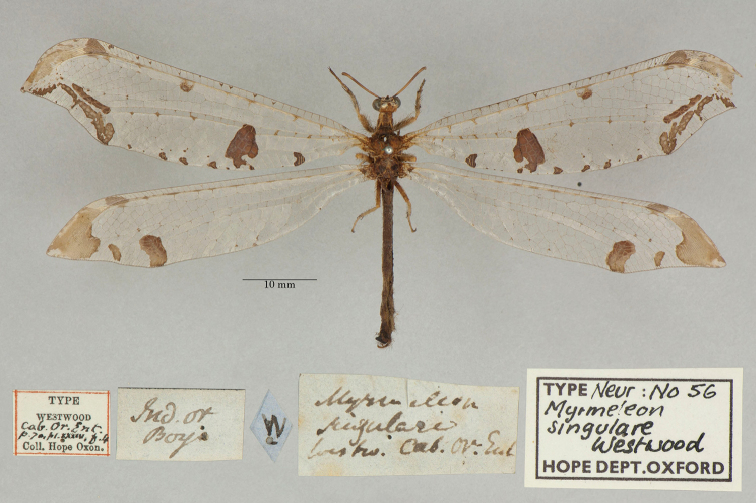
Lectotype (new designation) of *Myrmeleonsingulare* Westwood, 1847 (NEUR0056, male).

**20. *singulare* Westwood, 1847** (*Myrmeleon*) (Lectotype, new designation; Fig. 97)

**Original description.***The Cabinet of Oriental Entomology; being a selection of the rarer and more beautiful species of insects, natives of India and the adjacent islands. The greater portion of which are now, for the first time, described and figured. Smith, London, 1848 [1847]: 70, Plate 34, fig. 4.* “Inhabits the East Indies. In the Collection of the Linnaean Society and Capt. Boys.”. Sexes and number of specimens not specified.

**Type series.** Westwood did not mention how many specimens he had. Stange (2004: 92) suggested that the type was in the OUMNH, but he did not indicate that he had seen it.

A Westwood type (male), bearing labels in Westwood’s handwriting with data that are consistent with the original description, is in the OUMNH (NEUR0056, Fig. 97). Its labels also indicate an association with the Cabinet of Oriental Entomology and the collection of Captain Boys. There is another potential syntype (NHMUK011250009) in the NHMUK; it bears a Westwood identification label and a Saunders label. However, its status as a type is not as well supported as that of the OUMNH type. Given the clarity of the data associated with the OUMNH specimen (NEUR0056), here we designate it as the lectotype (present designation).

**Current name.***Indoclystussingularis* (Westwood, 1847).

**Nomenclature.** The current combination is by Banks (1941: 3); cited by Stange (2004: 92, as *singulare*) and Oswald (2018, as *singularis*).

The original specific name was “*singulare*” (Latin, third declension, neuter) in gender agreement with the genus name when most early systematists (e.g., Linnaeus, Fabricius, and also Westwood) treated *Myrmeleon* as neuter. Other, mostly later, authors (e.g., Walker 1853: 399, Banks 1941: 3, New 1985: 3, Ghosh 2000: 75) identified the genus name *Myrmeleon* as masculine and used the masculine “*singularis*”. The genus name *Indoclystus* also is masculine; thus, the species name *singularis* is in agreement.

**Figure 98.**
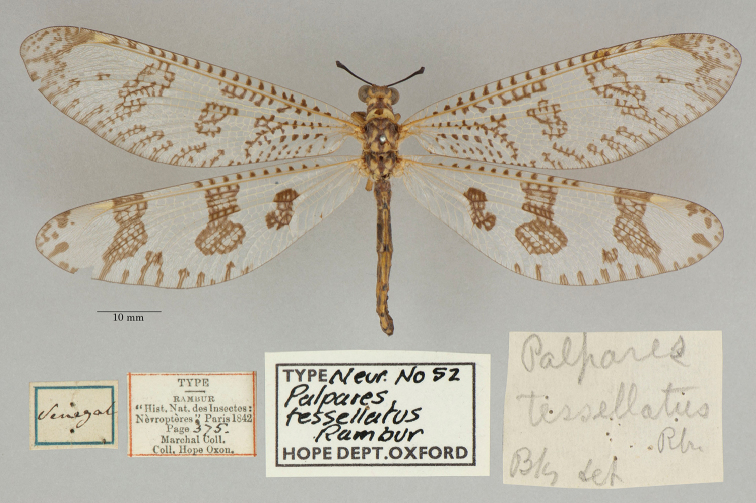
OUMNH paralectotype of *Palparestessellatus* Rambur, 1842 (NEUR0052, female).

**21. *tessellatus* Rambur, 1842** (*Palpares*) (One paralectotype; Fig. 98)

**Original description.***Libr. encycl. Roret, 1842: 375*. “Du Sénégal. La femelle communiqué par Marchal.”.

**Type series.** Rambur specifically mentioned seeing at least two specimens, a male and a female. The female (from Senegal) was given to him by Marchal; this specimen is in the OUMNH (NEUR0052, Fig. 98). In addition, several other syntypes that Rambur studied have been found. Prost (1995: 97) listed four specimens in the IRSNB; he designated one of them (a male) as the lectotype. He also mentioned the specimen in the OUMNH. Stange (2004: 58, as *tesellatus*) cited the lectotype designation, but he did not indicate that he had seen the specimen. We also found two other types in the NHMUK collection (NHMUK10288072, NHMUK10288073); they too are now paralectotypes.

**Current name.***Palparespercheronii* (Guérin-Méneville, 1831).

**Nomenclature.** The species was synonymized by Fraser (1950: 115, as *P.tesselatus* and *P.percheroni*, both incorrect subsequent spellings); it was confirmed by Prost (1995: 96) but with the priority reversed, because he believed that the publication of the pages containing the *P.percheronii* description had been delayed until 1844. However, Stange (2004: 59, as *tesellatus*) indicated that the earlier publication date (1831) of Fig. 62 under the species’ name *Myrmeleonpercheronii* has precedence over the publication date of the description. And, thus *P.percheronii*, with its publication date of 1831 has priority over *P.tessellatus* Rambur, 1842.

It should be noted that at least two subsequent misspellings are associated with this species name: *tesselatus* by Fraser (1950: 115) and *tesellatus* by Stange (2004: 59).

**Figure 99.**
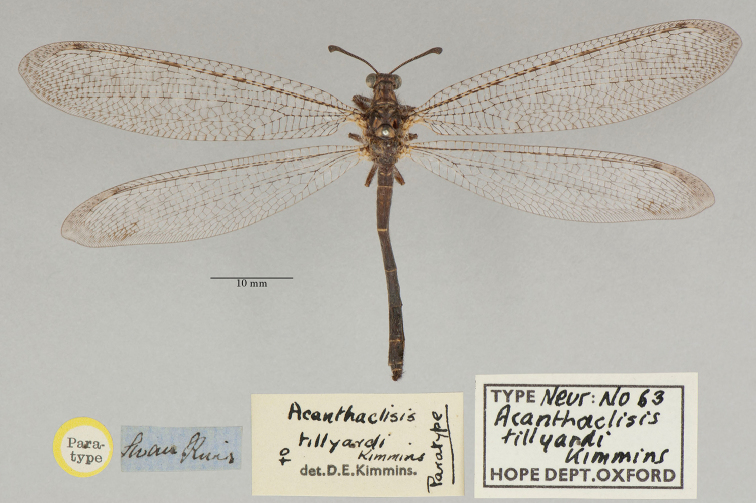
OUMNH paratype of *Acanthaclisistillyardi* Kimmins, 1939 (NEUR0063, female).

**22. *tillyardi* Kimmins, 1939** (*Acanthaclisis*) (One paratype; Fig. 99)

**Original description.***Ann. Mag. Nat. Hist., 1939: 588, Plate 19, Fig. b.* “W. AUSTRALIA: type ♂, Yanchep, 32 miles N. of Perth, 9–23. i. 36 (*Miss Raymond*), in the British Museum; paratype ♀, Swan River, in the Hope Department, University Museum, Oxford.”.

**Type series.** The holotype (by original designation) is in the NHMUK (Stange 2004: 353); it is reported in the NHMUK Database (NHMUK010288462). Kimmins’ paratype female is in the OUMNH (NEUR0063, Fig. 99).

**Current name.***Heoclisistillyardi* (Kimmins, 1939).

**Nomenclature.** The current generic assignment was made by New (1985: 56); see Stange (2004: 168).

#### Nemopteridae (Spoon-winged or thread-winged lacewings)

This relatively small family (~150 described species) contains adults with highly modified and striking hindwings and equally remarkable larvae with extreme thoracic elongation. Nemopterids are restricted to arid desert and dry tropical regions of northern and southern Africa, southern Europe, the Middle East, southern Asia, Australia, and southern South America (Tjeder 1967, Hölzel 1975). Currently, the family contains two distinct lineages, which are represented by two subfamilies, Nemopterinae and Crocinae. Comprehensive systematic studies of the group are those by Tjeder (1967) on the rich fauna of southern Africa, and Hölzel (1975) on the Crocinae. In our treatment here, we provide current nomenclatural information, and we cite (but do not repeat) the synonymies of the species included in the above works.

The OUMNH holds primary types for all seven nemopterid species reported to have types in the collection (three with holotypes, one with a lectotype, and three with syntypes). The specimens include representatives from both subfamilies. All but two of the species were described by Westwood; Navás and Withycombe each described one.

**Figure 100.**
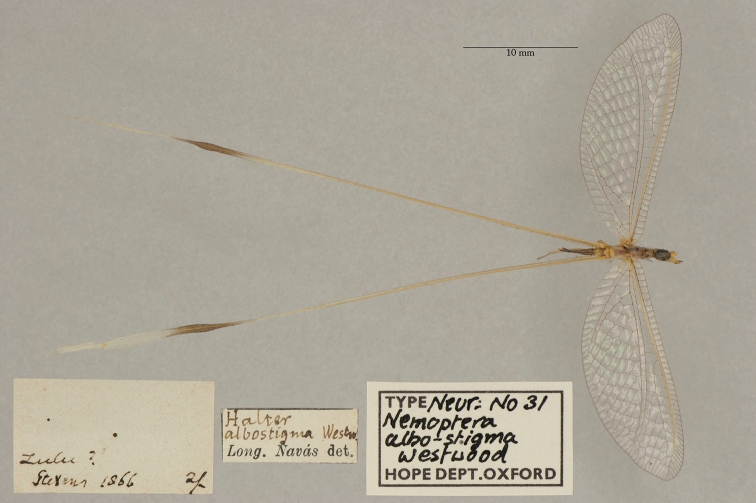
Holotype of *Nemopteraalbostigma* Westwood, 1874 (NEUR0031, probably male).

**Figure 101.**
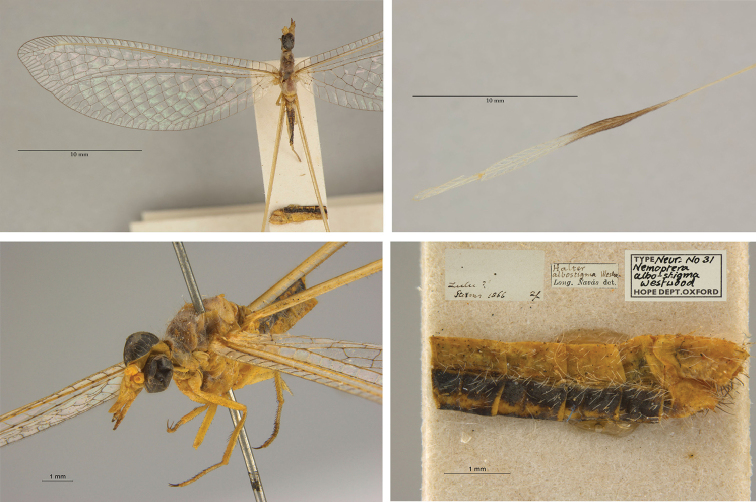
Holotype of *Nemopteraalbostigma* Westwood, 1874 (NEUR0031, probably male).

**1. *albostigma* Westwood, 1874** (*Nemoptera*, as “*albo-stigma*”) (Holotype; Figs 100, 101)

**Original description.***Thesaurus Entomologicus Oxoniensis; or, illustrations of new, rare, and interesting insects, for the most part contained in the collections presented to the University of Oxford by the Rev. F. W. Hope ... . Clarendon Press, Oxford, p. 179, Plate XXXIII (= 33), fig. 7.* “Africa australior, Terra Zoolu. In Mus. Hopeiano Oxoniae.”. Sexes and number of specimens not specified.

**Type series.** One type, probably a male, is in the OUMNH (NEUR0031, Figs 100, 101). It carries a locality label reading “Zulu”, as in the original description, and an identification label by L. Navás with the name “*Halteralbostigma* Westw.” Because only one depository is mentioned in the original description and this specimen is the only type of this species in the collection, we consider it to be the holotype (by implicit monotypy).

**Current name.***Halteralbostigma* (Westwood, 1874).

**Nomenclature.** The current combination was proposed by Kirby (1900: 459).

**Figure 102.**
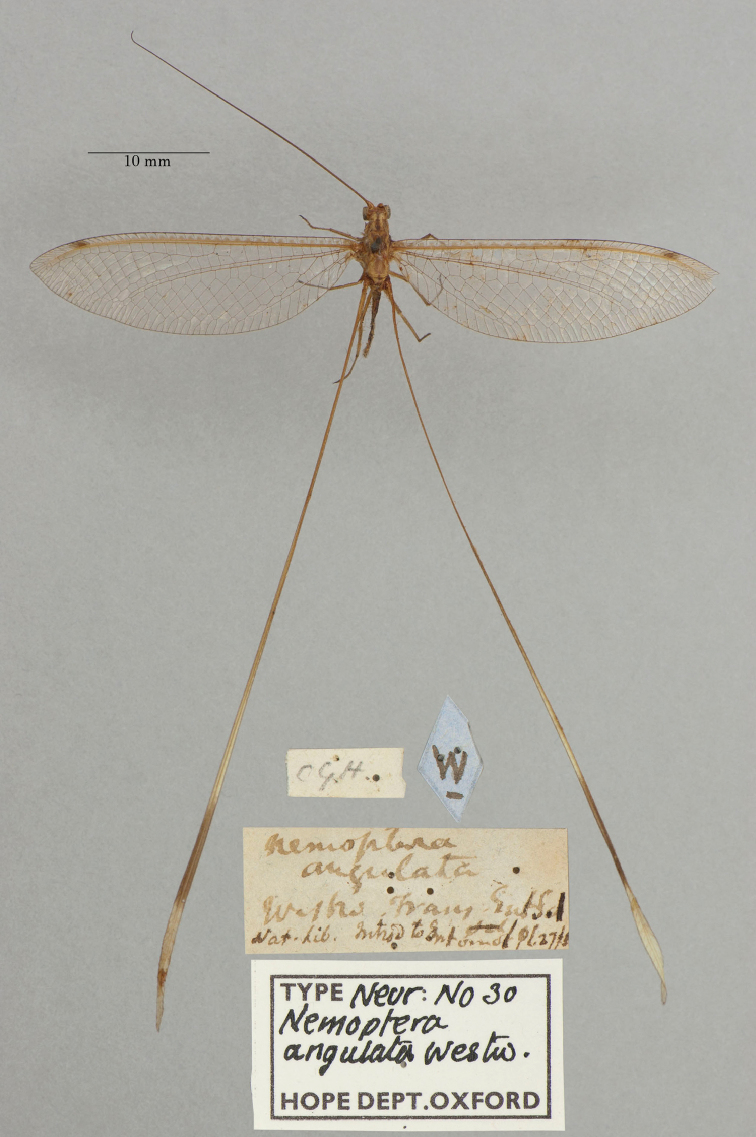
Holotype of *Nemopteraangulata* Westwood, 1836 (NEUR0030, male).

**2. *angulata* Westwood, 1836** (*Nemoptera*) (Holotype; Fig. 102)

**Original description.***Trans. R. Entomol. Soc. Lond., 1*: lxxv [= 75]. “Cape of Good Hope.”. Sexes and number of specimens not specified.

**Type series.** Westwood’s original description consists of a very brief note of three lines from an exhibit at the Entomological Society of London. This description of *N.angulata* follows a similarly abbreviated description of *Nemopteracostalis* Westwood, and it ends with the question “(An mas praecedentis?) [Is it a male of the former?]”. Thus, it appears that Westwood had one male specimen of *N.angulata*, and that he was not certain if the species differed from *N.costalis*.

Later, Tjeder (1967: 446) declared that the two species are distinct, and he redescribed them both. Although he did not examine the type specimen of *N.angulata*, he had a photograph of it that he compared with other material from Cape Province. He identified Westwood’s specimen of *N.angulata* in the OUMNH, a male, as the holotype. The specimen (NEUR0030, Fig. 102) is the only type of this species in the collection, and we have no reason to alter Tjeder’s determination of it as the holotype. Oswald (2018) lists it as the holotype (by implicit monotypy).

**Current name.***Nemiaangulata* (Westwood, 1836).

**Nomenclature.** Several authors after Westwood considered *N.angulata* and *N.costalis* to be synonymous. A full synonymy and the first use of the current combination were published by Tjeder (1967: 442–443, 446); also listed by Mansell (2000: 174).

**Figure 103.**
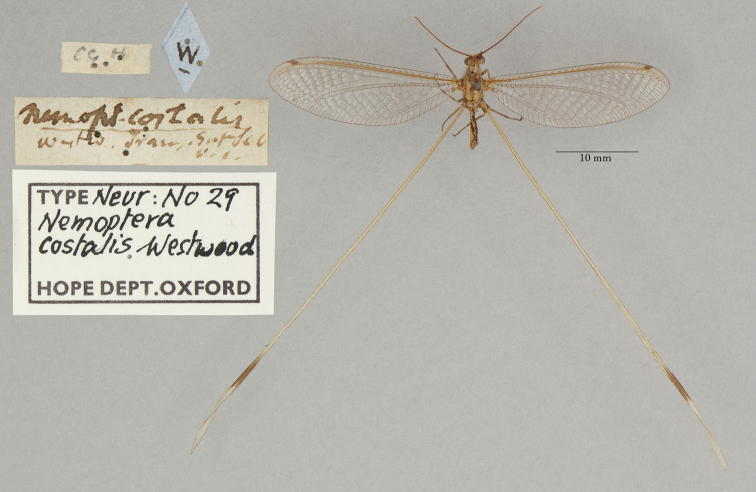
Holotype of *Nemopteracostalis* Westwood, 1836 (NEUR0029, female).

**3. *costalis* Westwood, 1836** (*Nemoptera*) (Holotype; Fig. 103)

**Original description.***Trans. R. Entomol. Soc. Lond., 1*: lxxv [= 75]. “Cape of Good Hope.”. Sexes and number of specimens not specified.

**Type series.** Neither Westwood's very brief original description nor his later redescription (Westwood 1874: 179) provides specific information on the number of types that he studied. However, they do offer some evidence that supports Tjeder’s (1967: 443) identification of the single specimen in the OUMNH (NEUR0029, Fig. 103) as the holotype. First, Westwood’s redescription of *N.costalis* cites only one depository, "Mus. Hopeiano Oxoniae". Second, his question concerning the identity of the *N.angulata* male (see above) implies that he had no males of *N.costalis*. As a consequence, Tjeder's identification of the type in the OUMNH as a female supports its status as the holotype. Given the absence of other *N.costalis* types in the collection, we see no reason to alter the holotype determination. Oswald (2018) also lists it as the holotype (by implicit monotypy).

**Current name.***Nemiacostalis* (Westwood, 1836).

**Nomenclature.** The first use of the combination was by Navás (1915b: 36). Several authors identified the holotype of this species and the holotype of *N.angulata* Westwood to be a male and female of the same species (see above), but Tjeder (1967: 442–444) redescribed each as fully distinct species.

**Figure 104.**
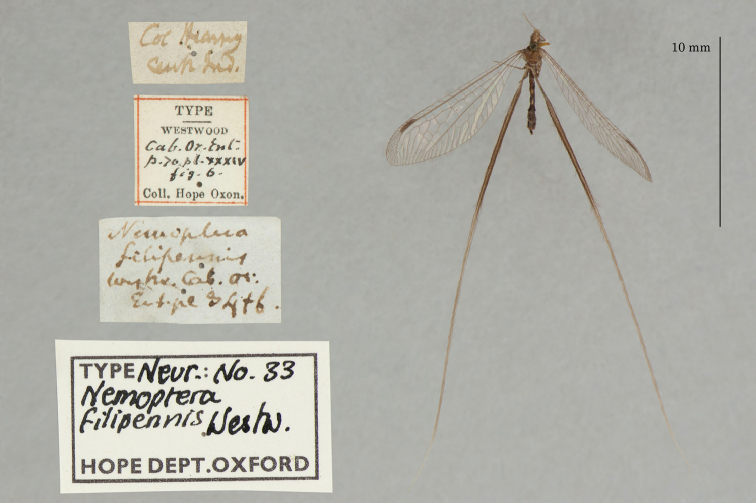
OUMNH syntype of *Nematopterafilipennis* Westwood, 1841 (NEUR0033, sex undetermined).

**4. *filipennis* Westwood, 1841** (*Nematoptera*) (One syntype; Fig. 104)

**Original description.***Proc. Zool. Soc. Lond., 9: 13.* “Habitat in India orientali. In Mus. D. W. W. Saunders, F.L.S., &c.”. Sexes and number of specimens not specified.

**Type series.** Although Westwood’s very brief original description carried no mention of the number of specimens he had, his use of the term “&c” after the Saunders collection implies that in addition to the one in the OUMNH, types may exist elsewhere. His slightly fuller redescription later (Westwood 1848: 70, as “*Nemoptera*”) indicated two depositories, “the Collections of Col. Hearsey and Mr. Hope.” Hölzel (1975: 60) reported the “Holotypus in Mus. Oxford, nicht gesehen [not seen]”.

A single syntype, sex undetermined, is in the OUMNH (NEUR0033, Fig. 104). No lectotype has been designated.

**Current name.***Crocefilipennis* (Westwood, 1841).

**Nomenclature.** The current combination was proposed by McLachlan (1885: 378). The species was also listed by Oswald (2018) as *Himantopterusfilipennis* (Westwood), but we did not find a citation in support of this name. See a list of synonymies by Hölzel (1975: 60, as *Croce*).

**Figure 105.**
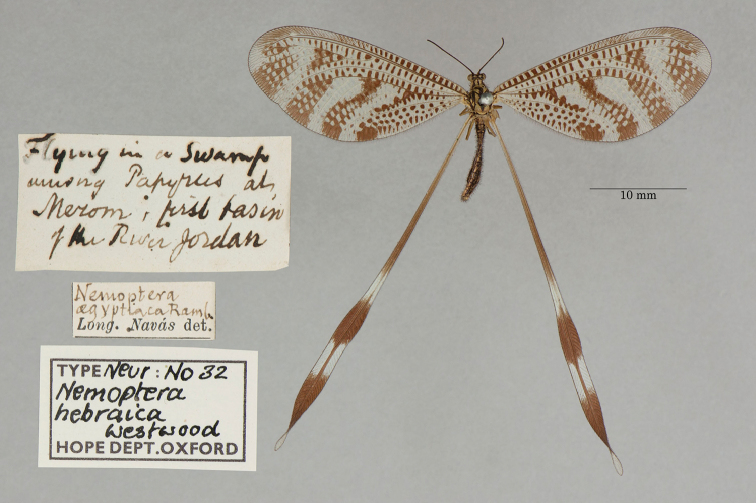
OUMNH syntype of *Nemopterahebraica* Westwood, 1874 (NEUR0032, probably male).

**5. *hebraica* Westwood, 1874** (*Nemoptera*) (One syntype; Fig. 105)

**Original description.***Thesaurus Entomologicus Oxoniensis; or, illustrations of new, rare, and interesting insects, for the most part contained in the collections presented to the University of Oxford by the Rev. F. W. Hope ... . Clarendon Press, Oxford. P. 178, Plate XXXIII = 330, fig. 5.* “Northern Palestine. ‘Flying in a swamp among papyrus, near the waters of Merom, forming the first basin of the River Jordan,’ – Rev. D. D. Holland and Pickard Cambridge [O. Pickard-Cambridge]. In Mus. Hopeiano Oxoniae.”. Sexes and number of specimens not specified.

**Type series.** Currently, there is one type, probably a male, in the OUMNH (NEUR0032, Fig. 105). In the absence of information concerning the number of specimens in the type series, we consider it a syntype. The specimen carries an identification label by L. Navás with the name “*Nemopteraaegyptiaca* Ramb.”.

**Current name.***Nemopteraaegyptiaca* Rambur, 1842.

**Nomenclature.** The synonymy between *N.hebraica* and *N.aegyptiaca* was suspected in the original description (Westwood 1874: 178), confirmed by McLachlan (1885: 379), and listed by Navás (1910: 357).

**Figure 106.**
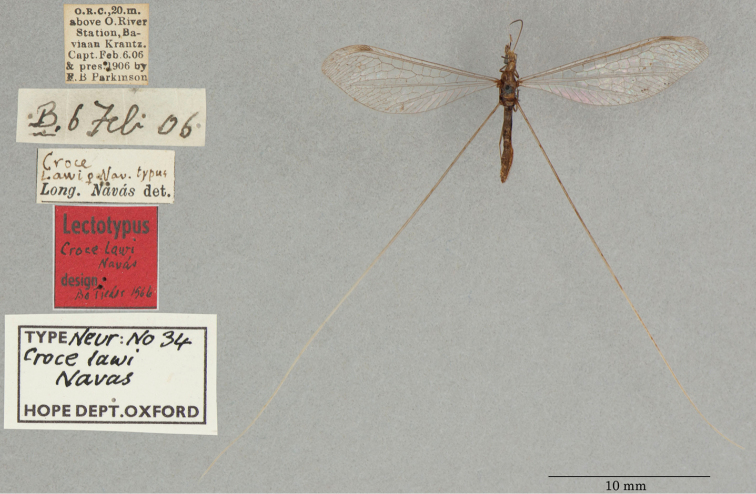
Lectotype of *Crocelawi* Navás, 1913 (NEUR0034, female).

**6. *lawi* Navás, 1913** (*Croce*) (Lectotype; Fig. 106)

**Original description.***Ann. Soc. sci. Bruxelles, 37 (pt. 1) [1913b]: 87, fig. 1.* “Afrique, Zambèse. Above O. River. Station Raviaan Krantz, 6 févr., 1906 (Mus. d’Oxford).”.

**Type series.** Navás mentioned a female, but he did not state how many specimens he studied. Tjeder (1967: 329) designated the single specimen (a female) in the OUMNH as the lectotype (NEUR0034, Fig. 106). He also discussed inaccuracies in Navás’ report of the type locality (Tjeder 1967: 330).

**Current name.***Laurhervasiasetacea* (Klug, 1838).

**Nomenclature.** The synonymy, current combination, and history of name changes are found in Tjeder (1967: 325–6, 329).

**Figure 107.**
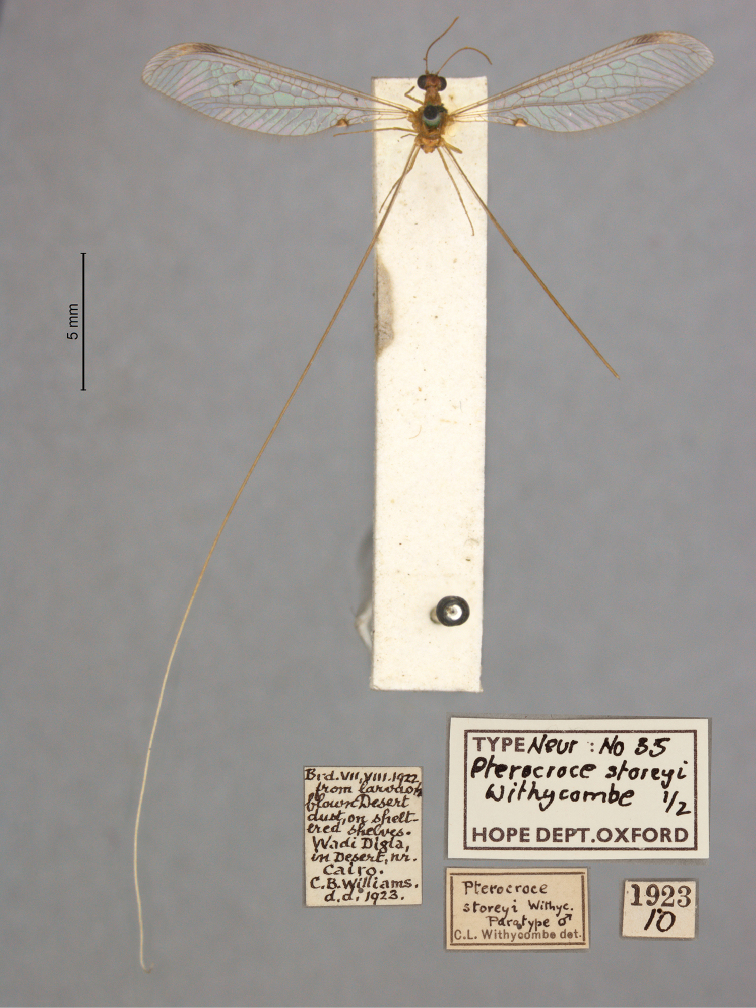
First of two OUMNH syntypes for *Pterocrocestoreyi* Withycombe, 1923 (NEUR0035-01, male).

**Figure 108.**
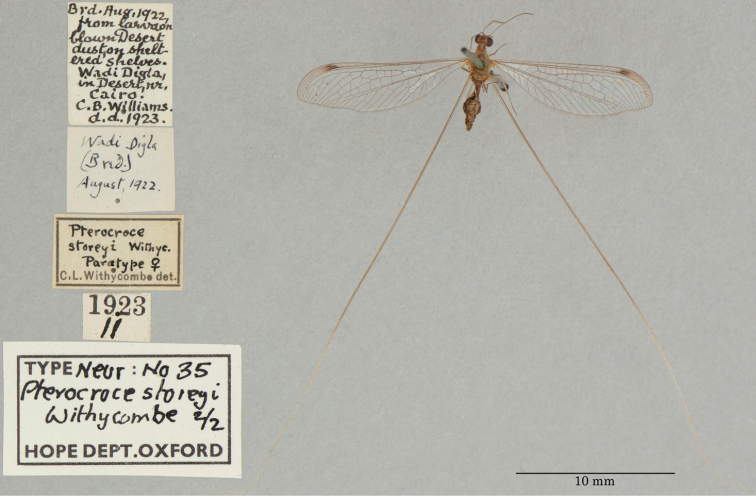
Second of two OUMNH syntypes for *Pterocrocestoreyi* Withycombe, 1923 (NEUR0035-02, female).

Note: All of the adult specimens that Withycombe studied were reared from field-collected larvae; the larvae also were described in the second article (Withycombe 1923–1924: 279). No mention is made of whether he preserved any larval specimens.

**7. *storeyi* Withycombe, 1923** (*Pterocroce*) (Two syntypes; Figs 107, 108)

**Original description.***Entomol., 56: 141.* “*Hab.* – Cairo, Egypt.”.

**Type series.** The extremely brief original description, which mentioned both male and female specimens, soon was followed by a more detailed redescription (Withycombe 1923–1924: 277). This later article mentions: a “Type ♂” and a “Type ♀” in the NHMUK, a male “paratype” and a female “paratype” in the Oxford Collection, and a male “paratype” and a female “paratype” in Withycombe’s personal collection (total of six specimens). It is clear that Withycombe regarded two specific types in the NHMUK as the primary types; however, he did not specify which should be the name-bearing type, the male or the female. Indeed, his entire article treats male and female specimens in a balanced manner.

Withycombe’s identification of two specimens as “Type” precludes the application of ICZN Article 74.5, and all of the specimens he used in his type series remain as syntypes -- including the four specimens mentioned in the original description as “paratypes”, and any additional adult or larval specimens that can be shown to have been included as part of the type series (ICZN Article 73.2). Thus, at this time, the two specimens in the OUMNH (a male with abdomen missing, NEUR0035-01, Fig. 107; and a female, NEUR0035-02, Fig. 108) are considered to be syntypes.

**Current name.***Necrophylusarenarius* Roux, 1833.

**Nomenclature.** The original name was synonymized with *Pterocrocecapillaris* (Klug) by Hölzel (1975: 80) and subsequently synonymized with *Necrophylusarenarius* Roux by Monserrat (2008: 20).

### Raphidioptera

#### Raphidiidae (Snakeflies)

The Raphidiidae is one of two families that comprise the order Raphidioptera. The entire order is small (~250 species), and its distribution and fossil record indicate that it is a relictual group. The family Raphidiidae is restricted to woodland habitats in the northern hemisphere, with species having quite limited distributions, largely within cool climates (Aspöck and Aspöck 2009: 864).

Both adults and larvae of this group are predaceous. Larvae are terrestrial and live beneath bark or in the soil. Little is known of their feeding habits and other biological traits.

The OUMNH houses the lectotype and paralectotype of one snakefly species. The species was described by Navás.

**Figure 109.**
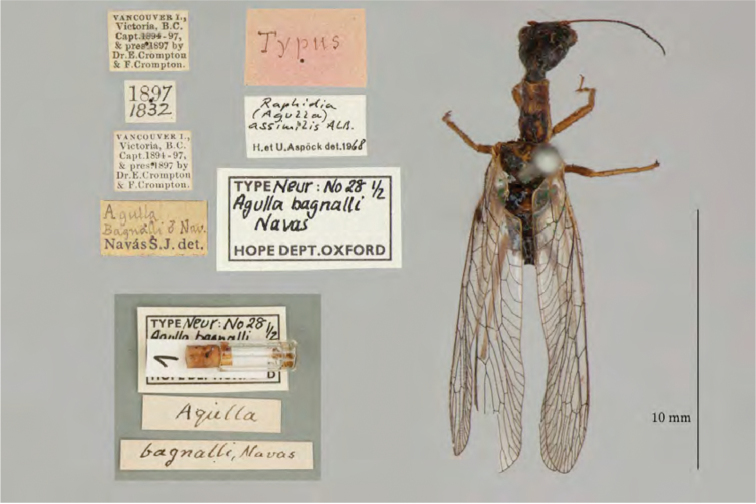
Lectotype of *Agullabagnalli* Navás, 1914 (NEUR0028-01, male).

**Figure 110.**
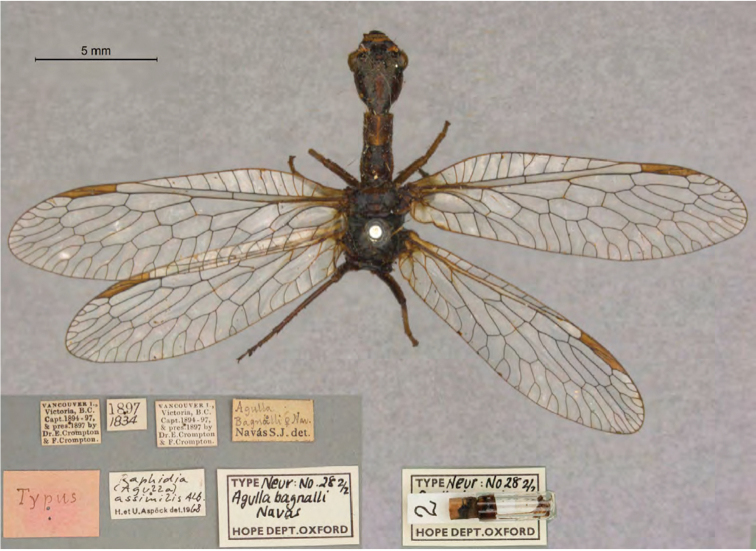
OUMNH paralectotype of *Agullabagnalli* Navás, 1914 (NEUR0028-02, female).

**1. *bagnalli* Navás, 1914** (*Agulla*) (Lectotype, paralectotype; Figs 109, 110)

**Original description.***Bol. Soc. Aragonesa Cienc. Nat., 1914c, 13: 67–68, fig. 2.* “Oceania: ‘Vancouver J. [I.?], Victoria, B. C. 1894–97,’ Dr. E. Crompton (Mus. De Oxtord [Oxford]).”.

**Type series.** In his original description, Navás mentioned male and female features. Two types (male and female) are in the collection, each with a type label in Navás’ handwriting and his determination label with either a male or female symbol. He did not specify which was to be the name-bearing type. The genitalia of both specimens are dissected and held in microvials with glycerol.

Carpenter (1936: 128) referred to the *A.bagnalli* type twice: (i) On page 127, he listed it as “*Holotype* (♀). -- …”. (ii) Then, on page 128, it appears that he mistakenly assumed that Navás had only one specimen, a male, and his discussion refers only to the male specimen or the male type. Thus, with regard to name-bearing status, Carpenter did not explicitly distinguish between the male and the female specimens. Subsequently, Penny et al. (1997: 90) referred to the “Holotype male: … (Oxford)”. This statement fixes the male type in the OUMNH (NEUR0028-01, Fig. 109) as the lectotype (ICZN Article 74.5); the female becomes a paralectotype (NEUR0028-02, Fig. 110).

**Current name.**Agulla (Agulla) assimilis (Albarda, 1891).

**Nomenclature.** Shortly after his description was published, Navás (1917: 26) realized that the type locality of *A.bagnalli* was not in Oceania, but in Victoria, Vancouver Island, British Columbia. As a result, he then synonymized this species with *Agullaassimilis* (Albarda), a species that typically occurs in British Columbia [see Carpenter (1936: 128), Penny et al. (1997: 90)].

## Appendix

With one exception, types of all the neuropteran species that were reported by Westwood (1848) [1847] in the Cabinet of Oriental Entomology are found in the OUMNH. The exception is *Ascalaphuscanifrons* Westwood, 1847. Its types are in the NHMUK. Our notes follow.

***canifrons* Westwood, 1847** [Ascalaphus (Bubo)]

**Original description.***The Cabinet of Oriental Entomology; being a selection of the rarer and more beautiful species of insects, natives of India and the adjacent islands. The greater portion of which are now, for the first time, described and figured. Smith, London, 1848 [1847]: 69, fig. 3*. “Inhabits the East Indies. In the Collection of W. W. Saunders, Esq.”. Sexes and number of specimens not specified.

**Type series.** The depository for the type(s) of this species was not reported in the original description. Moreover, we have found no reports of the species in the OUMNH collection, and we did not find any specimens there. Thus, we assume that types of *A.canifrons* never were in the collection. There are several specimens of this species in the NHMUK collection; we confirmed one type (NHMUK010212098, sex undetermined).

**Current name.***Ascalaphodescanifrons* (Westwood, 1847)

**Nomenclature.** Shortly after its original description, the species was referred to as *Bubocanifrons* (Westwood) [combination by Hagen (1866: 382)]. Later it was transferred to its current genus, *Ascalaphodes* [combination by McLachlan (1873a: 272)].
